# Defining a COPD composite safety endpoint for demonstrating efficacy in clinical trials: results from the randomized, placebo-controlled UPLIFT® trial

**DOI:** 10.1186/s12931-016-0361-4

**Published:** 2016-05-04

**Authors:** Bartolomé R. Celli, Marc Decramer, Dacheng Liu, Norbert Metzdorf, Guus M. Asijee, Donald P. Tashkin

**Affiliations:** Pulmonary Division, Brigham and Women’s Hospital, 75 Francis Street, PBB Clinics 3, Boston, MA 02115 USA; University of Leuven, Leuven, Belgium; Boehringer Ingelheim Pharmaceuticals Inc, Ridgefield, CT USA; Boehringer Ingelheim Pharma GmbH & Co. KG, Ingelheim am Rhein, Germany; David Geffen School of Medicine UCLA, Los Angeles, CA USA

**Keywords:** Exacerbation, COPD, Mortality, Composite endpoint, Outcomes, Discontinuation, Safety

## Abstract

**Background:**

Chronic obstructive pulmonary disease (COPD) clinical trials evaluating hard endpoints (mortality, hospitalized exacerbations) require a large number of subjects and prolonged observational periods. We hypothesized that a composite endpoint of respiratory outcomes (CERO) can help evaluate safety and benefit in COPD trials.

**Methods:**

Retrospective analysis of 5992 patients enrolled in the 4-year UPLIFT® trial, a randomized trial of tiotropium versus placebo in patients with moderate-to-severe COPD. Patients were permitted to continue using their usual COPD medications except for other anticholinergics. The CERO included deaths, respiratory failure, hospitalized exacerbations, and trial dropout due to COPD worsening. The incidence rates (IRs) per 100 patient-years and risk ratios (RRs and 95 % CI) were determined at years 1 to 4. The effect of treatments on CERO was similarly assessed. A power analysis helped calculate the sample size needed to achieve outcome differences between treatments.

**Results:**

The CERO IRs at years 1 to 4 for tiotropium versus placebo were 16, 13, 11, and 11, and 21, 16, 14, and 13, respectively. The RRs of CERO between tiotropium and placebo at the same time points were: RR-year 0.76 (0.67, 0.86), 0.80 (0.72, 0.88), 0.81 (0.74, 0.89), and 0.84 (0.77, 0.92). Using the IRs and RRs, the sample size (alpha = 0.05 two-sided, 90 % power) for studies of 1, 2, 3, and 4 years would be 1546, 1392, 1216, and 1504 per treatment group, respectively, with 575, 810, 930, 1383 required events, respectively, for hypothetical, event-driven studies.

**Conclusions:**

A composite endpoint incorporating relatively infrequent serious or significant COPD-related safety outcomes could be useful in clinical trials. In UPLIFT®, CERO events were significantly reduced in patients receiving tiotropium compared with placebo.

**Trial registration:**

NCT00144339.

**Electronic supplementary material:**

The online version of this article (doi:10.1186/s12931-016-0361-4) contains supplementary material, which is available to authorized users.

## Background

Chronic obstructive pulmonary disease (COPD) clinical trials evaluating relatively infrequent endpoints such as mortality, respiratory failure, and/or hospitalized exacerbations are, of necessity, often large and require follow-up of patients over prolonged periods of time [1–3]. Indeed, recent trials evaluating mortality as the outcome, such as the Tiotropium Safety and Performance in Respimat® (TIOSPIR®) study [3] or the Study to Understand Mortality and Morbidity in COPD (SUMMIT) [4], recruited more than 16,000 patients in order to have sufficient power to determine the significance of the outcomes.

Facing a similar situation, studies in other medically relevant fields have integrated infrequently occurring events into composite endpoints that have proven relevant to the planning of clinical trials and the likely benefit of treatment to patients [5–9]. This is particularly relevant in the field of cardiology, where a composite measure of major adverse cardiac events (MACE) has become a frequent endpoint for therapeutic trials [5, 6, 10]. Composite endpoints have also been used in infectious diseases (including tuberculosis [7]), in stroke [11], collagen vascular disease [12], multiple sclerosis [9], diabetes [8], and renal failure [6]. In COPD, a composite endpoint for safety in an endobronchial valve trial for emphysema was evaluated [13].

Using data from a large-scale clinical trial in patients with moderate to very severe COPD [2], we explored three hypotheses. First, that four important individual outcomes in COPD—death, hospitalized exacerbations, respiratory failure, and withdrawal from the study due to worsening COPD—could be integrated into a composite endpoint. Second, that there are measurable differences in the composite endpoint incidence between an active and a control arm. And third, that based on these results, fewer patients would need to be recruited to achieve sufficient power to explore significant effects of interventions on these outcomes. These hypotheses were tested using data from the UPLIFT® (Understanding Potential Long-term Impacts on Function with Tiotropium) trial, which included patients with COPD who were permitted to continue using their usual COPD medications, except for other inhaled anticholinergics [2].

## Methods

UPLIFT® (ClinicalTrials.gov number, NCT00144339) was an international, multicenter, 4-year, randomized, placebo-controlled trial of tiotropium in patients with COPD, the details of which were previously published [2, 14, 15]. Patients were recruited from 490 centers in 37 countries. In brief, patients were aged ≥40 years, with a smoking history of ≥10 pack-years, postbronchodilator forced expiratory volume in 1 s (FEV_1_) of ≤70 % of predicted, and FEV_1_ to forced vital capacity (FVC) ratio of <0.70 of predicted. Exclusion criteria included a history of asthma, COPD exacerbation, or respiratory infection within 4 weeks of screening, prior pulmonary resection, and use of supplemental oxygen for >12 h/day. Using centralized randomization by country and by site, patients were randomized to 18 μg tiotropium or placebo (control) (once daily). All respiratory medications, with the exception of other inhaled anticholinergics, were permitted throughout the trial. Postrandomization clinic visits occurred at 1 month, at 3 months, and every 3 months throughout the 4-year treatment period. All patients gave written informed consent and the study was approved by local ethical review boards and conducted in accordance with the Declaration of Helsinki.

### Composite Endpoint of Respiratory Outcomes (CERO) and statistical analysis

As part of the study, safety endpoints were carefully registered; causes of death were assessed by the mortality adjudication committee [16]. The composite outcomes endpoint included fatal events (all-cause mortality); severe ventilatory failure, defined as patients requiring noninvasive or invasive mechanical ventilation and/or hypercapnia as documented in arterial blood gases; severe exacerbations, defined as episodes requiring hospitalization from COPD; and discontinuations from the trial due to worsening COPD. The onset of the first event from the composite endpoint is used to calculate the incidence rate. All patients recruited into the study were included in the analysis for the events of mortality, severe respiratory failure, hospitalizations for COPD, and discontinuation of the study for worsening COPD while on treatment within the study period (within 30 days of last intake of study medication). The analysis of the composite endpoint in this study only included on-treatment fatal events. Although fatal events were recorded beyond the treatment period in UPLIFT®, nonfatal events were not, so that vital status for this period was not included. Incidence rates (IRs) per 100 patient-years and risk ratios (RRs) were determined at 1, 2, 3, and 4 years. Incidence rates were calculated using the ratio of number of patients with the event and time at risk. Under the conditions of an event-driven trial, sample size was calculated assuming exponential survival function as the total number of events needed to achieve a given statistical power for an expected RR (or hazard ratio [HR]) and type I error. Under the conditions of a trial with fixed treatment duration, such as UPLIFT®, number of events was first calculated for a given RR by log-rank test. Number of patients was derived based on the number of events, IR, and treatment duration. For both designs, it was assumed that patients will be followed up for CERO events after discontinuation of the study according to the intent-to-treat principle. Therefore, sample size was not adjusted for possible dropouts. Statistical software nQuery was used to calculate the sample sizes.

## Results

### Baseline characteristics

Altogether, 5992 patients were randomized and received study medication. The baseline characteristics were balanced between treatment groups (Table 1); there were no differences in comorbidity data between the two treatment groups. Mean age was 65 years, 75 % were men, 30 % were current smokers, and mean postbronchodilator FEV_1_ was 1.32 L (47.6 % predicted). The patients had a wide range of airflow limitation and on average had significant impairment in their health status scores.

**Table 1:** Baseline characteristics of the UPLIFT® study population

	Tiotropium (*n* = 2986)	Control (*n* = 3006)	Total (*N* = 5992)
Male, %	75	74	75
Mean (SD) age, y	65 (8)	65 (8)	65 (8)
Current smoker, %	29	30	30
Mean (SD) duration of COPD, y	10 (8)	10 (7)	10 (7)
GOLD stage, %			
II	46	45	46
III	44	44	44
IV	8	9	9
Mean (SD) prebronchodilator			
FEV_1_, L	1.1 (0.4)	1.1 (0.4)	1.1 (0.4)
FEV_1_, % predicted value	40 (12)	39 (12)	39 (12)
FVC, L	2.6 (0.8)	2.6 (0.8)	2.6 (0.8)
FEV_1_/FVC ratio, %	42 (11)	42 (11)	42 (11)
Mean (SD) postbronchodilator			
FEV_1_, L	1.3 (0.4)	1.3 (0.4)	1.3 (0.4)
FEV_1_, % predicted value	48 (13)	47 (13)	48 (13)
FVC, L	3.1 (0.9)	3.1 (0.9)	3.1 (0.9)
FEV_1_/FVC ratio, %	44 (11)	43 (11)	43 (11)
Mean (SD) SGRQ total score, units	46 (17)	46 (17)	46 (17)
Patients with comorbidities, n (%)			
Vascular disorders	1353 (45.3)	1367 (45.5)	2720 (45.4)
Cardiac disorders	790 (26.5)	765 (25.5)	1555 (26.0)
Respiratory, thoracic and mediastinal disorders	565 (18.9)	593 (19.7)	1158 (19.3)

*COPD* chronic obstructive pulmonary disease, *FEV*_*1*_ forced expiratory volume in 1 s, *FVC* forced vital capacity, *GOLD* Global Initiative for Chronic Obstructive Lung Disease, *SD* standard deviation, *SGRQ* St George’s Respiratory Questionnaire

### Composite Endpoint of Respiratory Outcomes (CERO)

**Fig. 1:**
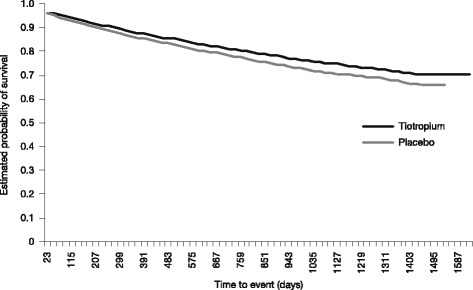
Kaplan–Meier survival event curve for the composite CERO endpoint (tiotropium vs placebo)

The survival event curve (Kaplan–Meier) for the CERO endpoint shows a separation throughout the study in favor of tiotropium (Fig. 1). The IRs and RRs of the CERO endpoints were consistently and significantly lower with tiotropium vs placebo for all 4 years (Table 2). The same was observed for the individual events of respiratory failure, hospitalizations for exacerbations, and discontinuation from the trial due to COPD, but not for death, for which the RRs were significant for years 3 and 4, but not significant for years 1 and 2 (Table 3).

**Table 2:** IR of the CERO endpoint in all patients enrolled in UPLIFT® study on years 1 to 4 of the study

	Tiotropium (*n* = 2986)			Control (*n* = 3006)			Tiotropium/control	
Year	N (%)	Patient -year	IR^a^	N (%)	Patient-year	IR^a^	RR-year	*p*-value
							(95 % CI)	
1	436 (15)	2711.4	16.1	556 (19)	2637.5	21.1	0.76	<0.0001
							(0.67–0.86)	
2	664 (22)	5146.1	12.9	803 (27)	4959.0	16.2	0.80	<0.0001
							(0.72–0.88)	
3	843 (28)	7373.7	11.4	992 (33)	7059.6	14.1	0.81	<0.0001
							(0.74–0.89)	
4	995 (33)	9442.9	10.5	1128 (38)	9007.5	12.5	0.84	0.0001
							(0.77–0.92)	

N = number of events

*CI* confidence interval, *IR* incidence rate, *RR* risk ratio

^a^Per 100 patient-years

**Table 3:** IRs and RRs of the individual events included in the composite COPD endpoint CERO in placebo and tiotropium groups over a 4-year period

	Tiotropium (*n* = 2986)			Control (*n* = 3006)			Tiotropium/control	
Event by year	*N* (%)	Patient-year	IR^a^	*N* (%)	Patient-year	IR^a^	RR-year	*p*-value
							(95 % CI)	
Fatal event								
1	162 (5)	2675.7	6.1	182 (6)	2576.8	7.1	0.86	0.1538
							(0.69-1.06)	
2	238 (8)	5067.4	4.7	261 (9)	4797.4	5.4	0.86	0.1010
							(0.72-1.03)	
3	307 (10)	7222.9	4.3	339 (11)	6741.5	5.0	0.85	0.0328
							(0.72-0.99)	
4	376 (13)	9169.5	4.1	408 (14)	8459.5	4.8	0.85	0.0232
							(0.74-0.98)	
Serious adverse event: Respiratory failure								
1	31 (1)	2726.9	1.1	51 (2)	2626.3	1.9	0.59	0.0187
							(0.37-0.91)	
2	48 (2)	5152.6	0.9	67 (2)	4866.5	1.4	0.68	0.0389
							(0.47-0.98)	
3	68 (2)	7324.4	0.9	88 (3)	6809.8	1.3	0.72	0.0406
							(0.52-0.99)	
4	83 (3)	9267.4	0.9	112 (4)	8513.8	1.3	0.68	0.0079
							(0.51-0.90)	
Serious adverse event: Hospitalization due to exacerbation								
1	272 (9)	2630.9	10.3	326 (11)	2513.9	13.0	0.80	0.0058
							(0.68-0.94)	
2	440 (15)	4825.7	9.1	507 (17)	4515.4	11.2	0.81	0.0014
							(0.71-0.92)	
3	573 (19)	6701.1	8.6	641 (21)	6161.6	10.4	0.82	0.0006
							(0.73-0.92)	
4	681 (23)	8308.8	8.2	735 (25)	7555.3	9.7	0.84	0.0013
							(0.76-0.94)	
Trial discontinuation due to worsening of COPD								
1	92 (3)	2741.3	3.4	178 (6)	2644.4	6.7	0.50	<0.0001
							(0.39-0.64)	
2	146 (5)	5179.9	2.8	250 (8)	4906.4	5.1	0.55	<0.0001
							(0.45-0.68)	
3	202 (7)	7366.6	2.7	317 (11)	6871.0	4.6	0.59	<0.0001
							(0.50-0.71)	
4	237 (8)	9326.7	2.5	367 (12)	8598.9	4.3	0.60	<0.0001
							(0.51-0.70)	

*N* = number of events

*CI* confidence interval, *COPD* chronic obstructive pulmonary disease, *IR* incidence rate, *RR* risk ratio

^a^Per 100 patient-years

The effects of tiotropium versus placebo on the incidence of CERO were significant in most patients, including the 2007 Global Initiative for Chronic Obstructive Lung Disease (GOLD) stage [17] III and IV patients (year 1 − 4) (Table 4).

**Table 4:** IR and RR of the occurrence of CERO in tiotropium and placebo groups for GOLD stage II, and combined GOLD stages III and IV over a 4-year period

GOLD stage II								
	Tiotropium (*n* = 1554)			Control (*n* = 1602)			Tiotropium/control	
Year	*N* (%)	Patient-year	IR^a^	*N* (%)	Patient-year	IR^a^	RR	*p*-value
							(95 % CI)	
1	114 (7)	1312.9	8.7	158 (10)	1255.7	12.6	0.69	0.0025
							(0.54–0.88)	
2	187 (12)	2543.0	7.4	242 (15)	2408.5	10.0	0.73	0.0013
							(0.60–0.89)	
3	249 (16)	3709.7	6.7	312 (20)	3483.4	9.0	0.75	0.0007
							(0.63–0.89)	
4	304 (20)	4818.7	6.3	361 (23)	4501.6	8.0	0.79	0.0021
							(0.68–0.92)	
GOLD stage III/IV (combined)								
	Tiotropium (*n* = 1554)			Control (*n* = 1602)			Tiotropium/control	
Year	*N* (%)	Patient-year	IR^a^	*N* (%)	Patient-year	IR^a^	RR	*p*-value
							(95 % CI)	
1	315 (20)	1354.3	23.3	385 (24)	1340.5	28.7	0.81	0.0055
							(0.70–0.94)	
2	465 (30)	2520.0	18.5	541 (34)	2476.8	21.8	0.84	0.0077
							(0.75–0.96)	
3	579 (37)	3547.6	16.3	659 (41)	3474.6	19.0	0.86	0.0084
							(0.77–0.96)	
4	675 (43)	4475.1	15.1	743 (46)	4378.0	17.0	0.89	0.0266
							(0.80–0.99)	

*N* = number of events

*CI* confidence interval, *GOLD* Global Initiative for Chronic Obstructive Lung Disease, *IR* incidence rate, *RR* risk ratio

^a^Per 100 patient-years

### Calculation of sample size

Using the RRs at 1, 2, 3, and 4 years as the expected HRs in a survival analysis, in a hypothetical event-driven study, the sample size (alpha = 0.05 two-sided, 90 % power) as represented in number of events would be 575, 810, 930, and 1383 for the HRs of 0.76, 0.80, 0.81, and 0.84, respectively, per treatment group (Table 5). Sample sizes were calculated based on the estimated RRs and the IRs provided in Tables 2, 3 and 4. The number of patients to enroll varies depending on the length of the study, including enrollment period. For example, for a HR of 0.76, if the enrollment is 1 year and the maximum length of follow-up is 2 years, then the number of patients would be 1205 per group to achieve 575 events, assuming all patients will be followed up for the composite safety endpoint until study completion. With the same HR, 1-year enrollment, and maximum follow-up of 3 years, the number of patients will reduce to 786 per group to achieve the expected 575 events. The sample sizes with the composite endpoint (GOLD stages II, III, IV, and total group) at 80 % power, 85 % power, and 90 % power showed differences depending on the GOLD stage (Table 5). Sample sizes were calculated based on the estimated RRs and the IRs provided in Tables 2, 3 and 4. Using the IRs and RRs as the expected outcome, the sample size (alpha = 0.05 two-sided, 90 % power) at 1, 2, 3, and 4 years would then be 1546, 1392, 1216, and 1504 per treatment group, respectively.

**Table 5:** Number of events needed to explore the effect of therapy in patients with COPD

	Events				
Power (%)	Year	GOLD II	GOLD III	GOLD IV	Overall
80	1	226	845	436	429
	2	331	1367	638	605
	3	375	1467	1244	695
	4	546	2538	1499	1033
85	1	259	967	499	491
	2	379	1564	730	692
	3	429	1678	1423	795
	4	625	2903	1714	1181
90	1	303	1131	584	575
	2	443	1830	854	810
	3	503	1964	1666	930
	4	731	3397	2006	1383

The calculations were made with a power of 80 %, 85 %, and 90 % at *p* < 0.05

*GOLD* Global Initiative for Chronic Obstructive Lung Disease

## Discussion

This analysis of data obtained in UPLIFT® yielded three important findings. First, a COPD composite endpoint incorporating infrequent serious or significant COPD-related safety outcomes can be useful to demonstrate efficacy and safety in clinical trials. Second, in the UPLIFT® trial, the IRs of CERO events were significantly reduced in patients receiving tiotropium compared with placebo [2]. Third, this approach may help plan studies incorporating efficacy and safety evaluation with a reasonable sample size and shorter time.

Randomized clinical trials represent the gold standard to evaluate the comparative efficacy and safety of novel therapies, especially pharmacological drugs. For highly prevalent and deadly diseases, potential therapies should be evaluated by their impact on important clinical endpoints. An example is COPD, a disease that constitutes one of the top four causes of death in the world and that in its more severe cases results in hospital admissions, which increase personal and societal costs. However, although COPD causes many deaths because of its high prevalence, its long natural course, and the relatively low incidence of deaths or hospitalized exacerbations has resulted in the need to complete studies with very large numbers of patients and over a long period. Thus, the TORCH (Towards a Revolution in COPD Health) and the UPLIFT® studies, which recruited ~6000 patients each, lasted 3 and 4 years, respectively; and the mortality and hospitalization rate signals were relatively weak [1, 2]. Based on those results, two other trials (TIOSPIR® and SUMMIT) evaluating mortality as an outcome needed to recruit more than 16,000 patients each to have sufficient power to address the impact of these interventions on mortality and hospitalized exacerbations [3, 4]. An alternative approach used in other fields with a high burden of disease that may be divided into several outcomes that by themselves are relatively rare has been integrating important clinical outcomes into a composite endpoint that can then represent a more comprehensive measure of the overall response to therapy from a safety perspective. Although most studies have been done in the general field of cardiology [10, 13, 15, 18, 19], there have been many reports of trials using composite endpoints in infectious diseases (including tuberculosis [7]) as well as in stroke [11], diabetes [8], rheumatoid arthritis [20], and renal failure [6], among others. Only one study used a composite endpoint to evaluate the safety of endobronchial valves [13]. Interestingly, some COPD studies used the composite endpoint MACE as a safety composite endpoint, but this was done to evaluate cardiac safety in COPD patients [2, 3, 21–23].

We selected four hard endpoints because of their clinical validity and registered accuracy in this regard; we not only included all-cause mortality (an accepted patient-centered outcome), but also hospitalizations for exacerbations of COPD and respiratory failure. We considered that these latter two outcomes were appropriately associated with all-cause mortality to add strength to the observed results. Similarly, we included discontinuation from the study due to COPD worsening because this event in long clinical trials represents an important signal of decompensation of the target organ and relates to the patients’ own feelings regarding their deteriorating health [24]. We integrated all of the outcomes into the CERO and explored its value in the UPLIFT® study database.

The results of the study indicate a significant effect on the composite index that was of only borderline significance for the individual outcome of mortality in the original report [2]. Indeed, in this regard, neither UPLIFT® nor the TORCH trial has been considered as having shown a significant impact on mortality using mortality including vital status follow-up of discontinued patients [1, 2]. Several authors have criticized the use of composite endpoints when there are contradictions among the results for the individual components and when they are not clearly defined. This is not the case in the current study. As seen in Tables 2 and 3, the tiotropium arm showed a significant impact on the incident rate ratio for CERO compared with placebo, and the RRs for each of the individual components supported the beneficial effect. Further, the results were applicable across a wide range of airflow obstruction (Table 4), supporting general application of the results, at least for patients meeting the criteria for inclusion in UPLIFT®.

Perhaps the most important information provided by the current study is the power calculation of the number of subjects and the duration of the trial needed to obtain a significant result for a therapeutic intervention. Again, using the results from UPLIFT® and the composite measure of CERO, significant reduction in the social and economic cost of completing such trials is theoretically possible.

Using the IRs and RRs as the expected outcome, the sample sizes are certainly much smaller than the SUMMIT trial (16,000 patients) and the completed TIOSPIR® (17,135 patients) study, in which all-cause mortality was the primary endpoint. As the number of medications approved for the treatment of COPD and the benefits that are reported to support their routine use increase, ethical reasons will preclude the completion of long-term trials with a placebo-control arm. Thus, comparison trials may prove to be the way of the future, and the larger signals from composite endpoints a potentially useful tool to evaluate the results of those trials.

We recognize limitations to this study. First, it is a retrospective analysis of data collected for a study in which the primary endpoint was rate of decline of lung function, and the composite endpoint could only be derived from endpoints reported in the study. However, mortality (carefully reviewed by a clinical endpoint committee) was a predetermined endpoint of the study. In addition, respiratory failure and severe exacerbation used in this report were collected as adverse events and the cause for discontinuation of the study carefully recorded. Indeed, we believe careful accounting for the differential dropout from clinical trials represents the strength of this analysis because those patients who discontinue the trial prematurely due to worsening disease [20] are characterized by worse patient-related outcomes. Furthermore, patients dropping out from the placebo group have worse patient-related outcomes than those dropping out from the active treatment group. Second, the elements used to define CERO were chosen after the study was completed. However, these were selected because of their clinical validity, careful recording, and value as reflecting the impact of COPD on the patients themselves. Third, the analysis was an on-treatment analysis based on data collected during the treatment period. The intent-to-treat analysis was not feasible because two components of the CERO, severe respiratory failure and severe exacerbation, were not collected after treatment discontinuation. This likely caused an unfavorable bias against tiotropium because more patients discontinued from placebo than from tiotropium; thus, more events in the placebo arm may otherwise have been recorded had the discontinued patients been followed up. Nevertheless, the exposure was adjusted in the calculation of IR and RR in an attempt to address such bias. If the composite endpoint is used as the primary endpoint in a future study, we recommend that all components are followed up after treatment discontinuation in a true intent-to-treat analysis.

## Conclusions

This study shows that a composite endpoint incorporating infrequent serious or significant COPD-related safety outcomes could be useful in clinical trials. This clinically meaningful approach may help in the design of studies incorporating safety with a feasible sample size; however, further validation of the composite endpoint will be required. In the UPLIFT® trial, the IRs of CERO events were significantly reduced in patients receiving tiotropium.

### Ethics approval and consent to participate

The trial was performed in accordance with the provisions of the Declaration of Helsinki, and the study protocol and procedures were approved by relevant ethics committees at each center. All the patients provided written informed consent.

## Additional file

Additional file 1: (PDF 965 kb)

## Abbreviations

CERO: composite endpoint of respiratory outcomes
COPD: chronic obstructive pulmonary disease
FEV_1_: forced expiratory volume in 1 s
GOLD: global initiative for chronic obstructive lung disease
HR: hazard ratio
IR: incidence rate
MACE: major adverse cardiac events
RR: risk ratio
SUMMIT: study to understand mortality and morbidity in COPD
TIOSPIR®: tiotropium safety and performance in respimat®
TORCH: towards a revolution in COPD health
UPLIFT®: understanding potential long-term impacts on function with tiotropium
