# Supplementary material for: Defining a COPD composite safety endpoint for demonstrating efficacy in clinical trials: results from the randomized, placebo-controlled UPLIFT® trial
Source: Respir Res. 2016 May 4;17:48. doi: 10.1186/s12931-016-0361-4 (PMC4855862; doi:10.1186/s12931-016-0361-4)
Supplement: Additional file 1: — (PDF 965 kb) [file 12931_2016_361_MOESM1_ESM.pdf]

Argentina							
**Centre number(s) / Investigator(s)**	**IRB or IEC (name/address)**	**IRB or IEC Chairperson**	**Informed Consent/Pt. Information Form Approval DD/MM/YY**	**Protocol Approval DD/MM/YY**	**Am. 1 Approval DD/MM/YY**	**Am. 2 Approval DD/MM/YY**	**Am. 3 Approval DD/MM/YY**
101 / Eduardo Schiavi	Comité de Ética "Dr. Virgilio G. Foglia" Tucumán 335 – 7° "D" CP: 1059 – Buenos Aires - Argentina	Dr. Carlos A. Barclay	12/01/06(V 1 Adicional) 19/07/06(V 1 Complementario) 12/08/02 (V 3) 04/02/05 (V 5)	26/04/02	02/07/03	12/01/06	11/05/07
102 / Juan C. Figuerosa Casas	Comité de Ética "Dr. Virgilio G. Foglia" Tucumán 335 – 7° "D" CP: 1059 – Buenos Aires - Argentina	Dr. Carlos A. Barclay	12/01/06(V 1 Adicional) 19/07/06(V 1 Complementario) 12/08/02 (V 3) 04/02/05 (V 5)	26/04/02	02/07/03	12/01/06	11/05/07
103 / Edgardo Rhodius	Comité de Ética "Dr. Virgilio G. Foglia" Tucumán 335 – 7° "D" CP: 1059 – Buenos Aires - Argentina	Dr. Carlos A. Barclay	12/01/06(V 1 Adicional) 19/07/06(V 1 Complementario) 12/08/02 (V 3) 04/02/05 (V 5)	26/04/02	02/07/03	12/01/06	11/05/07
104 / Ricardo Gené	Comité de Ética "Dr. Virgilio G. Foglia" Tucumán 335 – 7° "D" CP: 1059 – Buenos Aires - Argentina	Dr. Carlos A. Barclay	12/01/06(V 1 Adicional) 19/07/06(V 1 Complementario) 12/08/02 (V 3) 04/02/05 (V 5)	26/04/02	02/07/03	12/01/06	11/05/07
105 / César Benito Sáenz	Comité de Ética "Dr. Virgilio G. Foglia" Tucumán 335 – 7° "D" CP: 1059 – Buenos Aires -	Dr. Carlos A. Barclay	12/01/06(V 1 Adicional) 19/07/06(V 1 Complementario) 12/08/02 (V 3) 04/02/05 (V 5)	26/04/02	02/07/03	12/01/06	11/05/07

Argentina							
**Centre number(s) / Investigator(s)**	**IRB or IEC (name/address)**	**IRB or IEC Chairperson**	**Informed Consent/Pt. Information Form Approval DD/MM/YY**	**Protocol Approval DD/MM/YY**	**Am. 1 Approval DD/MM/YY**	**Am. 2 Approval DD/MM/YY**	**Am. 3 Approval DD/MM/YY**
	Argentina						
106 / Eduardo Giugno	Comité de Ética "Dr. Virgilio G. Foglia" Tucumán 335 – 7° "D" CP: 1059 – Buenos Aires - Argentina	Dr. Carlos A. Barclay	12/01/06(V 1 Adicional) 19/07/06(V 1 Complementario) 12/08/02 (V 3) 04/02/05 (V 5)	26/04/02	02/07/03	12/01/06	11/05/07
107 / Carlos Di Bartolo	Comité de Ética "Dr. Virgilio G. Foglia" Tucumán 335 – 7° "D" CP: 1059 – Buenos Aires - Argentina	Dr. Carlos A. Barclay	12/01/06(V 1 Adicional) 19/07/06(V 1 Complementario) 12/08/02 (V 3) 04/02/05 (V 5)	26/04/02	02/07/03	12/01/06	11/05/07
108 / María Cristina De Salvo	Comité de Ética "Dr. Virgilio G. Foglia" Tucumán 335 – 7° "D" CP: 1059 – Buenos Aires - Argentina	Dr. Carlos A. Barclay	12/01/06(V 1 Adicional) 19/07/06(V 1 Complementario) 12/08/02 (V 3) 04/02/05 (V 5)	26/04/02	02/07/03	12/01/06	11/05/07
109 / Eduardo Abbate	Comité de Ética "Dr. Virgilio G. Foglia" Tucumán 335 – 7° "D" CP: 1059 – Buenos Aires - Argentina	Dr. Carlos A. Barclay	12/01/06(V 1 Adicional) 19/07/06(V 1 Complementario) 12/08/02 (V 3) 04/02/05 (V 5)	26/04/02	02/07/03	12/01/06	11/05/07
110 / Ana María López	Comité de Ética "Dr. Virgilio G. Foglia" Tucumán	Dr. Carlos A. Barclay	12/01/06(V 1 Adicional) 19/07/06(V 1 Complementario)12/08/02	26/04/02	02/07/03	12/01/06	11/05/07

Argentina							
**Centre number(s) / Investigator(s)**	**IRB or IEC (name/address)**	**IRB or IEC Chairperson**	**Informed Consent/Pt. Information Form Approval DD/MM/YY**	**Protocol Approval DD/MM/YY**	**Am. 1 Approval DD/MM/YY**	**Am. 2 Approval DD/MM/YY**	**Am. 3 Approval DD/MM/YY**
	335 – 7° "D" CP: 1059 – Buenos Aires - Argentina		(V 3) 04/02/05 (V 5)				

Australia							
**Centre number(s) / Investigator(s)**	**IRB or IEC (name/address)**	**IRB or IEC Chairperson**	**Informed Consent/Pt. Information Form Approval DD/MM/YY**	**Protocol Approval DD/MM/YY**	**Am. 1 Approval DD/MM/YY**	**Am. 2 Approval DD/MM/YY**	**Am. 3 Approval DD/MM/YY**
201 / Chris Steinfort	St John Of God Health Care Ethics Committee	Professor Con Michael	06Jun2002	06Jun2002	02Oct2003	02Feb2006	07Jun2007
202 / Matthew Peters	Central Sydney Area Health Service HREC –CRGH Zone	Dr Garry Pearce	25Jul2002	25Jul2002	19Aug2003	21Mar2006	19Jun2007
203 / Patrick Carroll	Redcliffe-Caboolture Health Service District Ethics Commmittee	Mark Zgrajewski	05Jun2002	05Jun2002	06Aug2003	01Feb2006	06Jun2007
204 / Graham Simpson	Cairns Base Hospital Ethics Committee	Marlane Byrne	04Jul2002	04Jul2002	31Jul2003	23Mar2006	21Jun2007
205 / David Freiberg	South Western Sydney Area Health Service HREC	Prof Hugh Dickson	25Jul2002	25Jul2002	23Jul2003	22Dec2005	06Jun2007
206 / Paul Fogarty	Eastern Health Research and Ethics Committee	Lee Hamley	25Jul2002	25Jul2002	21Aug2003	18Jan2006	19Jul07
207 / Richard Watts	ACHA Research & Ethics Committee/ Bellberry HREC	Dr Brian Stoffell	25Mar2003	20Nov2002	15Oct2003	18Jan2006	20Jun2007
209 / John Wheatley	Western Sydney Area Health Service Human research Ethics Committee	Prof Stephen Leader	17Oct2002	17Oct2002	28Jul2003	03Feb2006	29May2007

Austria							
**Centre number(s) / Investigator(s)**	**IRB or IEC (name/address)**	**IRB or IEC Chairperson**	**Informed Consent/Pt. Information Form Approval DD/MM/YY**	**Protocol Approval DD/MM/YY**	**Am. 1 Approval DD/MM/YY**	**Am. 2 Approval DD/MM/YY**	**Am. 3 Approval DD/MM/YY**
301 / Norbert Vetter	Ethikkommission der Stadt Wien<br>Thomas Klestil Platz 8<br>A-1130 Wien,<br>EK.Nr.: 02-057-0602	Frau<br>Dr. Karin Spacek	14.Nov.02 (Vers. II)<br>01.Dec.04 (Vers. III)<br>27.Dec.05 (Long Term)<br>30.Aug.06 (Add. Info.)	14.Nov.02	Not submitted in Austria	27.Dec.05	15. Jun.07
302 / Otto Burghuber	Ethikkommission der Stadt Wien<br>Thomas Klestil Platz 8<br>A-1130 Wien,<br>EK.Nr.: 02-057-0602	Frau<br>Dr. Karin Spacek	14.Nov.02 (Vers. II)<br>01.Dec.04 (Vers. III)<br>27.Dec.05 (Long Term)<br>30.Aug.06 (Add. Info.)	14.Nov.02	Not submitted in Austria	27.Dec.05	15.Jun.07
303 / Dr. Christian Hesse	Ethikkommission der medizinischen Universität Graz<br>Auenbruggerplatz 29<br>A-8036 Graz<br>EK.Nr.: 12-177 ex 01/02	Herr Univ. Prof. Dr. Peter Rehak	31.Oct.02 (Vers. II)<br>15.Jul.04 (Vers. III)<br>12.Jan.06 (Vers. IV)<br>17.Jan.06 (Long Term)<br>11.Oct.06 (Add. Info)	31.Oct.02	Not submitted in Austria	17.Jan.06	11.Jun.07
304 / Martin Flicker	Ethikkommission der medizinischen Universität Graz<br>Auenbruggerplatz 29<br>A-8036 Graz<br>EK.Nr.: 12-189 ex	Herr Univ. Prof. Dr. Peter Rehak	31.Oct.02 (Vers. II)<br>12.Jan.06 (Vers. III)<br>17.Jan.06 (Long Term)	31.Oct.02	Not submitted in Austria	17.Jan.06	11.Jun.07

Austria							
**Centre number(s) / Investigator(s)**	**IRB or IEC (name/address)**	**IRB or IEC Chairperson**	**Informed Consent/Pt. Information Form Approval DD/MM/YY**	**Protocol Approval DD/MM/YY**	**Am. 1 Approval DD/MM/YY**	**Am. 2 Approval DD/MM/YY**	**Am. 3 Approval DD/MM/YY**
	01/02		11.Oct.06 (Add. Info)				
305 / Christian Kähler	Ethikkommission der medizinischen Universität Innsbruck Innrain 43 A-6020 Innsbruck EK.Nr.: UN 1626 197/4.14	Herr Univ. Prof. Dipl. Ing. Dr. Peter Lukas	30.Aug.02 (Ver. II) 17.Dec.04 (Vers. III) 27.Jan.06 (Long Term) 20.Oct.06 (Add. Info)	30.Aug.02	Not submitted in Austria	27.Jan.06	01.Jun.07

Belgium							
**Centre number(s) / Investigator(s)**	**IRB or IEC (name/address)**	**IRB or IEC Chairperson**	**Informed Consent/Pt. Information Form Approval DD/MM/YY**	**Protocol Approval DD/MM/YY**	**Am. 1 Approval DD/MM/YY**	**Am. 2 Approval DD/MM/YY**	**Am. 3 Approval DD/MM/YY**
402 / Greet Bral	Etische Commissie Jan Yperman Ziekenhuis Briekestraat, 12 B – Yeper 8900	Mr. J.Vandenbulcke	03/06/2002	20/11/2002	Not Applicable	20/12/2007	06/12/2007
403 / Kris Carron	Medische Etische Commissie Heilig HartZiekenhuius Wilgenstraat, 2 B – 8800 Roeselare	Dr. L. Marcelis	03/06/2002	22/10/2002	15/09/2003	23/01/2006	20/08/2007
404 / D. Coolen	Etische Commissie Campus Drie Eicken D T 506 Universiteitsplein, 1 B – 2610 Wilrijk	Prof. P. De Deyn	03/06/2002	03/07/2002	15/11/2003	08/02/2006	Not available
405 / Wilfried De Backer	Etische Commissie UZA Wilrijkstraat, 10 B – 2650 Edegem	Prof. P. Cras	03/06/2002	19/08/2002	18/07/2003	07/02/2006	24/05/2007
406 / Tom De Beukelaar	Etische Commissie Campus Drie Eicken D T 506 Universiteitsplein, 1 B – 2610 Wilrijk	Prof. P. De Deyn	03/06/2002	04/09/2002	05/11/2003	08/02/2006	11/07/2007
407 / M. Decramer	Commissie Medische Ethiek van de UZ KU Leuven - Gasthuisberg Herestraat, 49 B – 3000 Leuven	Prof. J. Vermylen	03/06/2002	19/06/2002	08/07/2003	11/01/2006	03/12/2007

Belgium							
**Centre number(s) / Investigator(s)**	**IRB or IEC (name/address)**	**IRB or IEC Chairperson**	**Informed Consent/Pt. Information Form Approval DD/MM/YY**	**Protocol Approval DD/MM/YY**	**Am. 1 Approval DD/MM/YY**	**Am. 2 Approval DD/MM/YY**	**Am. 3 Approval DD/MM/YY**
409 / Rene Deman	Ethische Commissie AZ Groeninge Campus Saint Maarten Loofstraat, 43 B – 8500 Kortrijk	Dr. L. Van Lysebeth	03/06/2002	10/09/2002	07/10/2003	27/11/2006	04/12/2007
412 / Bruno Dewispelaere	Medische Ethische Commissie AZ Sint- Josef Steenweg op Merksplas, 44 B – 2300 Turnhout	Dr. B. Reynders	03/06/2002	22/08/2002	01/10/2003	11/12/2006	01/10/2007
413 / P. Driesen	Etische Comité Saint Elisabeth Ziekenhuis Rubenstraat, 166 B – 2300 Turnhout	Mr. K. Ackaert	03/06/2002	26/06/2002	16/06/2003	31/01/2006	18/05/2007
414 / Willy Elinck	Commissie voor Medische Ethiek Jan Palfijn Henridunantlaan, 5 B – 9000 Gent	Dr. L. Van Nimmen	03/06/2002	09/09/2002	Not Applicable	13/03/2006	29/10/2007
417 / Yves Mentens	Ethische Commissie AZ Sint-Elisabeth Nederrij, 133 B – 2200 Herentals	Dr. M. Vansteenkiste	03/06/2002	17/09/2002	16/09/2003	06/12/2006	30/05/2007
418 / Guy Joos	Etische Commissie UZ Gent De Pintelaan, 185 B – 9000 Gent	Prof. M. Bogaert	03/06/2002	05/09/2002	Not Applicable	Not Applicable	Not Applicable
420 / Kurt Vandeurzen	Commissie voor Medische Ethiek Maesenveld, 1	Dr. F. Ulens	03/06/2002	18/09/2002	10/09/2003	15/03/2006	21/05/2007

Belgium							
**Centre number(s) / Investigator(s)**	**IRB or IEC (name/address)**	**IRB or IEC Chairperson**	**Informed Consent/Pt. Information Form Approval DD/MM/YY**	**Protocol Approval DD/MM/YY**	**Am. 1 Approval DD/MM/YY**	**Am. 2 Approval DD/MM/YY**	**Am. 3 Approval DD/MM/YY**
	B – 3900 Overpelt						
421 / Dirk Van Renterghem	Etische Commissie AZ Sint Jan Ruddershove, 10 B – 8000 Brugge	Dr. Van Droogenbroek	03/06/2002	14/08/2002	26/06/2003	26/01/2006	21/11/2007
422 / Luc Van Zandweghe	Etische Commissie AZ Sint Blasius Kroonveldlaan, 50 B – 9200 Dendermonde	Dr. B. D' Haen	03/06/2002	18/09/2002	13/11/2003	27/03/2006	27/07/2007
423 / Wim Verhaeghe	Etische Commissie Ziekenhuis H. Serruys Kaïrostraat, 84 B – 8400 Oostende	Dr. G. Pulinckx	03/06/2002	20/09/2002	19/11/2003	03/02/2006	18/05/2007
424 / W. Vincken	Commissie Medische Ethiek UZ Brussel Laarbeeklaan, 101 B – 1090 Brussels	Prof. P. Devroey	03/06/2002	01/08/2002	04/07/2003	09/01/2006	14/07/2007
428 / Luc Delaunois	Comité d'Ethique Hospitalier Clinique Universitaire de Mont-Godinne Ave. Docteur Gaston Ghérasse, 1 B – 5530 Yvoir	Dr. A. Evrard	03/06/2002	03/10/2002	16/07/2003	01/02/2006	23/05/2007

Belgium							
**Centre number(s) / Investigator(s)**	**IRB or IEC (name/address)**	**IRB or IEC Chairperson**	**Informed Consent/Pt. Information Form Approval DD/MM/YY**	**Protocol Approval DD/MM/YY**	**Am. 1 Approval DD/MM/YY**	**Am. 2 Approval DD/MM/YY**	**Am. 3 Approval DD/MM/YY**
429 / Duchatelet	Comité d'Ethique RHMS<br>Clinique Louis Cathy<br>Rue Louis Cathy, 136<br>B – 7331 Baudour	Dr. de Brouckère	03/06/2002	15/10/2002	01/10/2003	23/02/2006	Not available
432 / André Noseda	Comité d'Ethique Hospitalier Brugman<br>Place A. Van Gehuchten, 4<br>B – 1020 Brussels	Dr. J. Valsamis	03/06/2002	10/10/2002	18/09/2003	14/03/2006	10/07/2007
433 / Guy Nuttin	Commission d'Ethique CHR de Tournai<br>Boulevard Lalaing, 39<br>B – 7500 Tournai	Dr. Desplanque	03/06/2002	30/01/2003	18/08/2003	22/12/2005	11/05/2007
434 / Daniel Rodenstein	Commission d'Ethique Biomédicale<br>Hospitalo Facultaire<br>Ave Hippocrate, 55<br>Tour Harvey, niveau 0<br>B – 1200 Brussels	Dr. JM Maloteaux	03/06/2002	09/10/2002	03/11/2004	15/11/2006	18/05/2007
435 / Annick Thibaut-Baudrez	CRP Santé Comité National d'Ethique<br>Hôpital Princesse Marie Astrid<br>Rue Thomas Edison, 1AB<br>B – 1210 Luxembourg	Dr. Michel	03/06/2002	01/10/2002	31/05/2007	31/05/2007	Not available

Belgium							
**Centre number(s) / Investigator(s)**	**IRB or IEC (name/address)**	**IRB or IEC Chairperson**	**Informed Consent/Pt. Information Form Approval DD/MM/YY**	**Protocol Approval DD/MM/YY**	**Am. 1 Approval DD/MM/YY**	**Am. 2 Approval DD/MM/YY**	**Am. 3 Approval DD/MM/YY**
437 / Guy Vandermoten	Commission d'Ethique Centre Hospitalier Régional de Namur<br>Ave Albert 1er, 185<br>B – 5000 Namur	Dr. A. Fox	03/06/2002	17/09/2002	Not available	25/04/2006	22/05/2007
438 / Patricia Wackeniers-Genard	Commission d' Ethique CHU Ambroise Paré Commission d'Ethique<br>Boulevard Kennedy, 2<br>B – 7000 Mons	Dr. D. Diana	03/06/2002	26/06/2002	24/09/2003	19/04/2006	13/09/2007
439 / Sandra Baldassarre	Commission d'Ethique<br>H.U. Erasme<br>Route de Lennik, 808<br>B - 1070 Brussels	Prof. A. Herchuelz	03/06/2002	24/09/2002	26/08/2003	24/01/2006	02/10/2007
440 / Schlesser	CRP Santé Comité National d'Ethique Centre Hospitalier de Luxembourg<br>Rue Thomas Edison, 1AB<br>L – 1417 Luxembourg	Dr. G. Michel	03/06/2002	06/11/2002	21/10/2004	02/05/2007	Not available

Brazil							
**Centre number(s) / Investigator(s)**	**IRB or IEC (name/address)**	**IRB or IEC Chairperson**	**Informed Consent/Pt. Information Form Approval DD/MM/YY**	**Protocol Approval DD/MM/YY**	**Am. 1 Approval DD/MM/YY**	**Am. 2 Approval DD/MM/YY**	**Am. 3 Approval DD/MM/YY**
501 / Sérgio Menna Barreto	Comissão de Pesquisa e Ética em Saúde do Hospital de Clínicas de Porto Alegre	Sérgio Pinto Machado	v1 27Mar02- 13May02; v2 09Mar05- 08Apr05; v3 15May06- 10Aug06; vital ICF - missing	13May02	N/A	20Feb06	N/A
502 / Joao Carlos Corrêa	Comitê de Ética em Pesquisa em Seres Humanos	Sylvio Carlos Corrêa	v1 27Mar02- 07may02; v2 09Mar05- 05Apr05; v3 15May06- 03aug06; vital ICF- 18Jan06	07May02	N/A	18Jan06	N/A
503 / Luiz Carlos Corrêa da Silva	Comitê de Ética em Pesquisa da Irmandade da Santa Casa de Misericórdia de Porto Alegre	Claudio Teloken	v1 27Mar02- 07May02; v1.1 24Sep02- 29Oct02; v2 09Nov04- 05Oct05; v3 25Jul06- 29Aug06; vital ICF- 29Aug06	07May02	N/A	29Aug06	N/A
504 / Elie Fiss	Comitê de Ética em Pesquisa da Faculdade de Medicina do ABC	Márcia Tamosauskas	v1 27Mar02- 16May02; v2 09Mar05- 11May05; v3 25Jul06- 22Aug06; vital ICF- 13Mar06	16May02	N/A	13Mar06	N/A
505 / Antônio Chibante	Comitê de Ética em Pesquisa	Fernanda Medeiros	v1 27Mar02- 20May02; v2 09Mar05- 09Dec05; v3 09Mar06- missing; v4 25Jul06- 21Aug06; vital ICF- 06Jul06	20May02	N/A	06Jul06	N/A

Brazil							
**Centre number(s) / Investigator(s)**	**IRB or IEC (name/address)**	**IRB or IEC Chairperson**	**Informed Consent/Pt. Information Form Approval DD/MM/YY**	**Protocol Approval DD/MM/YY**	**Am. 1 Approval DD/MM/YY**	**Am. 2 Approval DD/MM/YY**	**Am. 3 Approval DD/MM/YY**
506 / Waldo Mattos	Comitê de Ética em Pesquisa do Grupo Hospitalar Conceição	Julio Baldissertotto	v1 27mar02-12Jun02; v2 09Mar05- 11Aug05; v3 25Jul06-18Oct06; vital ICF-16May06	12Jun02	N/A	16May06	N/A
507 / Carlos Fritscher	Comitê de Ética em Pesquisa da PUCRS	José Roberto Goldim	v1 27Mar02-14May02; v2 09Mar05- 05Aug05; v3 15May06-24Aug06; vital ICF-20Mar06	14May02	N/A	20Mar06	15Jan08
508 / Julio Abreu de Oliveira	Comitê de Ética em Pesquisa da Universidade Federal de Juiz de Fora	Sonia Maria Dias	v1 27Mar02-15May02<br>v2 09Mar05-19May05<br>v3 15May06-17Aug06<br>vital ICF- 16Feb06	15May02	18Dec03	16Feb06	N/A
510 / Dr. Emilio Pizzichini	Comitê de Ética em Pesquisa com Seres Humanos da Universidade Federal de Santa Catarina	Washington Portela de Souza	v1 27Mar02-27May02<br>v2 09Mar05-25Apr05<br>v3 15May06-28Aug06<br>vital ICF- 06Mar06	27May02	N/A	06Mar06	N/A
511 / Alvaro Cruz	Comitê de Ética em Pesquisa - CEP / MCO / UFBA	Antônio dos Santos Barata	v1 27Mar02-05Jun02<br>v2 09Mar05-30May05<br>v3 15May06-08Dec06	05Jun02	03Dec03	29Apr06	21Jan08

Brazil							
**Centre number(s) / Investigator(s)**	**IRB or IEC (name/address)**	**IRB or IEC Chairperson**	**Informed Consent/Pt. Information Form Approval DD/MM/YY**	**Protocol Approval DD/MM/YY**	**Am. 1 Approval DD/MM/YY**	**Am. 2 Approval DD/MM/YY**	**Am. 3 Approval DD/MM/YY**
			vital ICF- 29Apr06				
512 / Carlos a. de Castro Pereira	Comitê de Ética em Pesquisa do IAMSPE/HSPE - "F.M.O."	Ana Maria Moraes deAndrade	v1 27Mar02- 15Jul02<br>v2 09Mar05- 26Apr05<br>v3 15May06- 29Aug06<br>vital ICF- 21Feb06	15Jul02	13Feb04	21Feb06	N/A
513 / Alberto Cukier	Comissão de Ética para Análise de Projetos de Pesquisa - CAPPesq	Euclides Ayres de Castilho	v1 27Mar02- 25Jul02<br>v2 09Mar05- 22Jun05<br>v3 15May06- 13Sep06<br>vital ICF- 14Jun06	25Jul02	18Feb04	14Jun06	N/A
514 / Roberto Stirbulov	Comitê de Ética em Pesquisa em Seres Humanos da Santa Casa de Misericórdia de São Paulo	Daniel Romero Muñoz	v1 27Mar02- 19Aug02<br>v2 09Mar05- 14Oct05<br>v3 15May06- 21Dec06<br>vital ICF- 02Jun06	19Aug02	27Feb04	21May06	N/A
515 / Jose Roberto Brito Jardim	Comitê de Ética em Pesquisa da universidade Federal de São Paulo / Hospital São Paulo	José Osmar Medina Pestana	v1 27Mar02- 20Sep02<br>v2 09Mar05- 01Dec05<br>v3 15May06- 29Sep06<br>vital ICF- 13Feb06	20Sep02	N/A	13Feb06	N/A

Czechia							
**Centre number(s) / Investigator(s)**	**IRB or IEC (name/address)**	**IRB or IEC Chairperson**	**Informed Consent/Pt. Information Form Approval DD/MM/YY**	**Protocol Approval DD/MM/YY**	**Am. 1 Approval DD/MM/YY**	**Am. 2 Approval DD/MM/YY**	**Am. 3 Approval DD/MM/YY**
701 / Kveta Kalandrova	Etická komise Fakultní nemocnice a Lékařské fakulty UK Plzeň tř. Dr. E. Beneše 13 305 99 Plzeň	Dr. Jindřich Fínek	20.6.2002	20.6.2002	11.9.2003	9.2.2006	16.8.2007
702 / Marie Novakova	Etická komise Fakultní nemocnice a Lékařské fakulty UK Plzeň tř. Dr. E. Beneše 13 305 99 Plzeň	Dr. Jindřich Fínek	20.6.2002	20.6.2002	11.9.2003	9.2.2006	16.8.2007
703 / Petr Hartman	Etická komise Fakultní nemocnice a Lékařské fakulty UK Plzeň tř. Dr. E. Beneše 13 305 99 Plzeň	Dr. Jindřich Fínek	20.6.2002	20.6.2002	11.9.2003	9.2.2006	16.8.2007
704 / Viktor Jenovsky	Etická komise Plicní a interní ordinace Terazínská - 487 410 02, Lovosice	Dr. Roman Záhora	17.9.2002	17.9.2002	1.10.2003	2.3.2006	
705 / Jan Vacek	Etická komise Nemocnice Jablonec nad Nisou Nemocniční 15 466 01 Jablonec nad Nisou	Dr. Petr Žák	30.10.2002	30.10.2002	9.9.2003	20.3.2006	24.10.2007
706 / Viktor Kasak	Etická komise při Institutu klin. a exp. Medicíny	Dr. Vladimír Staněk	12.6.2002	12.6.2002	8.10.2003	8.2.2006	11.7.2007

Czechia							
**Centre number(s) / Investigator(s)**	**IRB or IEC (name/address)**	**IRB or IEC Chairperson**	**Informed Consent/Pt. Information Form Approval DD/MM/YY**	**Protocol Approval DD/MM/YY**	**Am. 1 Approval DD/MM/YY**	**Am. 2 Approval DD/MM/YY**	**Am. 3 Approval DD/MM/YY**
	Thomayerově FN<br>Vídeňská - 800<br>140 59 Praha - 4						
707 / Jiri Erban	Etická komise SPLIN s.r.o.<br>Nestátní zdravotnické zařízení<br>Čimická - 37<br>182 00 Praha - 8	Dr. Oldřich Koberec	9.10.2002	9.10.2002	29.7.2003	20.3.2006	8.8.2007
708 / Pavel Kalina	Etická komise SPLIN s.r.o.<br>Nestátní zdravotnické zařízení<br>Čimická - 37<br>182 00 Praha - 8	Dr. Oldřich Koberec	9.10.2002	9.10.2002	29.7.2003	20.3.2006	8.8.2007
709 / Jan Chlumsky	Etická komise<br>při Institutu klin. a exp. Medicíny<br>Thomayerově FN<br>Vídeňská - 800<br>140 59 Praha - 4	Dr. Vladimír Staněk	12.6.2002	12.6.2002	8.10.2003	8.2.2006	11.7.2007
710 / Jaromir Musil	Etická komise FN Motol<br>V Úvalu - 84<br>150 06 Praha - 5	Dr. Vratislav Šmelhaus	28.8.2002	28.8.2002	27.11.2003	18.1.2006	6.6.2007
711 / Ivo Hojka	Etická komise FN Motol<br>V Úvalu - 84<br>150 06 Praha - 5	Dr. Vratislav Šmelhaus	17.9.2002	17.9.2002	5.8.2003	31.1.2006	6.6.2007
712 / Alena Havlikova	Etická komise<br>Nem. Rudolfa a Stefanie	Dr. Jiří Bráza	17.9.2002	17.9.2002	5.3.2003	20.2.2006	23.8.2007

Czechia							
**Centre number(s) / Investigator(s)**	**IRB or IEC (name/address)**	**IRB or IEC Chairperson**	**Informed Consent/Pt. Information Form Approval DD/MM/YY**	**Protocol Approval DD/MM/YY**	**Am. 1 Approval DD/MM/YY**	**Am. 2 Approval DD/MM/YY**	**Am. 3 Approval DD/MM/YY**
	Máchova - 400<br>256 30, Benešov u Prahy						
713 / Jaroslav Mares	Etická komise Nemocnice Strakonice<br>Radomyšlská - 336<br>386 29, Strakonice	Dr. Pavel Švihálek	11.6.2002	11.6.2002	2.12.2003	7.3.2006	14.8.2007
714 / Kamil Klenha	Etická komise Okresní Nemocnice Tábor<br>Kpt. Jaroše - 2000<br>390 19, Tábor	Dr. Kamil Kleňha	20.6.2002	20.6.2002	26.6.2003	7.2.2006	12.9.2007
715 / Vladimir Vondra	Etická komise SPLIN s.r.o.<br>Nestátní zdravotnické zařízení<br>Čimická - 37<br>182 00 Praha - 8	Dr. Oldřich Koberec	9.10.2002	9.10.2002	29.7.2003	20.3.2006	8.8.2007
716 / Milos Havlik	Etická komise ECOLAB<br>Václavské Náměstí - 1<br>669 02 Znojmo	Dr. Hulán Karel	16.10.2002	16.10.2002	10.8.2003	27.3.2006	25.6.2007
717 / Zdenka Parakova	Etická komise<br>FN Hradec Králové<br>Sokolská 408<br>500 05 Hradec Králové	Dr. Jiří Vortel	14.11.2002	14.11.2002	10.10.2003	9.2.2006	2.7.2007
718 / Jiri Vlcek	Etická komise FN u sv. Anny v Brně<br>Pekařská 53	Dr. Vladimir Soška	24.6.2002	24.6.2002	22.8.2003	21.4.2006	14.6.2007

Czechia							
**Centre number(s) / Investigator(s)**	**IRB or IEC (name/address)**	**IRB or IEC Chairperson**	**Informed Consent/Pt. Information Form Approval DD/MM/YY**	**Protocol Approval DD/MM/YY**	**Am. 1 Approval DD/MM/YY**	**Am. 2 Approval DD/MM/YY**	**Am. 3 Approval DD/MM/YY**
	656 91 Brno						
719 / Jana Dvorakova	Etická komise Čs. Armády 1076 562 18 Ústí nad Orlicí	Dr.Miroslav Kareš	8.7.2002	8.7.2002	25.8.2003	1.2.2006	6.6.2007
720 / Helena Mazacova	Etická komise Nemocnice Polesí Konská 453 739 61 Třinec	Dr. Marian Branny	4.11.2002	4.11.2002	25.8.2003	1.3.2006	27.9.2007
721 / Velart Dusan	Etická komise při M.I.O. s.r.o. K nemocnici 32 741 01 Nový Jičín	Dr.Jaromir Gumulec	6.10.2002	6.10.2002	10.9.2003	6.4.2006	13.9.2007
722 / Ladislav Pospisil	Etická komise Havlíčkova 660 767 55 Kroměříž	Dr.Lumír Domes	13.6.2002	13.6.2002	12.9.2003	17.3.2006	12.7.2007
723 / Dr. Olga Zajicova	Etická komise FN a LF Olomouc I.P. Pavlova 6 775 20 Olomouc	Dr.Vladko Horčička	17.6.2002	17.6.2002	18.8.2003	20.2.2006	18.6.2007
724 / Petr Kolman	Etická komise Nemocnice Kyjov Strážovská 976 697 33 Kyjov	Dr.Luděk Šimeček	11.11.2002	11.11.2002	25.8.2003	21.3.2006	8.8.2007
725 / Ilona Binková	Etická komise FN Brno Bohunice Jihlavská 20 625 00 Brno	Dr.Šárka Sedláčková	27.11.2002	27.11.2002	11.9.2003	8.2.2006	20.6.2007

Czechia							
**Centre number(s) / Investigator(s)**	**IRB or IEC (name/address)**	**IRB or IEC Chairperson**	**Informed Consent/Pt. Information Form Approval DD/MM/YY**	**Protocol Approval DD/MM/YY**	**Am. 1 Approval DD/MM/YY**	**Am. 2 Approval DD/MM/YY**	**Am. 3 Approval DD/MM/YY**
726 / Josef Fratrik	Etická komise<br>FN Hradec Králové<br>Sokolská 408<br>500 05 Hradec Králové	Dr. Jiří Vortel	14.11.2002	14.11.2002	10.10.2003	9.2.2006	2.7.2007

Denmark							
**Centre number(s) / Investigator(s)**	**IRB or IEC (name/address)**	**IRB or IEC Chairperson**	**Informed Consent/Pt. Information Form Approval DD/MM/YY**	**Protocol Approval DD/MM/YY**	**Am. 1 Approval DD/MM/YY**	**Am. 2 Approval DD/MM/YY**	**Am. 3 Approval DD/MM/YY**
801 / Peter Lange	Current address:<br>De Videnskabsetiske Komitéer for Region Hovedstaden<br>Regionsgaarden<br>Kongens Vaenge 2<br>DK-3400 Hilleroed<br><br>Address on approval:<br>Sjaellandsgade 40<br>DK-2200 Koebenhavn N	Jens Bülow	25-10-02	11-07-02	12-08-03	21-07-07 (Approval from the Danish Health Authority)	08-06-07
802 / Vibeke Backer	Current address:<br>De Videnskabsetiske Komitéer for Region Hovedstaden<br>Regionsgaarden<br>Kongens Vaenge 2<br>DK-3400 Hilleroed<br><br>Address on approval:<br>Sjaellandsgade 40<br>DK-2200 Koebenhavn N	Jens Bülow	25-10-02	11-07-02	12-08-03	21-07-07 (Approval from the Danish Health Authority)	08-06-07
803 / Ronald Dahl	Current address:<br>De Videnskabsetiske Komitéer for Region Hovedstaden<br>Regionsgaarden<br>Kongens Vaenge 2<br>DK-3400 Hilleroed	Jens Bülow	25-10-02	11-07-02	12-08-03	21-07-07 (Approval from the Danish Health Authority)	08-06-07

Denmark							
**Centre number(s) / Investigator(s)**	**IRB or IEC (name/address)**	**IRB or IEC Chairperson**	**Informed Consent/Pt. Information Form Approval DD/MM/YY**	**Protocol Approval DD/MM/YY**	**Am. 1 Approval DD/MM/YY**	**Am. 2 Approval DD/MM/YY**	**Am. 3 Approval DD/MM/YY**
	Address on approval:<br>Sjaellandsgade 40<br>DK-2200<br>Koebenhavn N						
804 / Henrik Harving	Current address:<br>De Videnskabsetiske Komitéer for Region Hovedstaden<br>Regionsgaarden<br>Kongens Vaenge 2<br>DK-3400 Hilleroed<br><br>Address on approval:<br>Sjaellandsgade 40<br>DK-2200<br>Koebenhavn N	Jens Bülow	25-10-02	11-07-02	12-08-03	21-07-07 (Approval from the Danish Health Authority)	08-06-07
805 / Phillip TØnnesen	Current address:<br>De Videnskabsetiske Komitéer for Region Hovedstaden<br>Regionsgaarden<br>Kongens Vaenge 2<br>DK-3400 Hilleroed<br><br>Address on approval:<br>Sjaellandsgade 40<br>DK-2200<br>Koebenhavn N	Jens Bülow	25-10-02	11-07-02	12-08-03	21-07-07 (Approval from the Danish Health Authority)	08-06-07
806 / Erik Munch	Current address:<br>De Videnskabsetiske Komitéer for Region Hovedstaden	Jens Bülow	25-10-02	11-07-02	12-08-03	21-07-07 (Approval from the Danish	08-06-07

Denmark							
**Centre number(s) / Investigator(s)**	**IRB or IEC (name/address)**	**IRB or IEC Chairperson**	**Informed Consent/Pt. Information Form Approval DD/MM/YY**	**Protocol Approval DD/MM/YY**	**Am. 1 Approval DD/MM/YY**	**Am. 2 Approval DD/MM/YY**	**Am. 3 Approval DD/MM/YY**
	Regionsgaarden<br>Kongens Vaenge 2<br>DK-3400 Hilleroed<br>Address on approval:<br>Sjaellandsgade 40<br>DK-2200<br>Koebenhavn N					Health Authority)	
807 / Ole Bruun Rasmussen	Current address:<br>De Videnskabsetiske Komitéer for Region Hovedstaden<br>Regionsgaarden<br>Kongens Vaenge 2<br>DK-3400 Hilleroed<br><br>Address on approval:<br>Sjaellandsgade 40<br>DK-2200<br>Koebenhavn N	Jens Bülow	25-10-02	11-07-02	12-08-03	21-07-07 (Approval from the Danish Health Authority)	08-06-07
808 / Flemming Egede	Current address:<br>De Videnskabsetiske Komitéer for Region Hovedstaden<br>Regionsgaarden<br>Kongens Vaenge 2<br>DK-3400 Hilleroed<br><br>Address on approval:<br>Sjaellandsgade 40<br>DK-2200<br>Koebenhavn N	Jens Bülow	25-10-02	11-07-02	12-08-03	21-07-07 (Approval from the Danish Health Authority)	08-06-07

Denmark							
**Centre number(s) / Investigator(s)**	**IRB or IEC (name/address)**	**IRB or IEC Chairperson**	**Informed Consent/Pt. Information Form Approval DD/MM/YY**	**Protocol Approval DD/MM/YY**	**Am. 1 Approval DD/MM/YY**	**Am. 2 Approval DD/MM/YY**	**Am. 3 Approval DD/MM/YY**
809 / Niels Christian Hansen	Current address:<br>De Videnskabsetiske Komitéer for Region Hovedstaden Regionsgaarden Kongens Vaenge 2 DK-3400 Hilleroed<br><br>Address on approval:<br>Sjaellandsgade 40 DK-2200 Koebenhavn N	Jens Bülow	25-10-02	11-07-02	12-08-03	21-07-07 (Approval from the Danish Health Authority)	08-06-07
810 / AsbjØrn HØegholm	Current address:<br>De Videnskabsetiske Komitéer for Region Hovedstaden Regionsgaarden Kongens Vaenge 2 DK-3400 Hilleroed<br><br>Address on approval:<br>Sjaellandsgade 40 DK-2200 Koebenhavn N	Jens Bülow	25-10-02	11-07-02	12-08-03	21-07-07 (Approval from the Danish Health Authority)	08-06-07
811 / Finn VejlØ Rasmussen	Current address:<br>De Videnskabsetiske Komitéer for Region Hovedstaden Regionsgaarden Kongens Vaenge 2 DK-3400 Hilleroed	Jens Bülow	25-10-02	11-07-02	12-08-03	21-07-07 (Approval from the Danish Health Authority)	08-06-07

Denmark							
**Centre number(s) / Investigator(s)**	**IRB or IEC (name/address)**	**IRB or IEC Chairperson**	**Informed Consent/Pt. Information Form Approval DD/MM/YY**	**Protocol Approval DD/MM/YY**	**Am. 1 Approval DD/MM/YY**	**Am. 2 Approval DD/MM/YY**	**Am. 3 Approval DD/MM/YY**
	Address on approval:<br>Sjaellandsgade 40<br>DK-2200<br>Koebenhavn N						
812 / Mogens Christensen	Current address:<br>De Videnskabsetiske Komitéer for Region Hovedstaden<br>Regionsgaarden<br>Kongens Vaenge 2<br>DK-3400 Hilleroed<br><br>Address on approval:<br>Sjaellandsgade 40<br>DK-2200<br>Koebenhavn N	Jens Bülow	25-10-02	11-07-02	N/A<br>Center closed	N/A<br>Center closed	N/A<br>Center closed
813 / Kristen Stax Jakobsen	Current address:<br>De Videnskabsetiske Komitéer for Region Hovedstaden<br>Regionsgaarden<br>Kongens Vaenge 2<br>DK-3400 Hilleroed<br><br>Address on approval:<br>Sjaellandsgade 40<br>DK-2200<br>Koebenhavn N	Jens Bülow	25-10-02	11-07-02	12-08-03	21-07-07 (Approval from the Danish Health Authority)	08-06-07
814 / Jost Wessels	Current address:<br>De Videnskabsetiske Komitéer for Region Hovedstaden	Jens Bülow	25-10-02	11-07-02	12-08-03	21-07-07 (Approval from the Danish	08-06-07

Denmark							
**Centre number(s) / Investigator(s)**	**IRB or IEC (name/address)**	**IRB or IEC Chairperson**	**Informed Consent/Pt. Information Form Approval DD/MM/YY**	**Protocol Approval DD/MM/YY**	**Am. 1 Approval DD/MM/YY**	**Am. 2 Approval DD/MM/YY**	**Am. 3 Approval DD/MM/YY**
	Regionsgaarden<br>Kongens Vaenge 2<br>DK-3400 Hilleroed<br><br>Address on approval:<br>Sjaellandsgade 40<br>DK-2200<br>Koebenhavn N					Health Authority)	
815 / Jens L. Frandsen	Current address:<br>De Videnskabsetiske Komitéer for Region Hovedstaden<br>Regionsgaarden<br>Kongens Vaenge 2<br>DK-3400 Hilleroed<br><br>Address on approval:<br>Sjaellandsgade 40<br>DK-2200<br>Koebenhavn N	Jens Bülow	25-10-02	11-07-02	12-08-03	21-07-07 (Approval from the Danish Health Authority)	08-06-07
816 / Torben Evald	Current address:<br>De Videnskabsetiske Komitéer for Region Hovedstaden<br>Regionsgaarden<br>Kongens Vaenge 2<br>DK-3400 Hilleroed<br><br>Address on approval:<br>Sjaellandsgade 40<br>DK-2200<br>Koebenhavn N	Jens Bülow	25-10-02	11-07-02	12-08-03	21-07-07 (Approval from the Danish Health Authority)	08-06-07

Denmark							
**Centre number(s) / Investigator(s)**	**IRB or IEC (name/address)**	**IRB or IEC Chairperson**	**Informed Consent/Pt. Information Form Approval DD/MM/YY**	**Protocol Approval DD/MM/YY**	**Am. 1 Approval DD/MM/YY**	**Am. 2 Approval DD/MM/YY**	**Am. 3 Approval DD/MM/YY**
819 / Johnna Steentoft	Current address:<br>De Videnskabsetiske Komitéer for Region Hovedstaden<br>Regionsgaarden<br>Kongens Vaenge 2<br>DK-3400 Hilleroed<br><br>Address on approval:<br>Sjaellandsgade 40<br>DK-2200<br>Koebenhavn N	Jens Bülow	25-10-02	11-07-02	12-08-03	21-07-07 (Approval from the Danish Health Authority)	08-06-07
820 / Per Garsdal	Current address:<br>De Videnskabsetiske Komitéer for Region Hovedstaden<br>Regionsgaarden<br>Kongens Vaenge 2<br>DK-3400 Hilleroed<br><br>Address on approval:<br>Sjaellandsgade 40<br>DK-2200<br>Koebenhavn N	Jens Bülow	25-10-02	11-07-02	12-08-03	N/A Center Closed	N/A Center Closed
821 / Carl Nielsen	Current address:<br>De Videnskabsetiske Komitéer for Region Hovedstaden<br>Regionsgaarden<br>Kongens Vaenge 2<br>DK-3400 Hilleroed	Jens Bülow	25-10-02	11-07-02	12-08-03	21-07-07 (Approval from the Danish Health Authority)	08-06-07

Denmark							
**Centre number(s) / Investigator(s)**	**IRB or IEC (name/address)**	**IRB or IEC Chairperson**	**Informed Consent/Pt. Information Form Approval DD/MM/YY**	**Protocol Approval DD/MM/YY**	**Am. 1 Approval DD/MM/YY**	**Am. 2 Approval DD/MM/YY**	**Am. 3 Approval DD/MM/YY**
	Address on approval:<br>Sjaellandsgade 40<br>DK-2200<br>Koebenhavn N						
822 / Svend Haahr	Current address:<br>De Videnskabsetiske Komitéer for Region Hovedstaden<br>Regionsgaarden<br>Kongens Vaenge 2<br>DK-3400 Hilleroed<br><br>Address on approval:<br>Sjaellandsgade 40<br>DK-2200<br>Koebenhavn N	Jens Bülow	25-10-02	11-07-02	12-08-03	21-07-07 (Approval from the Danish Health Authority)	08-06-07
824 / Povl Arne Revsbech	Current address:<br>De Videnskabsetiske Komitéer for Region Hovedstaden<br>Regionsgaarden<br>Kongens Vaenge 2<br>DK-3400 Hilleroed<br><br>Address on approval:<br>Sjaellandsgade 40<br>DK-2200<br>Koebenhavn N	Jens Bülow	25-10-02	11-07-02	12-08-03	21-07-07 (Approval from the Danish Health Authority)	08-06-07
826 / Jens Georg Hansen	Current address:<br>De Videnskabsetiske Komitéer for Region Hovedstaden	Jens Bülow	25-10-02	11-07-02	12-08-03	21-07-07 (Approval from the Danish	08-06-07

Denmark							
**Centre number(s) / Investigator(s)**	**IRB or IEC (name/address)**	**IRB or IEC Chairperson**	**Informed Consent/Pt. Information Form Approval DD/MM/YY**	**Protocol Approval DD/MM/YY**	**Am. 1 Approval DD/MM/YY**	**Am. 2 Approval DD/MM/YY**	**Am. 3 Approval DD/MM/YY**
	Regionsgaarden<br>Kongens Vaenge 2<br>DK-3400 Hilleroed<br><br>Address on approval:<br>Sjaellandsgade 40<br>DK-2200<br>Koebenhavn N					Health Authority)	
827 / Torben Soerensen	Current address:<br>De Videnskabsetiske Komitéer for Region Hovedstaden<br>Regionsgaarden<br>Kongens Vaenge 2<br>DK-3400 Hilleroed<br><br>Address on approval:<br>Sjaellandsgade 40<br>DK-2200<br>Koebenhavn N	Jens Bülow	25-10-02	11-07-02	12-08-03	21-07-07 (Approval from the Danish Health Authority)	08-06-07
828 / John Rützou Arnved	Current address:<br>De Videnskabsetiske Komitéer for Region Hovedstaden<br>Regionsgaarden<br>Kongens Vaenge 2<br>DK-3400 Hilleroed<br><br>Address on approval:<br>Sjaellandsgade 40<br>DK-2200<br>Koebenhavn N	Jens Bülow	25-10-02	11-07-02	12-08-03	21-07-07 (Approval from the Danish Health Authority)	08-06-07

Denmark							
**Centre number(s) / Investigator(s)**	**IRB or IEC (name/address)**	**IRB or IEC Chairperson**	**Informed Consent/Pt. Information Form Approval DD/MM/YY**	**Protocol Approval DD/MM/YY**	**Am. 1 Approval DD/MM/YY**	**Am. 2 Approval DD/MM/YY**	**Am. 3 Approval DD/MM/YY**
830 / Axel MØller	Current address:<br>De Videnskabsetiske Komitéer for Region Hovedstaden<br>Regionsgaarden<br>Kongens Vaenge 2<br>DK-3400 Hilleroed<br><br>Address on approval:<br>Sjaellandsgade 40<br>DK-2200<br>Koebenhavn N	Jens Bülow	25-10-02	11-07-02	12-08-03	21-07-07 (Approval from the Danish Health Authority)	08-06-07
831 / Henriette Enevoldsen	Current address:<br>De Videnskabsetiske Komitéer for Region Hovedstaden<br>Regionsgaarden<br>Kongens Vaenge 2<br>DK-3400 Hilleroed<br>Address on approval:<br>Sjaellandsgade 40<br>DK-2200<br>Koebenhavn N	Jens Bülow	25-10-02	11-07-02	12-08-03	21-07-07 (Approval from the Danish Health Authority)	08-06-07
832 / Martin DØssing	Current address:<br>De Videnskabsetiske Komitéer for Region Hovedstaden<br>Regionsgaarden<br>Kongens Vaenge 2<br>DK-3400 Hilleroed<br>Address on approval:<br>Sjaellandsgade 40	Jens Bülow	25-10-02	11-07-02	12-08-03	21-07-07 (Approval from the Danish Health Authority)	08-06-07

Denmark							
Centre number(s) / Investigator(s)	IRB or IEC (name/address)	IRB or IEC Chairperson	Informed Consent/Pt. Information Form Approval DD/MM/YY	Protocol Approval DD/MM/YY	Am. 1 Approval DD/MM/YY	Am. 2 Approval DD/MM/YY	Am. 3 Approval DD/MM/YY
	DK-2200 Koebenhavn N						
833 / Peter Christensen	Current address: De Videnskabsetiske Komitéer for Region Hovedstaden Regionsgaarden Kongens Vaenge 2 DK-3400 Hilleroed Address on approval: Sjaellandsgade 40 DK-2200 Koebenhavn N	Jens Bülow	25-10-02	11-07-02	12-08-03	21-07-07 (Approval from the Danish Health Authority)	08-06-07
834 / Kent Dencker Christensen	Current address: De Videnskabsetiske Komitéer for Region Hovedstaden Regionsgaarden Kongens Vaenge 2 DK-3400 Hilleroed Address on approval: Sjaellandsgade 40 DK-2200 Koebenhavn N	Jens Bülow	25-10-02	11-07-02	12-08-03	21-07-07 (Approval from the Danish Health Authority)	08-06-07
835 / Lars FrØlund	Current address: De Videnskabsetiske Komitéer for Region Hovedstaden Regionsgaarden Kongens Vaenge 2 DK-3400 Hilleroed	Jens Bülow	25-10-02	11-07-02	12-08-03	21-07-07 (Approval from the Danish Health Authority)	08-06-07

Denmark							
**Centre number(s) / Investigator(s)**	**IRB or IEC (name/address)**	**IRB or IEC Chairperson**	**Informed Consent/Pt. Information Form Approval DD/MM/YY**	**Protocol Approval DD/MM/YY**	**Am. 1 Approval DD/MM/YY**	**Am. 2 Approval DD/MM/YY**	**Am. 3 Approval DD/MM/YY**
	Address on approval: Sjaellandsgade 40 DK-2200 Koebenhavn N						

Finland							
**Centre number(s) / Investigator(s)**	**IRB or IEC (name/address)**	**IRB or IEC Chairperson**	**Informed Consent/Pt. Information Form Approval DD/MM/YY**	**Protocol Approval DD/MM/YY**	**Am. 1 Approval DD/MM/YY**	**Am. 2 Approval DD/MM/YY**	**Am. 3 Approval DD/MM/YY**
901 / Kari Venho	Keski-Suomen Sairaanhoitopiiri, Eettinen toimikunta	Vuokko Rauhala<br>Jukka Puolakka<br>Raisa Lounama	06/08/02	20/10/02	-	01/03/06	28/05/07
902 / Matti Pietiläinen				20/10/02	-	06/03/06	22/05/07
903 / Pekka Saarelainen	Keski-Suomen Sairaanhoitopiiri, Eettinen toimikunta	Raisa Lounama	21/05/02	20/10/02	-	03/03/06	28/06/07
904 / Jyrki Kotaniemi	Päijät-Hämeen sairaanhoitopiirin kuntayhtymä, Eettinen toimikunta	Markku Luostarinen	20/11/02	28/10/02	-	06/03/06	25/05/07
905 / Jussi Männistö	Kymenlaakson sairaanhoitopiirin Eettinen toimikunta	Jaakko Johansson	18/11/02	29/10/02	-	03/03/06	25/05/07

France							
**Centre number(s) / Investigator(s)**	**IRB or IEC (name/address)**	**IRB or IEC Chairperson**	**Informed Consent/Pt. Information Form Approval DD/MM/YY**	**Protocol Approval DD/MM/YY**	**Am. 1 Approval DD/MM/YY**	**Am. 2 Approval DD/MM/YY**	**Am. 3 Approval DD/MM/YY**
1001 / Michel Aubier	C.P.P. Ile de France I Hôpital Hôtel Dieu 1, place du Parvis Notre-Dame 75181 PARIS Cedex 04	Docteur Elisabeth FRIJA	30/04/02	30/04/02	04/08/03	12/12/05	20/08/07
1002 / Bernard Pigearias	same	Docteur Elisabeth FRIJA	30/04/02	30/04/2002	04/08/03	12/12/05	20/08/07
1004 / François Malaquin	same	Docteur Elisabeth FRIJA	30/04/02	30/04/02	04/08/03	12/12/05	20/08/07
1005 / Noël Grunchec	same	Docteur Elisabeth FRIJA	30/04/02	30/04/02	04/08/03	12/12/05	20/08/07
1006 / Lucien Bernabeu	same	Docteur Elisabeth FRIJA	30/04/02	30/04/02	04/08/03	12/12/05	20/08/07
1007 / Bernard Carme	same	Docteur Elisabeth FRIJA	30/04/02	30/04/02	04/08/03	12/12/05	20/08/07
1009 / Anne Prudhomme	same	Docteur Elisabeth FRIJA	30/04/02	30/04/02	04/08/03	12/12/05	20/08/07
1012 / François Bonte	same	Docteur Elisabeth FRIJA	30/04/02	30/04/02	04/08/03	12/12/05	20/08/07
1013 / Dominique Boz	same	Docteur Elisabeth FRIJA	30/04/02	30/04/02	04/08/03	12/12/05	20/08/07

France							
**Centre number(s) / Investigator(s)**	**IRB or IEC (name/address)**	**IRB or IEC Chairperson**	**Informed Consent/Pt. Information Form Approval DD/MM/YY**	**Protocol Approval DD/MM/YY**	**Am. 1 Approval DD/MM/YY**	**Am. 2 Approval DD/MM/YY**	**Am. 3 Approval DD/MM/YY**
1015 / Richard Meunier	same	Docteur Elisabeth FRIJA	30/04/02	30/04/02	04/08/03	12/12/05	20/08/07
1016 / Marie-Thérèse Rasolojaona	same	Docteur Elisabeth FRIJA	30/04/02	30/04/02	04/08/03	12/12/05	20/08/07
1019 / Michel Apprill	same	Docteur Elisabeth FRIJA	30/04/02	30/04/02	04/08/03	12/12/05	20/08/07
1020 / Roland Fargeon	same	Docteur Elisabeth FRIJA	30/04/02	30/04/02	04/08/03	12/12/05	20/08/07
1021 / Patrick Bernard	same	Docteur Elisabeth FRIJA	30/04/02	30/04/02	04/08/03	12/12/05	20/08/07
1023 / François Tirouvanziam	same	Docteur Elisabeth FRIJA	30/04/02	30/04/02	04/08/03	12/12/05	20/08/07
1024 / Marc Legendre	same	Docteur Elisabeth FRIJA	30/04/02	30/04/02	04/08/03	12/12/05	20/08/07
1025 / Jean-Yves Jasnot	same	Docteur Elisabeth FRIJA	30/04/02	30/04/02	04/08/03	12/12/05	20/08/07

Germany							
**Centre number(s) / Investigator(s)**	**IRB or IEC (name/address)**	**IRB or IEC Chairperson**	**Informed Consent/Pt. Information Form Approval DD/MM/YY**	**Protocol Approval DD/MM/YY**	**Am. 1 Approval DD/MM/YY**	**Am. 2 Approval DD/MM/YY**	**Am. 3 Approval DD/MM/YY**
1101 / Michael Barczok	Landesaerztekammer Baden-Wuerttemberg Jahnstrasse 40 70597 Stuttgart	PD Dr. med. R. Zwirner	08/04/02 06/06/02 22/07/02	08/04/02	04/07/03	21/01/06	11/05/07 (notice)
1102 / Jens Becker	Ethikkommission der Aerztekammer Schleswig-Holstein Bismarckallee 8-12 23795 Bad Segeberg		same	09/09/02	04/07/03 (Landesaerztekammer Baden-Wuerttemberg)	21/01/06 (Landesaerztekammer Baden-Wuerttemberg)	11/05/07 (notice) (Landesaerztekammer Baden-Wuerttemberg)
1103 / Mark Pilz	Aerztekammer Niedersachsen Berliner Allee 20 30175 Hannover		same	10/09/02	04/07/03 (Landesaerztekammer Baden-Wuerttemberg)	21/01/06 (Landesaerztekammer Baden-Wuerttemberg)	11/05/07 (notice)(Landesaerztekammer Baden-Wuerttemberg)
1104 / Vera Grimm-Sachs	Landesaerztekammer Baden-Wuerttemberg Jahnstrasse 40 70597 Stuttgart	PD Dr. med. R. Zwirner	08/04/02 06/06/02 22/07/02	08/04/02	04/07/03	21/01/06	11/05/07 (notice)
1105 / Martin Jahn	Aerztekammer Niedersachsen Berliner Allee 20 30175 Hannover		same	10/09/02	04/07/03 (Landesaerztekammer Baden-Wuerttemberg)	21/01/06 (Landesaerztekammer Baden-Wuerttemberg)	11/05/07 (notice) (Landesaerztekammer Baden-Wuerttemberg)
1106 / Bernd Kemmerich	Bayerische Landesaerztekammer Mühlbaurstrasse 16 81677 Muenchen		same	27/08/02	04/07/03 (Landesaerztekammer Baden-Wuerttemberg)	21/01/06 (Landesaerztekammer Baden-Wuerttemberg)	11/05/07 (notice) (Landesaerztekammer Baden-Wuerttemberg)

Germany							
**Centre number(s) / Investigator(s)**	**IRB or IEC (name/address)**	**IRB or IEC Chairperson**	**Informed Consent/Pt. Information Form Approval DD/MM/YY**	**Protocol Approval DD/MM/YY**	**Am. 1 Approval DD/MM/YY**	**Am. 2 Approval DD/MM/YY**	**Am. 3 Approval DD/MM/YY**
1107 / Walter Vorderstrasse	Aerztekammer Niedersachsen Berliner Allee 20 30175 Hannover		same	10/09/02	04/07/03 (Landesaerztekammer Baden-Wuerttemberg)	21/01/06 (Landesaerztekammer Baden-Wuerttemberg)	11/05/07 (notice) (Landesaerztekammer Baden-Wuerttemberg)
1108 / Gerhard Klein	Landesaerztekammer Baden-Wuerttemberg Jahnstrasse 40 70597 Stuttgart	PD Dr. med. R. Zwirner	08/04/02 06/06/02 22/07/02	08/04/02	04/07/03	21/01/06	11/05/07 (notice)
1109 / Bernd Kroemer	Bayerische Landesaerztekammer Mühlbaurstrasse 16 81677 Muenchen		same	27/08/02	04/07/03 (Landesaerztekammer Baden-Wuerttemberg)	21/01/06 (Landesaerztekammer Baden-Wuerttemberg)	11/05/07 (notice)(Landesaerztekammer Baden-Wuerttemberg)
1110 / Joachim Lehnert	Bayerische Landesaerztekammer Mühlbaurstrasse 16 81677 Muenchen		same	27/08/02	04/07/03 (Landesaerztekammer Baden-Wuerttemberg)	21/01/06 (Landesaerztekammer Baden-Wuerttemberg)	11/05/07 (notice) (Landesaerztekammer Baden-Wuerttemberg)
1112 / Ronald Redlich	Landesaerztekammer Baden-Wuerttemberg Jahnstrasse 40 70597 Stuttgart	PD Dr. med. R. Zwirner	08/04/02 06/06/02 22/07/02	08/04/02	04/07/03	21/01/06	11/05/07 (notice)
1113 / Sören Schmidtmann	Äerztekammer Berlin Flottenstrasse 28-42 13407 Berlin		same	20/08/02	04/07/03 (Landesaerztekammer Baden-Wuerttemberg)	21/01/06 (Landesaerztekammer Baden-Wuerttemberg)	11/05/07 (notice) (Landesaerztekammer Baden-Wuerttemberg)

Germany							
**Centre number(s) / Investigator(s)**	**IRB or IEC (name/address)**	**IRB or IEC Chairperson**	**Informed Consent/Pt. Information Form Approval DD/MM/YY**	**Protocol Approval DD/MM/YY**	**Am. 1 Approval DD/MM/YY**	**Am. 2 Approval DD/MM/YY**	**Am. 3 Approval DD/MM/YY**
1114 / Thomas Schultz	Äerztekammer Berlin Flottenstrasse 28-42 13407 Berlin		ame	20/08/02	04/07/03 (Landesaerztekammer Baden-Wuerttemberg)	21/01/06 (Landesaerztekammer Baden-Wuerttemberg)	11/05/07 (notice) (Landesaerztekammer Baden-Wuerttemberg)
1115 / Hans-Henning Weber	Aerztekammer Niedersachsen Berliner Allee 20 30175 Hannover		same	10/09/02	04/07/03 (Landesaerztekammer Baden-Wuerttemberg)	21/01/06 (Landesaerztekammer Baden-Wuerttemberg)	11/05/07 (notice) (Landesaerztekammer Baden-Wuerttemberg)

Greece							
**Centre number(s) / Investigator(s)**	**IRB or IEC (name/address)**	**IRB or IEC Chairperson**	**Informed Consent/Pt. Information Form Approval DD/MM/YY**	**Protocol Approval DD/MM/YY**	**Am. 1 Approval DD/MM/YY**	**Am. 2 Approval DD/MM/YY**	**Am. 3 Approval DD/MM/YY**
1201 / Nicolaos Siafakas	National Organization of Medicines/ Mesogeion Avenue 284, 155 62 Xolargos	M. Marselos	Same as the protocol	17/JUN/02	Not applicable for Greece	21/AUG/06	14/SEP/07
1202 / Ourania Anagnostopoulou	National Organization of Medicines/ Mesogeion Avenue 284, 155 62 Xolargos	M. Marselos	Same as the protocol	17/JUN/02	Not applicable for Greece	21/AUG/06	14/SEP/07
1203 / Michail Toubis	National Organization of Medicines/ Mesogeion Avenue 284, 155 62 Xolargos	M. Marselos	Same as the protocol	17/JUN/02	Not applicable for Greece	21/AUG/06	14/SEP/07
1204 / Konstantinos Gourgoulianis	National Organization of Medicines/ Mesogeion Avenue 284, 155 62 Xolargos	M. Marselos	Same as the protocol	17/JUN/02	Not applicable for Greece	16/OCT/07	14/SEP/07
1205 / Lazaros Sichletides	National Organization of Medicines/ Mesogeion Avenue 284, 155 62 Xolargos	M. Marselos	Same as the protocol	17/JUN/02	Not applicable for Greece	21/AUG/06	14/SEP/07
1206 / Marianna Kakoura	National Organization of Medicines/ Mesogeion Avenue 284, 155 62 Xolargos	M. Marselos	Same as the protocol	17/JUN/02	Not applicable for Greece	21/AUG/06	14/SEP/07
1207 / Pandora Christaki	National Organization of Medicines/ Mesogeion Avenue 284, 155 62 Xolargos	M. Marselos	Same as the protocol	17/JUN/02	Not applicable for Greece	21/AUG/06	14/SEP/07
1209 / Ioannis Lychros	National Organization of Medicines/ Mesogeion Avenue 284, 155 62 Xolargos	M. Marselos	Same as the protocol	17/JUN/02	Not applicable for Greece	21/AUG/06	14/SEP/07

Greece							
**Centre number(s) / Investigator(s)**	**IRB or IEC (name/address)**	**IRB or IEC Chairperson**	**Informed Consent/Pt. Information Form Approval DD/MM/YY**	**Protocol Approval DD/MM/YY**	**Am. 1 Approval DD/MM/YY**	**Am. 2 Approval DD/MM/YY**	**Am. 3 Approval DD/MM/YY**
1210 / Konstantinos Spiropoulos	National Organization of Medicines/ Mesogeion Avenue 284, 155 62 Xolargos	M. Marselos	Same as the protocol	17/JUN/02	Not applicable for Greece	21/AUG/06	14/SEP/07
1211 / Stavroula Bousmoukilia	National Organization of Medicines/ Mesogeion Avenue 284, 155 62 Xolargos	M. Marselos	Same as the protocol	17/JUN/02	Not applicable for Greece	21/AUG/06	14/SEP/07

Greece							
**Centre number(s) / Investigator(s)**	**IRB or IEC (name/address)**	**IRB or IEC Chairperson**	**Informed Consent/Pt. Information Form Approval DD/MM/YY**	**Protocol Approval DD/MM/YY**	**Am. 1 Approval DD/MM/YY**	**Am. 2 Approval DD/MM/YY**	**Am. 3 Approval DD/MM/YY**
**Centre number(s) / Investigator(s)**	**IRB or IEC (name/address)**	**IRB or IEC Chairperson**	**Informed Consent/Pt. Information Form Approval DD/MM/YY**	**Protocol Approval DD/MM/YY**	**Am. 1 Approval DD/MM/YY**	**Am. 2 Approval DD/MM/YY**	**Am. 3 Approval DD/MM/YY**
1401 / David S C Hui	Joint The Chinese University of Hong Kong – New Territories East Cluster Clinical Research Ethics Committee<br><br>Address: Flat 3C, Blk B, Staff Quarter, Prince of Wales Hospital, Shatin, N.T., Hong Kong	Prof. Joseph Lau	Patient Information and Written Informed Consent Form, English Ver, HK No.1 dated 26Mar2002 Approval date 24/06/02<br><br>Patient Information and Written Informed Consent Form, Chinese Ver, HK No.1 dated 15Apr2002 Approval date 24/06/02<br><br>Patient Information and Written Informed Consent Form, English Ver, HK No.2 dated	24/06/02	NA	09/05/06	08/06/07

Greece							
Centre number(s) / Investigator(s)	IRB or IEC (name/address)	IRB or IEC Chairperson	Informed Consent/Pt. Information Form Approval DD/MM/YY	Protocol Approval DD/MM/YY	Am. 1 Approval DD/MM/YY	Am. 2 Approval DD/MM/YY	Am. 3 Approval DD/MM/YY
			3Dec2004 Approval date 04/01/05<br><br>Patient Information and Written Informed Consent Form, Chinese Ver, HK No.2 dated 3Dec2004 Approval date 04/01/05<br><br>Supplemental Informed Consent – Long Term Follow up, English Ver 1.0, dated 30Nov2005 Approval date 09/05/06<br><br>Supplemental Informed Consent – Long Term Follow up, Chinese Chinse Ver 1.0, dated 28Mar2006 Approval date 09/05/06				

Greece							
**Centre number(s) / Investigator(s)**	**IRB or IEC (name/address)**	**IRB or IEC Chairperson**	**Informed Consent/Pt. Information Form Approval DD/MM/YY**	**Protocol Approval DD/MM/YY**	**Am. 1 Approval DD/MM/YY**	**Am. 2 Approval DD/MM/YY**	**Am. 3 Approval DD/MM/YY**
			Patient Information and Written Informed Consent Form, Chinese Ver, HK No.3 dated 15Jun2006 Approval date 13/10/06<br><br>Patient Information and Written Informed Consent Form, Chinese Ver, HK No.3 dated 15Jun2006 Approval date 13/10/06<br><br>Addendum to Patient Information and Written Informed Consent Form – English Ver, dated 11Dec2006 Approval date 09/01/07<br><br>Addendum to				

Greece							
**Centre number(s) / Investigator(s)**	**IRB or IEC (name/address)**	**IRB or IEC Chairperson**	**Informed Consent/Pt. Information Form Approval DD/MM/YY**	**Protocol Approval DD/MM/YY**	**Am. 1 Approval DD/MM/YY**	**Am. 2 Approval DD/MM/YY**	**Am. 3 Approval DD/MM/YY**
			Patient Information and Written Informed Consent Form – Traditional Chinese Ver, dated 11Dec2006 Approval date 09/01/07				
1402 / Wai Cho Yu	Clinical Research Ethics Committee Kowloon West Cluster<br><br>Address: Rm133, Block J, Princess Margaret Hospital, Lai Chi Kok, Kowloon, N.T., Hong Kong.	Dr. Yen-Chow TSAO	Patient Information and Written Informed Consent Form, English Ver, HK No.1 dated 26Mar2002 Approval date 17/07/02<br><br>Patient Information and Written Informed Consent Form, Chinese Ver, HK No.1 dated 15Apr2002 Approval date 17/07/02<br><br>Patient Information and Written Informed	17/07/02	NA	04/10/2005	30/05/07

Greece							
**Centre number(s) / Investigator(s)**	**IRB or IEC (name/address)**	**IRB or IEC Chairperson**	**Informed Consent/Pt. Information Form Approval DD/MM/YY**	**Protocol Approval DD/MM/YY**	**Am. 1 Approval DD/MM/YY**	**Am. 2 Approval DD/MM/YY**	**Am. 3 Approval DD/MM/YY**
			Consent Form, English Ver, HK No.2 dated 3Dec2004 Approval date 04/10/2005<br><br>Patient Information and Written Informed Consent Form, Chinese Ver, HK No.2 dated 3Dec2004 Approval date 04/10/2005<br><br>Supplemental Informed Consent – Long Term Follow up, English Ver 1.0, dated 30Nov2005 Approval date 18/08/06<br><br>Supplemental Informed Consent – Long Term Follow up, Chinese Chinse Ver 1.0, dated				

Greece							
**Centre number(s) / Investigator(s)**	**IRB or IEC (name/address)**	**IRB or IEC Chairperson**	**Informed Consent/Pt. Information Form Approval DD/MM/YY**	**Protocol Approval DD/MM/YY**	**Am. 1 Approval DD/MM/YY**	**Am. 2 Approval DD/MM/YY**	**Am. 3 Approval DD/MM/YY**
			28Mar2006 Approval date 18/08/06<br><br>Patient Information and Written Informed Consent Form, English Ver, HK No.3 dated 15Jun2006 Approval date 07/11/06<br><br>Patient Information and Written Informed Consent Form, Chinese Ver, HK No.3 dated 15Jun2006 Approval date 07/11/06<br><br>Patient Information and Written Informed Consent Form, Chinese Ver, HK No.3 dated 15Jun2006				

Greece							
**Centre number(s) / Investigator(s)**	**IRB or IEC (name/address)**	**IRB or IEC Chairperson**	**Informed Consent/Pt. Information Form Approval DD/MM/YY**	**Protocol Approval DD/MM/YY**	**Am. 1 Approval DD/MM/YY**	**Am. 2 Approval DD/MM/YY**	**Am. 3 Approval DD/MM/YY**
			Approval date 20/09/06<br><br>Patient Information and Written Informed Consent Form, Chinese Ver, HK No.3 dated 15Jun2006 Approval date 20/09/06<br><br>Addendum to Patient Information and Written Informed Consent Form – English Ver, dated 11Dec2006 Approval date 02/02/07<br><br>Addendum to Patient Information and Written Informed Consent Form – Traditional Chinese Ver, dated				

Greece							
**Centre number(s) / Investigator(s)**	**IRB or IEC (name/address)**	**IRB or IEC Chairperson**	**Informed Consent/Pt. Information Form Approval DD/MM/YY**	**Protocol Approval DD/MM/YY**	**Am. 1 Approval DD/MM/YY**	**Am. 2 Approval DD/MM/YY**	**Am. 3 Approval DD/MM/YY**
			11Dec2006 Approval date 02/02/07				
1403 / Hok Sum Chan	Joint The Chinese University of Hong Kong – New Territories East Cluster Clinical Research Ethics Committee<br><br>Address: Flat 3C, Blk B, Staff Quarter, Prince of Wales Hospital, Shatin, N.T., Hong Kong	Prof. Joseph Lau	Patient Information and Written Informed Consent Form, English Ver, HK No.1 dated 26Mar2002 Approval date 19/07/02<br><br>Patient Information and Written Informed Consent Form, Chinese Ver, HK No.1 dated 15Apr2002 Approval date 19/07/02<br><br>Supplemental Informed Consent – Long Term Follow up, English Ver 1.0, dated 30Nov2005 Approval date 06/02/07	19/07/02	NA	06/02/07	Not available

Greece							
**Centre number(s) / Investigator(s)**	**IRB or IEC (name/address)**	**IRB or IEC Chairperson**	**Informed Consent/Pt. Information Form Approval DD/MM/YY**	**Protocol Approval DD/MM/YY**	**Am. 1 Approval DD/MM/YY**	**Am. 2 Approval DD/MM/YY**	**Am. 3 Approval DD/MM/YY**
			Supplemental Informed Consent – Long Term Follow up, Traditional Chinse Ver 1.0, dated 28Mar2006 Approval date 06/02/07<br><br>Patient Information and Written Informed Consent Form, English Ver, HK No.3 dated 15Jun2006 Approval date 06/02/07<br><br>Patient Information and Written Informed Consent Form, Chinese Ver, HK No.3 dated 15Jun2006 Approval date 06/02/07				

Greece							
**Centre number(s) / Investigator(s)**	**IRB or IEC (name/address)**	**IRB or IEC Chairperson**	**Informed Consent/Pt. Information Form Approval DD/MM/YY**	**Protocol Approval DD/MM/YY**	**Am. 1 Approval DD/MM/YY**	**Am. 2 Approval DD/MM/YY**	**Am. 3 Approval DD/MM/YY**
1404 / Mo Lin M. L. Wong	Caritas Medical Centre Ethics Committee<br><br>Address: Caritas Medical Centre, Shamshuipo, Kowloon, HK.	Dr. Helen TINSLEY	Patient Information and Written Informed Consent Form, English Ver, HK No.1 dated 26Mar2002 Approval date 23/09/02<br><br>Patient Information and Written Informed Consent Form, Chinese Ver, HK No.1 dated 15Apr2002 Approval date 23/09/02<br><br>Patient Information and Written Informed Consent Form, English Ver, HK No.2 dated 3Dec2004 Approval date 26/01/2005<br><br>Patient	23/09/02	NA	10/05/06	06/06/07

Greece							
**Centre number(s) / Investigator(s)**	**IRB or IEC (name/address)**	**IRB or IEC Chairperson**	**Informed Consent/Pt. Information Form Approval DD/MM/YY**	**Protocol Approval DD/MM/YY**	**Am. 1 Approval DD/MM/YY**	**Am. 2 Approval DD/MM/YY**	**Am. 3 Approval DD/MM/YY**
			Information and Written Informed Consent Form, Chinese Ver, HK No.2 dated 3Dec2004 Approval date 26/01/2005<br><br>Supplemental Informed Consent – Long Term Follow up, English Ver 1.0, dated 30Nov2005 Approval date 10/05/2006<br><br>Supplemental Informed Consent – Long Term Follow up, Traditional Chinse Ver 1.0, dated 28Mar2006 Approval date 10/05/2006<br><br>Patient Information and Written Informed				

Greece							
**Centre number(s) / Investigator(s)**	**IRB or IEC (name/address)**	**IRB or IEC Chairperson**	**Informed Consent/Pt. Information Form Approval DD/MM/YY**	**Protocol Approval DD/MM/YY**	**Am. 1 Approval DD/MM/YY**	**Am. 2 Approval DD/MM/YY**	**Am. 3 Approval DD/MM/YY**
			Consent Form, English Ver, HK No.3 dated 15Jun2006 Approval date 20/09/06<br><br>Patient Information and Written Informed Consent Form, Chinese Ver, HK No.3 dated 15Jun2006 Approval date 20/09/06<br><br>Addendum to Patient Information and Written Informed Consent, English Ver, dated 11Dec2006 Approval date 03/01/07<br>Addendum to Patient Information and Written Informed Consent, Traditional				

Greece							
**Centre number(s) / Investigator(s)**	**IRB or IEC (name/address)**	**IRB or IEC Chairperson**	**Informed Consent/Pt. Information Form Approval DD/MM/YY**	**Protocol Approval DD/MM/YY**	**Am. 1 Approval DD/MM/YY**	**Am. 2 Approval DD/MM/YY**	**Am. 3 Approval DD/MM/YY**
			Chinese Ver, dated 11Dec2006 Approval date 03/01/07				

Centre number(s) / Investigator(s)	IRB or IEC (name/address)	IRB or IEC Chairperson	Informed Consent/Pt. Information Form Approval DD/MM/YY	Protocol Approval DD/MM/YY	Am. 1 Approval DD/MM/YY	Am. 2 Approval DD/MM/YY	Am. 3 Approval DD/MM/YY
**Greece**							
**Hungary**							
Hungarian PI-IC version 1 (14 February 2002) was approved together with 1st submitted trial documents (included Protocol) by Local Ethics Committees.<br><br>2nd version of PI-IC (dated 09 Nov 2004) had been submitted to OGYI and to the LECs in January 2005. OGYI had requested for further modification in it, so a modified 2nd version of PI-IC (dated 30 March 2005) had been submitted to OGYI in April 2005 and had been later approved by them. This latter version was sent to all LECs again. That is both versions were submitted to LECs.<br>The LEC approvals are available either for version dated 09 Nov 2004 or for version dated 30 Mar 2005 depending on the date of the approval of the LEC.<br><br>From the 15th of September 2005 new local regulation became effective. See Decree 35/2005 (VIII.26.) of the Minister of Health (filed in the ISF section 12). According to this local regulation Protocol amendments and change of other study related documents have to be approved by the Regulatory Authority and Central Ethics Committee. Requests for change of any study related documents have to be submitted only to the Regulatory Authority who forwards documents to the Central Ethics Committee if they decide it is required. Central Ethic Committee issues its opinion only for the Regulatory Authority and not directly for the sponsor or investigators.<br>In case of Amendment 3 RA issued its approval without CEC review.<br><br>Central Ethics Committee in Hungary was: Egészségügyi Tudományos Tanács, Klinikai Farmakológiai Etikai Bizottsága, 1051 Budapes Arany J. u. 6-8.							
1301 / Zoltan Baliko	POTE Regionális Kutatásetikai Bizottsága Szigeti u. 12, Pécs 7643	Prof. Dr. Iván Kétyi	01-04-05<br>04-04-05	12-08-02	01-08-03<br>29-09-03	01-02-06	NA
1302 / Marta Bisits	Komárom-Esztergom Megyei Önormányzat Szent Borbála Kórháza, Etikai Bizottság Dózya Gy. u. 77., Tatabánya 2800	Dr. Gábor Nagy	09-02-05<br>25-02-05	30-10-02	13-08-03<br>28-08-03	01-02-06	NA
1303 / György Böszörményi	Országos Korányi TBC és Pulmonológiai Intézet	Dr. Márta Valyon	02-02-05<br>04-02-05	09-09-02	28-07-03<br>26-08-03	01-02-06	NA

Greece							
**Centre number(s) / Investigator(s)**	**IRB or IEC (name/address)**	**IRB or IEC Chairperson**	**Informed Consent/Pt. Information Form Approval DD/MM/YY**	**Protocol Approval DD/MM/YY**	**Am. 1 Approval DD/MM/YY**	**Am. 2 Approval DD/MM/YY**	**Am. 3 Approval DD/MM/YY**
	Intézményi Kutatásetikai Bizottsága Pihenő út 1., Budapest 1529						
1304 / Dr. Maria Marton	Regionális Tudomáynos Kutatásetikai Bizottság Borsod-Abaúj-Zemplén Megye Szentpéteri kapu 72-76., Miskolc 3501	Dr. László Szabó	11-02-05<br>28-04-07	09-07-02	04-08-03<br>28-04-07	01-02-06	NA
1305 / Karoly Fonay	Egészségügyi Tudományos Tanács Regionális Kutatásetikai Bizottsága Győr-Moson-Sopron és Komárom - Esztergom Megye Vasvári Pál u. 2, Győr 9002	Dr. György Ostorharics-Horváth	01-02-05<br>16-03-05	16-10-02	not sent back<br>30-09-03	01-02-06	NA
1306 / Zsuzsa Györi	Tüdőgyógyintézet Törökbálint Intézeti Kutatásetikai Bizottság Munkácsy M. u. 70., Törökbálint 2045	Dr. László Perger	01-02-05<br>04-02-08	19-06-02	18-08-03<br>26-08-03	01-02-06	NA
1307 / Laszlo Hajdu	Szegedi Tudomáynegyetem ÁOK, Regionális	Prof. Dr. Tibor Wittmann	31-01-05<br>25-04-05	05-11-02	18-08-03<br>03-10-03	01-02-06	NA

Greece							
**Centre number(s) / Investigator(s)**	**IRB or IEC (name/address)**	**IRB or IEC Chairperson**	**Informed Consent/Pt. Information Form Approval DD/MM/YY**	**Protocol Approval DD/MM/YY**	**Am. 1 Approval DD/MM/YY**	**Am. 2 Approval DD/MM/YY**	**Am. 3 Approval DD/MM/YY**
	Humán Orvosbiológiai Kutatásetikai Bizottsága Korányi fasor 8., Szeged 6720 untill 18 May 2006 Semmelweis Kórház Kutatásetikai Bizottsága Dr. Monszaprt L. u. 1., Kiskunhalas 6400 from 18 May 2006	Dr. Katalin Takács					
1308 / Judit Lukács	Szent János Kórház – Rendelőintézet Tudományos Bizottsága és IKEB Diósárok u. 1., Budapest 1125	Prof. Dr. András Jánosi	01-02-05<br>17-01-08	11-11-02	29-07-03<br>23-09-03	01-02-06	NA
1309 / Gabor Kovacs	Országos Korányi TBC és Pulmonológiai Intézet Intézményi Kutatásetikai Bizottsága Pihenő út 1., Budapest 1529	Dr. Márta Valyon	09-05-05<br>28-02-08	24-07-02	25-07-03<br>22-11-06	01-02-06	NA
1310 / Pal Magyar	Semmelweis Egyetem Regionális és Tudományos Kutatásetikai Bizottsága	Prof. Dr. Péter Sótonyi	11-02-05<br>30-05-05	21-10-02	28-08-03<br>07-10-03	01-02-06	NA

Greece							
**Centre number(s) / Investigator(s)**	**IRB or IEC (name/address)**	**IRB or IEC Chairperson**	**Informed Consent/Pt. Information Form Approval DD/MM/YY**	**Protocol Approval DD/MM/YY**	**Am. 1 Approval DD/MM/YY**	**Am. 2 Approval DD/MM/YY**	**Am. 3 Approval DD/MM/YY**
	Üllői út 93., Budapest 1091						
1311 / Gabriella Nagy	Szent Imre Kórház Intézményi Kutatásetikai Bizottság Tétényi út 12-16., Budapest 1125	Dr. Imre Orbán	04-02-05<br>09-03-05	11-09-02	05-08-03<br>03-09-03	site closed	site closed
1312 / Miklos Namenyi	Veszprém Megyei Csolnoky Ferenc Kórház-Rendelőintézet Etikai Bizottsága Kórház u. 1., Veszprém 8200 until 31 Dec 2003<br>Vas Megyei Önkormányzat Markusovszky Kórháza Regionális Tudományos Kutatásetikai Bizottság Markusovszky u. 3., Szombathely 9700 from 01 Jan 2004	Dr. Ferenc Brittig<br>Prof. Dr. Antal Salamon	14-02-05<br>26-09-07	04-06-04	Not sent back<br>26-07-03	01-02-06	NA
1313 / Laszlo Petö	Budai MÁV Kórház Etikai Bizottsága Szanatórium u. 2., Budapest 1528	Dr. Paróczai Karolin<br>Prof. Dr. Péter Sótonyi	25-02-05<br>28-06-07	22-11-02	31-07-03<br>28-06-07	01-02-06	NA

Greece							
**Centre number(s) / Investigator(s)**	**IRB or IEC (name/address)**	**IRB or IEC Chairperson**	**Informed Consent/Pt. Information Form Approval DD/MM/YY**	**Protocol Approval DD/MM/YY**	**Am. 1 Approval DD/MM/YY**	**Am. 2 Approval DD/MM/YY**	**Am. 3 Approval DD/MM/YY**
	until 10 Oct 2007<br>Semmelweis Egyetem Regionális és Tudományos Kutatásetikai Bizottsága<br>Üllői út 93., Budapest 1091<br>from 11 Oct 2007						
1314 / Attila Somfay	Szegedi Tudományegyetem ÁOK, Regionális Humán Orvosbiológiai Kutatásetikai Bizottsága<br>Korányi fasor 8., Szeged 6720	Prof. Dr. Tibor Wittmann	09-05-05<br>30-05-05	14-10-02	05-08-03<br>01-10-03	01-02-06	NA
1315 / Zsolt Kiraly	Vas Megyei Önkormányzat Markusovszky Kórháza Regionális Tudományos Kutatásetikai Bizottság<br>Markusovszky u. 3., Szombathely 9700	Prof. Dr. Antal Salamon	13-05-05<br>26-09-07	24-09-02	Not sent back<br>31-08-03	01-02-06	NA
1316 / Janos Strausz	Tüdőgyógyintézet Törökbálint<br>Intézeti Kutatásetikai Bizottság, Munkácsy	Dr. László Perger	01-02-05<br>25-11-05	19-06-02	18-08-03<br>26-08-03	01-02-06	NA

Greece							
**Centre number(s) / Investigator(s)**	**IRB or IEC (name/address)**	**IRB or IEC Chairperson**	**Informed Consent/Pt. Information Form Approval DD/MM/YY**	**Protocol Approval DD/MM/YY**	**Am. 1 Approval DD/MM/YY**	**Am. 2 Approval DD/MM/YY**	**Am. 3 Approval DD/MM/YY**
	M. u. 70., Törökbálint 2045						
1317 / Zsuzsanna Szalai	Karolina Kórház – Rendelőintézet Intézeti Kutatásetikai Bizottság, Régi Vámház tér 2-4., Mosonmagyaróvár 9200	Dr. Zoltán Várallyay	10-02-05<br>18-05-05	08-11-02	25-07-03<br>30-07-03	01-02-06	NA
1318 / Maria Szilasi	DEOEC Tudományos Bizottságának Regionális Kutatásetikai Bizottsága Nagyaerdei krt. 98., Debrecen 4012	Prof. Dr. Irén Horkay	15-02-05<br>31-03-05	24-06-02	24-09-03<br>29-09-03	01-02-06	NA
1319 / Barna Szima	Vas Megyei Önkormányzat Markusovszky Kórháza Regionális Tudományos Kutatásetikai Bizottság Markusovszky u. 3., Szombathely 9700	Prof. Dr. Antal Salamon	13-05-05<br>17-01-08	02-09-02	28-07-03<br>17-01-08	01-02-06	NA
1320 / Zsuzsa Sztancsik	Szegedi Tudományegyetem ÁOK, Regionális Humán Orvosbiológiai Kutatásetikai	Prof. Dr. Tibor Wittmann	09-05-05<br>30-05-05	14-10-02	01-08-03<br>01-10-03	01-02-06	NA

Greece							
**Centre number(s) / Investigator(s)**	**IRB or IEC (name/address)**	**IRB or IEC Chairperson**	**Informed Consent/Pt. Information Form Approval DD/MM/YY**	**Protocol Approval DD/MM/YY**	**Am. 1 Approval DD/MM/YY**	**Am. 2 Approval DD/MM/YY**	**Am. 3 Approval DD/MM/YY**
	Bizottsága Korányi fasor 8., Szeged 6720						

Ireland							
**Centre number(s) / Investigator(s)**	**IRB or IEC (name/address)**	**IRB or IEC Chairperson**	**Informed Consent/Pt. Information Form Approval DD/MM/YY**	**Protocol Approval DD/MM/YY**	**Am. 1 Approval DD/MM/YY**	**Am. 2 Approval DD/MM/YY**	**Am. 3 Approval DD/MM/YY**
1501 / Richard Costello	Beaumont Hospital Ethics Committee Beaumont Hospital Dublin 9 Ireland	Professor Gerry McElvaney	14/01/03	14/01/03	16/05/03	15/11/05	25/04/07
1503 / Seamas Donnelly	Ethics Committee St. Vincents Hospital Elm Park Dublin 4 Ireland	Dr. Douglas Veale	12/12/02	12/12/02	19/05/03	N/A	N/A
1505 / Conor Burke	Ethics Committee James Connolly Memorial Hospital Blanchardstown Dublin 15 Ireland	Dr. Conor Burke	04/02/03	04/02/03	21/05/03	15/11/05	25/04/07

Italy							
**Centre number(s) / Investigator(s)**	**IRB or IEC (name/address)**	**IRB or IEC Chairperson**	**Informed Consent/Pt. Information Form Approval DD/MM/YY**	**Protocol Approval DD/MM/YY**	**Am. 1 Approval DD/MM/YY**	**Am. 2 Approval DD/MM/YY**	**Am. 3 Approval DD/MM/YY**

Italy							
**Centre number(s) / Investigator(s)**	**IRB or IEC (name/address)**	**IRB or IEC Chairperson**	**Informed Consent/Pt. Information Form Approval DD/MM/YY**	**Protocol Approval DD/MM/YY**	**Am. 1 Approval DD/MM/YY**	**Am. 2 Approval DD/MM/YY**	**Am. 3 Approval DD/MM/YY**
1601 / Pier Luigi Paggiaro	COMITATO PER LA SPERIMENTAZIONE CLINICA DEI MEDICINALI DELL´AZIENDA OSPEDALIERO UNIVERSITARIA PISANA DI PISA VIA ROMA, 67 56126 PISA	Romano Danesi	14/03/2002	17/05/2002	25/07/2003	09/03/2006	13/09/2007
1602 / Carlo Cattaneo	COMITATO ETICO INDIPENDENTE DELL´AZIENDA OSPEDALIERA DELLA PROVINCIA DI PAVIA Viale Repubblica n. 34 27100 PAVIA	Alessandra Marinoni	14/03/2002	23/07/2002	23/09/2003	20/04/2006	25/09/2007
1603 / Marco Dottorini	COMITATO ETICO DELLE AZIENDE SANITARIE DELL´UMBRIA DI PERUGIA via della Rivoluzione, 16, 06070 Ellera di Corciano (PG)	Adolfo Puxeddu	14/03/2002	18/07/2002	11/09/2003	28/02/2006	13/09/2007
1604 / Maurizio Dottorini	COMITATO ETICO DELLE AZIENDE SANITARIE DELL´UMBRIA DI PERUGIA via della Rivoluzione,	Adolfo Puxeddu	14/03/2002	18/07/2002	11/09/2003	28/02/2006	13/09/2007

Italy							
**Centre number(s) / Investigator(s)**	**IRB or IEC (name/address)**	**IRB or IEC Chairperson**	**Informed Consent/Pt. Information Form Approval DD/MM/YY**	**Protocol Approval DD/MM/YY**	**Am. 1 Approval DD/MM/YY**	**Am. 2 Approval DD/MM/YY**	**Am. 3 Approval DD/MM/YY**
	16, 06070 Ellera di Corciano (PG)						
1606 / Roberto Tazza	COMITATO ETICO DELLE AZIENDE SANITARIE DELL´UMBRIA DI PERUGIA via della Rivoluzione, 16, 06070 Ellera di Corciano (PG)	Adolfo Puxeddu	14/03/2002	18/07/2002	11/09/2003	28/02/2006	13/09/2007
1607 / Prof. Pietro Pirina	COMITATO DI BIOETICA DELLA ASL DI SASSARI VIA MONTE GRAPPA, 82 07100 SASSARI	Guido Fellin	14/03/2002	10/07/2002	23/09/2003	12/09/2006	17/09/2007
1608 / Riccardo Ortu	COMITATO DI BIOETICA DELLA ASL DI SASSARI VIA MONTE GRAPPA, 82 07100 SASSARI	Guido Fellin	14/03/2002	10/07/2002	23/09/2003	12/09/2006	17/09/2007
1612 / Mario Sugamiele	AZIENDA OSPEDALIERA S.ANTONIO ABATE - TRAPANI via cosenza 82 91016 ERICE (TP)	Vincenzo Lima	14/03/2002	10/07/2002	16/10/2003	06/04/2006	14/12/2007
1613 / Michele Mastroberardino	CE c/o Ufficio Amministrativo A.O. “San Giuseppe	Fernando Salerno	14/03/2002	20/08/2002	19/12/2003	29/09/2006	27/09/2007

Italy							
**Centre number(s) / Investigator(s)**	**IRB or IEC (name/address)**	**IRB or IEC Chairperson**	**Informed Consent/Pt. Information Form Approval DD/MM/YY**	**Protocol Approval DD/MM/YY**	**Am. 1 Approval DD/MM/YY**	**Am. 2 Approval DD/MM/YY**	**Am. 3 Approval DD/MM/YY**
	Moscati"<br>Contrada Amoretta<br>Città Ospedaliera<br>83100 AVELLINO						
1614 / Giuseppe Anzalone	COMITATO ETICO ANCHE PER LA VALUTAZIONE E CONTROLLO DELLE SPERIMENTAZIONI CLINICHE DEI MEDICINALI DELLA AUSL 4 DI PRATO<br>V.le delle Repubblica 240, 59100 PRATO	Fabrizio Ledda	14/03/2002	01/07/2002	30/10/2003	10/04/2006	17/09/2007
1615 / Giorgio Walter Canonica	COMITATO ETICO DELL´AZIENDA OSPEDALIERA UNIVERSITARIA S. MARTINO DI GENOVA<br>Largo Rosanna Benzi 10, 16132 GENOVA	Luigi Francesco Meloni	14/03/2002	11/07/2002	29/09/2003	19/05/2006	26/10/2007
1617 / Dr. Fernando De Benedetto	COMITATO ETICO<br>Università degli Studi "G. D'Annunzio"<br>c/o Presidenza Facoltà di Medicina<br>Via dei Vestini, 31<br>66013 CHIETI	Mauro Carabella	14/03/2002	30/07/2002	23/09/2003	02/05/2006	25/09/2007

Italy							
**Centre number(s) / Investigator(s)**	**IRB or IEC (name/address)**	**IRB or IEC Chairperson**	**Informed Consent/Pt. Information Form Approval DD/MM/YY**	**Protocol Approval DD/MM/YY**	**Am. 1 Approval DD/MM/YY**	**Am. 2 Approval DD/MM/YY**	**Am. 3 Approval DD/MM/YY**
1618 / Franco Falcone	COMITATO ETICO c/o Dipartimento Farmaceutico Azienda USL di Bologna Via Gramsci, 12 40121 BOLOGNA	Lucia Alberghini	14/03/2002	18/07/2002	11/12/2003	22/03/2006	18/12/2007
1619 / Pietro Greco	COMITATO ETICO DELLA ASL DI CARBONIA (CI) Via Dalmazia 83 09013 CARBONIA (CI)	De Gregorio	14/03/2002	09/07/2002	30/09/2003	28/03/2006	29/10/2007
1622 / Adalberto Ciaccia	Comitato Etico della Provincia di Ferrara Corso Giovecca, 203 44100 FERRARA	Voci / Palara	14/03/2002	23/07/2002	07/10/203	12/04/2006	27/09/2007
1623 / Rigoletta Vincenti	Comitato Etico ASL 1 di Massa e Carrara Via Porta Parma 54027 PONTREMOLI (MS)	Margherita Rinaldi	14/03/2002	06/07/2002	25/09/2003	02/03/2006	07/08/2007
1624 / Alberto Tubaldi	COMITATO ETICO INTERZONALE DELLA ASUR ZONA TERRITORIALE 8 DI CIVITANOVA MARCHE E ZONA TERRITORIALE 9 DI MACERATA VIA SANTA LUCIA 2 62100 MACERATA	Americo Sbriccoli	14/03/2002	26/06/2002	25/09/2003	24/08/2006	approval not needed: the ec will provide a note to take cognizance of am. 3

Italy							
**Centre number(s) / Investigator(s)**	**IRB or IEC (name/address)**	**IRB or IEC Chairperson**	**Informed Consent/Pt. Information Form Approval DD/MM/YY**	**Protocol Approval DD/MM/YY**	**Am. 1 Approval DD/MM/YY**	**Am. 2 Approval DD/MM/YY**	**Am. 3 Approval DD/MM/YY**
1629 / Virginia De Rose	COMITATO ETICO DELL´AZIENDA OSPEDALIERA UNIVERSITARIA S. LUIGI GONZAGA DI ORBASSANO REGIONE GONZOLE 10 10043 ORBASSANO	Francesco Di Carlo	14/03/2002	09/09/2002	08/09/2003	29/05/2006	01/10/2007
1631 / Gabriele Ferretti	COMITATO ETICO DELL´AZIENDA OSPEDALIERA SS. ANTONIO E BIAGIO E CESARE ARRIGO DI ALESSANDRIA VIA VENEZIA 16, 15100, ALESSANDRIA	Ilario Viano	14/03/2002	09/09/2002	29/09/2003	21/02/2006	18/09/2007
1632 / Gianni Forconi	COMITATO ETICO DELLA ASL TO/2 DI TORINO STRADA DELL'ARRIVORE 25/A, 10154 TORINO	Maria Chiara Cassone	14/03/2002	28/10/2002	NA	NA	NA
1633 / Stefano Gasparini	COMITATO ETICO DELL´AZIENDA OSPEDALIERO-UNIVERSITARIA OSPEDALI RIUNITI UMBERTO I - G.M. LANCISI - G. SALESI DI ANCONA	Tullio Manzoni	14/03/2002	03/10/2002	27/11/2003	NA	NA

Italy							
**Centre number(s) / Investigator(s)**	**IRB or IEC (name/address)**	**IRB or IEC Chairperson**	**Informed Consent/Pt. Information Form Approval DD/MM/YY**	**Protocol Approval DD/MM/YY**	**Am. 1 Approval DD/MM/YY**	**Am. 2 Approval DD/MM/YY**	**Am. 3 Approval DD/MM/YY**
	VIA CONCA 71<br>60126 ANCONA						
1634 / Delfino Legnani	COMITATO ETICO LOCALE PER LA SPERIMENTAZIONE CLINICA DELL'AZIENDA OSPEDALIERA LUIGI SACCO DI MILANO<br>VIA G.B. GRASSI, 74<br>20157 MILANO	Emilio Tracbucchi	14/03/2002	18/09/2002	17/09/2003	29/03/2006	19/09/2007
1635 / Giovanni Paolo Ligia	COMITATO ETICO DELLA ASL DI CAGLIARI<br>VIA LOGUDORO 17<br>09127 CAGLIARI	Luigi Minerba	14/03/2002	18/09/2002	22/10/2003	24/05/2006	24/10/2007
1639 / Vincenzo Padua	COMITATO ETICO DELLA AUSL 7 DI RAGUSA<br>Piazza Igea 2<br>97100 RAGUSA	Pietro Bonomo	14/03/2002	25/09/2002	23/09/2003	26/09/2006	10/09/2007
1640 / Giorgio Santelli	COMITATO ETICO PER LA SPERIMENTAZIONE CLINICA DELLA PROVINCIA DI TREVISO<br>BORGO CAVALLI 42<br>31100 TREVISO	Paolo Tottolo	14/03/2002	25/09/2002	29/10/2003	24/05/2006	20/09/2007

Italy							
**Centre number(s) / Investigator(s)**	**IRB or IEC (name/address)**	**IRB or IEC Chairperson**	**Informed Consent/Pt. Information Form Approval DD/MM/YY**	**Protocol Approval DD/MM/YY**	**Am. 1 Approval DD/MM/YY**	**Am. 2 Approval DD/MM/YY**	**Am. 3 Approval DD/MM/YY**
1643 / Francesco Mazza	COMITATO ETICO INDIPENDENTE PER LA VALUTAZIONE DELLE SPERIMENTAZIONI CLINICHE DEI MEDICINALI DELL´AZIENDA OSPEDALIERA S. MARIA DEGLI ANGELI DI PORDENONE via montereale, 24 33170 PORDENONE	-----	14/03/2002	13/09/2002	14/04/2004	30/05/2006	30-31/10/2007
1645 / Roberto Cogo	COMITATO ETICO DELL´AZIENDA OSPEDALIERA DI MELEGNANO (MI) VIA PANDINA 1 20070 VIZZOLO PREDABISSI (MI)	Roberto Cosentina	14/03/2002	22/10/2002	24/02/2004	NA	NA
1646 / Andrea Lopes Pegna	COMITATO ETICO PER LA SPERIMENTAZIONE CLINICA DEI MEDICINALI DELL´AZIENDA OSPEDALIERO-UNIVERSITARIA CAREGGI DI	Giovanni Passagnoli	14/03/2002	23/09/02	15/09/2003	07/03/2006	03/10/2007

Italy							
**Centre number(s) / Investigator(s)**	**IRB or IEC (name/address)**	**IRB or IEC Chairperson**	**Informed Consent/Pt. Information Form Approval DD/MM/YY**	**Protocol Approval DD/MM/YY**	**Am. 1 Approval DD/MM/YY**	**Am. 2 Approval DD/MM/YY**	**Am. 3 Approval DD/MM/YY**
	FIRENZE viale Pieraccini, 28 50139 FIRENZE						
1647 / Massimo Pistolesi	COMITATO ETICO PER LA SPERIMENTAZIONE CLINICA DEI MEDICINALI DELL´AZIENDA OSPEDALIERO-UNIVERSITARIA CAREGGI DI FIRENZE viale Pieraccini, 28 50139 FIRENZE	Giovanni Passagnoli	14/03/2002	23/09/02	15/09/2003	07/03/2006	03/10/2007
1648 / Vincenzo Colorizio	COMITATO ETICO DELLA AUSL 4 DI L'AQUILA Azienda U.S.L, 4 Comitato Etico ex P.O. Santa Maria di Collemaggio 67100 L'AQUILA	Guiseppe Cerone	14/03/2002	13/08/02	24/09/03	23/03/06	13/09/07
1649 / Dr. Pietro Zanon	COMITATO ETICO DELL´AZIENDA OSPEDALIERA OSPEDALE DI CIRCOLO DI BUSTO ARSIZIO (VA) PIAZZALE SOLARO, 3 21052 BUSTO	Emanuela Cerruti	14/03/2002	04/10/02	12/12/03	27/10/06	19/10/07

Italy							
**Centre number(s) / Investigator(s)**	**IRB or IEC (name/address)**	**IRB or IEC Chairperson**	**Informed Consent/Pt. Information Form Approval DD/MM/YY**	**Protocol Approval DD/MM/YY**	**Am. 1 Approval DD/MM/YY**	**Am. 2 Approval DD/MM/YY**	**Am. 3 Approval DD/MM/YY**
	ARSIZIO (VA)						

Japan							
**Centre number(s) / Investigator(s)**	**IRB or IEC (name/address)**	**IRB or IEC Chairperson**	**Informed Consent/Pt. Information Form Approval DD/MM/YY**	**Protocol Approval DD/MM/YY**	**Am. 1 Approval DD/MM/YY**	**Am. 2 Approval DD/MM/YY**	**Am. 3 Approval DD/MM/YY**
1701 / Masaharu Nishimura	The IRB of Hokkaido University / Nishi 5, Kita Juyonjou, Kita-ku, Sapporo, Hokkaido, 060-8648	Katsumi Miyazaki (12 September 2002) Ken Iseki (7 March 2006)	12/09/02	12/09/02	05/08/03	07/03/06	05/06/07
1702 / Kohei Yamauchi	The IRB of Iwate Medical University / 19-1 Uchimaru, Morioka, Iwate, 020-8505	Hiroshi Inoue (18 September 2002) Kazuyuki Suzuki (16 March 2006)	18/09/02	18/09/02	04/08/03	16/03/06	30/05/07
1703 / Hiromasa Ogawa	The IRB of Tohoku University / 1-1 Seiryo-cho, Aoba-ku, Sendai, Miyagi, 980-8574	Kazuhiko Taniuchi (2 September 2002 / 1 September 2003/ 1 March 2006) Toshio Hattori (2 July 2007)	02/09/02	02/09/02	01/09/03	01/03/06	02/07/07
1704 / Keiji Kimura	The IRB of Hiraka General Hospital / 3-1, Aza Yatsukuchi, Maegou, Yokote, Akita, 013-8610	Toshiharu Okubo (20 January 2003 / 19 August 2003) Katsu Hirayama (6 February 2006/ 6 August 2007)	20/01/03	20/01/03	19/08/03	06/02/06	06/08/07
1705 / Norihiro Kaneko	The IRB of Kameda Medical Center / 929 Higashimachi, Kamogawa, Chiba, 296-0041	Yuji Hashimoto	04/09/02	04/09/02	07/08/03	08/02/06	04/07/07
1706 / Kuniaki Seyama	The IRB of Juntendo University / 3-1-3 Hongo, Bunkyo-ku, Tokyo, 113-8431	Ryuzo Kawamori	08/10/02	08/10/02	18/09/03	14/02/06	12/07/07

Japan							
**Centre number(s) / Investigator(s)**	**IRB or IEC (name/address)**	**IRB or IEC Chairperson**	**Informed Consent/Pt. Information Form Approval DD/MM/YY**	**Protocol Approval DD/MM/YY**	**Am. 1 Approval DD/MM/YY**	**Am. 2 Approval DD/MM/YY**	**Am. 3 Approval DD/MM/YY**
1708 / Keishi Kubo	The IRB of Shinshu University / 3-1-1 Asahi Matsumoto, Nagano, 390-0802	Shigeru Omori (22 October 2002); Toshiaki Saida (15 February 2006/ 19 June 2007)	22/10/02	22/10/02	10/07/03	15/02/06	19/06/07
1709 / Hiroyuki Taniguchi	The IRB of Tosei General Hospital / 160 Nishi-Oiwake-cho, Seto, Aichi, 489-0065	Kazuyoshi Sakai	25/07/02	25/07/02	25/07/03	27/01/06	30/07/07
1710 / Kazuto Hirata	The IRB of Osaka City University / 1-5-7 Asahimachi, Abeno-ku, Osaka, Osaka, 545-8586	Masamitsu Ishii (29 January 2003/ 24 September 2003 22 February 2006); Kunio Shiraki (27 June 2007)	29/01/03	29/01/03	24/09/03	22/02/06	27/06/07
1711 / Yuji Tohda	The IRB of Kinki University / 377-2 Ohno-higashi, Osakasayama, Osaka, 589-0014	Harumasa Oyangai (29 July 2002) Masahiro Fukuoka (20 February 2006) Susumu Kusunoki (18 June 2007)	29/07/02	29/07/02	11/08/03	20/02/06	18/06/07
1712 / Hisamichi Aizawa	The IRB of Kurume University Hospital / 67 Asahi-machi, Kurume, Fukuoka, 830-0011	Takashi Hashimoto	21/10/02	21/10/02	18/08/03	20/08/06	18/06/07
1713 / Shuichi Matsumoto	The IRB of Komaki City Hospital / 1-20 Joubushi, Komaki, Aichi, 485-0044	Tatsuya Kobayashi (12 November 2002) Tsukasa Imaizumi (17 September 2003) Yoshihisa Kida (8 June 2006)	12/11/02	12/11/02	17/09/03	08/06/06	23/05/07

\

Lithuania							
1.Date of IC/PI - version 1 dated 14 February, 2002 was approved on 10-Jun-2002 - date of approval to conduct biomedical research.<br>IC/PI version 2 dated 9 November, 2004 was approved on 03-Mar-05 - this date entered into table<br><br>2.<br>Amendment 1 - was only submitted as information(issue not related to Lithuania)<br>Amendment 2 - approved<br>Amendment 3 submitted on 09-May-07. Lithuanian Bioethics Committee this information accepted, approval not needed as there were no changes in IC or PI.							
**Centre number(s) / Investigator(s)**	**IRB or IEC (name/address)**	**IRB or IEC Chairperson**	**Informed Consent/Pt. Information Form Approval DD/MM/YY**	**Protocol Approval DD/MM/YY**	**Am. 1 Approval DD/MM/YY**	**Am. 2 Approval DD/MM/YY**	**Am. 3 Approval DD/MM/YY**
1801 / Raimundas Sakalauskas	Lithuanian Bioethics Committee Didzioji str. 22, LT 01128, Vilnius	Assoc.prof. E.Gefenas	03-Mar-05	28-May-02	Not for LT	30-Jan-06	N/A
1802 / Alfredas Bagdouas	Lithuanian Bioethics Committee Didzioji str. 22, LT 01128, Vilnius	Assoc.prof. E.Gefenas	03-Mar-05	28-May-02	Not for LT	30-Jan-06	N/A
1803 / Remigijus V. Nargela	Lithuanian Bioethics Committee Didzioji str. 22, LT 01128, Vilnius	Assoc.prof. E.Gefenas	03-Mar-05	28-May-02	Not for LT	30-Jan-06	N/A

Malaysia							
**Centre number(s) / Investigator(s)**	**IRB or IEC (name/address)**	**IRB or IEC Chairperson**	**Informed Consent/Pt. Information Form Approval DD/MM/YY**	**Protocol Approval DD/MM/YY**	**Am. 1 Approval DD/MM/YY**	**Am. 2 Approval DD/MM/YY**	**Am. 3 Approval DD/MM/YY**
1901 / Aziah Ahmad Mahiyidin	Medical Research Ethics Committee Ministry of Health C/o Institute for Medical Research Jalan Pahang 50588 Kuala Lumpur Malaysia	Mr Noor Hisham Abdullah	23 Oct 2002	23 Oct 2002	28 Aug 2006	28 Aug 2006	24 May 2007
1902 / Roslan Harun	Research Ethics Committee Faculty of Medicine University Kebangsaan Malaysia Jalan Yaacob Latif 56000 Cheras Kuala Lumpur	Prof. Dr. Raymond Azman Ali	02 Dec 2002	02 Dec 2002	22 Aug 2006	22 Aug 2006	11 Jul 2007
1903 / Liam Chong Kim	Medical Ethics Committee University of Malaya Medical Centre Lembah Pantai 59100 Kuala Lumpur Malaysia	Prof Looi Lai Meng	25 Sep 2002	25 Sep 2002	16 Aug 2006	16 Aug 2006	25 Jul 2007

Mexico							
**Centre number(s) / Investigator(s)**	**IRB or IEC (name/address)**	**IRB or IEC Chairperson**	**Informed Consent/Pt. Information Form Approval DD/MM/YY**	**Protocol Approval DD/MM/YY**	**Am. 1 Approval DD/MM/YY**	**Am. 2 Approval DD/MM/YY**	**Am. 3 Approval DD/MM/YY**
2002 / Raúl Ortiz Peregrina	IRB del Hospital Civil Nuevo de Guadalajara Salvador Quevedo y Zubieta # 750 Col Independencia ZC 44340 Guadalajara, Jal	Salvador Fonseca M.D.	17-May-2002	17-May-2002	NA	05-Apr-2006	11- Jul-2007
2003 / Javier Díaz Castañón	IRB del Hospital Angel Leaño Av Dr Angel Leaño # 500Col. Los Robles ZC 45200 Zapopan, Jal	Victoriano Sáenz Félix, M.D.<br><br>Actual: Bruna Isabella Taboada Mascarin M.D.	12-Jun-2002	12-Jun-2002	NA	28-Feb-2006	25-Jun-07
2004 / Rodolfo Posadas Valay	IRB/IEC del Hospital Universitario de UANL Av Francisco I Madero Pte s/n y Av Gonzalitos Col. Mitras ZC 64460 Monterrey Nuevo León	José Garza Leal, M.D.	10-Oct-2002	10-Oct-2002	NA	09-Mar-2006	01-Jun-2007
2006 / Raúl Sansores	IRB INER Calzada de Tlalpan # 4502 Col Sección XVI ZC14080 Mexico City, México	Guillermo Carvajal Sandoval, M.D.	02-Jul-2002	02-Jul-2002	NA	22-Mar-2006	06-Jun-2007

Mexico							
**Centre number(s) / Investigator(s)**	**IRB or IEC (name/address)**	**IRB or IEC Chairperson**	**Informed Consent/Pt. Information Form Approval DD/MM/YY**	**Protocol Approval DD/MM/YY**	**Am. 1 Approval DD/MM/YY**	**Am. 2 Approval DD/MM/YY**	**Am. 3 Approval DD/MM/YY**
2009 / Alfredo Domínguez Peregrina	IRB Hospital UPAEP 5 Poniente #175 col Centro ZC 72000 Puebla, Pueb	Lorena de la Calleja, M.D.	01-Jul-2002	01-Jul-2002	NA	18-May-2006	16-Jun-2007
2010 / Héctor Ocaña Servín	IRB de la Universidad Autónoma del Estado de México Ixtacihuatl # 297 Col. Xuiautecal Toluca, Edo de México	José María Pérez Aviles, M.D.	14-Aug-2002	14-Aug-2002	NA	ND	ND

Netherlands							
**Centre number(s) / Investigator(s)**	**IRB or IEC (name/address)**	**IRB or IEC Chairperson**	**Informed Consent/Pt. Information Form Approval DD/MM/YY**	**Protocol Approval DD/MM/YY**	**Am. 1 Approval DD/MM/YY**	**Am. 2 Approval DD/MM/YY**	**Am. 3 Approval DD/MM/YY**
Central approval for all sites (2101-2115)	Medisch Ethische Toetsingscommissie Atrium MC – Maaslandziekenhuis<br>Henri Dunantstraat 5<br>6419 PC HEERLEN	G. Blaauw	same	08-Aug-02	29-Jul-03	25-Jan-06	29-May-07
2101 / Jan van Noord	Raad van Bestuur Atrium medisch centrum<br>Henri Dunantstraat 5<br>6419 PC HEERLEN	Dr. Ir. J.W.J. van Wersch	same	15-Oct-02	Not Applicable*	Not Applicable*	Not Applicable*
2102 / R. Aalbers	Medisch Ethische Toetsingscommissie Atrium MC – Maaslandziekenhuis<br>Henri Dunantstraat 5<br>6419 PC HEERLEN	G. Blaauw	same	04-Sep-02	Not Applicable*	Not Applicable*	Not Applicable*
2104 / J.P.H.M. Creemers	Medisch Ethische Toetsingscommissie Atrium MC – Maaslandziekenhuis<br>Henri Dunantstraat 5<br>6419 PC HEERLEN	G. Blaauw	same	08-Aug-02	Not Applicable*	Not Applicable*	Not Applicable*
2105 / W. Dalinghaus	Medisch Ethische Toetsingscommissie Atrium MC – Maaslandziekenhuis<br>Henri Dunantstraat 5<br>6419 PC HEERLEN	G. Blaauw	same	17-Oct-02	Not Applicable*	Not Applicable*	Not Applicable*
2106 / M. Eland	Medisch Ethische Toetsingscommissie Atrium MC – Maaslandziekenhuis<br>Henri Dunantstraat 5<br>6419 PC HEERLEN	G. Blaauw	same	17-Oct-02	Not Applicable*	Not Applicable*	Not Applicable*
2107 / B.J.M. Pannekoek	Medisch Ethische Toetsingscommissie Atrium MC – Maaslandziekenhuis	G. Blaauw	same	08-Aug-02	Not Applicable*	Not Applicable*	Not Applicable*

Netherlands							
Centre number(s) / Investigator(s)	IRB or IEC (name/address)	IRB or IEC Chairperson	Informed Consent/Pt. Information Form Approval DD/MM/YY	Protocol Approval DD/MM/YY	Am. 1 Approval DD/MM/YY	Am. 2 Approval DD/MM/YY	Am. 3 Approval DD/MM/YY
	Henri Dunantstraat 5<br>6419 PC HEERLEN						
2108 / H.R. Pasma	Medisch Ethische Toetsingscommissie Atrium MC – Maaslandziekenhuis<br>Henri Dunantstraat 5<br>6419 PC HEERLEN	G. Blaauw	same	08-Aug-02	Not Applicable*	Not Applicable*	Not Applicable*
2109 / A. Rudolphus	Medisch Ethische Toetsingscommissie Atrium MC – Maaslandziekenhuis<br>Henri Dunantstraat 5<br>6419 PC HEERLEN	G. Blaauw	same	18-Dec-02	Not Applicable*	Not Applicable*	Not Applicable*
2110 / H.E.J. Sinn. Damsté	Medisch Ethische Toetsingscommissie Atrium MC – Maaslandziekenhuis<br>Henri Dunantstraat 5<br>6419 PC HEERLEN	G. Blaauw	same	20-Jan-03	Not Applicable*	Not Applicable*	Not Applicable*
2111/ W.B.M. Evers	Medisch Ethische Toetsingscommissie Atrium MC – Maaslandziekenhuis<br>Henri Dunantstraat 5<br>6419 PC HEERLEN	G. Blaauw	same	08-Aug-02	Not Applicable*	Not Applicable*	Not Applicable*
2112 / S.J.M. Gans	Medisch Ethische Toetsingscommissie Atrium MC – Maaslandziekenhuis<br>Henri Dunantstraat 5<br>6419 PC HEERLEN	G. Blaauw	same	17-Oct-02	Not Applicable*	Not Applicable*	Not Applicable*
2114 / Hans Timmer	Medisch Ethische Toetsingscommissie Atrium MC – Maaslandziekenhuis<br>Henri Dunantstraat 5	G. Blaauw	same	09-Jan-03	Not Applicable*	Not Applicable*	Not Applicable*

Netherlands							
**Centre number(s) / Investigator(s)**	**IRB or IEC (name/address)**	**IRB or IEC Chairperson**	**Informed Consent/Pt. Information Form Approval DD/MM/YY**	**Protocol Approval DD/MM/YY**	**Am. 1 Approval DD/MM/YY**	**Am. 2 Approval DD/MM/YY**	**Am. 3 Approval DD/MM/YY**
	6419 PC HEERLEN						
2115 / J. Westbroek	Medisch Ethische Toetsingscommissie Atrium MC – Maaslandziekenhuis<br>Henri Dunantstraat 5<br>6419 PC HEERLEN	G. Blaauw	same	17-Oct-02	Not Applicable*	Not Applicable*	Not Applicable*

New Zealand							
Centre number(s) / Investigator(s)	IRB or IEC (name/address)	IRB or IEC Chairperson	Informed Consent/Pt. Information Form Approval DD/MM/YY	Protocol Approval DD/MM/YY	Am. 1 Approval DD/MM/YY	Am. 2 Approval DD/MM/YY	Am. 3 Approval DD/MM/YY
211 / Peter Black	Multi-Region Ethics Committee	Nicole Presland	16Jan2003	16Jan2003	30Jul2003	08Feb2006	30May2007
212 / Michael Epton	Multi-Region Ethics Committee	Nicole Presland	16Jan2003	16Jan2003	30Jul2003	08Feb2006	30May2007

Norway							
**Centre number(s) / Investigator(s)**	**IRB or IEC (name/address)**	**IRB or IEC Chairperson**	**Informed Consent/Pt. Information Form Approval DD/MM/YY**	**Protocol Approval DD/MM/YY**	**Am. 1 Approval DD/MM/YY**	**Am. 2 Approval DD/MM/YY**	**Am. 3 Approval DD/MM/YY**
2201 / Ragnar Dahle	Regional komite for medisinsk forskningsetikk Postboks 1130, Blindern 0318 OSLO	Knut Engedal	10/09/02	10/09/02	NA	19/12/05	No approval only send as information to IRB
2202 / Arne Eivindson	Regional komite for medisinsk forskningsetikk Postboks 1130, Blindern 0318 OSLO	Knut Engedal	10/09/02	10/09/02	NA	19/12/05	No approval only send as information to IRB
2204 / Jostein Asmervik	Regional komite for medisinsk forskningsetikk Postboks 1130, Blindern 0318 OSLO	Knut Engedal	10/09/02	10/09/02	NA	19/12/05	No approval only send as information to IRB
2205 / Per Arve Lier	Regional komite for medisinsk forskningsetikk Postboks 1130, Blindern 0318 OSLO	Knut Engedal	10/09/02	10/09/02	NA	19/12/05	No approval only send as information to IRB

Philippines							
**Centre number(s) / Investigator(s)**	**IRB or IEC (name/address)**	**IRB or IEC Chairperson**	**Informed Consent/Pt. Information Form Approval DD/MM/YY**	**Protocol Approval DD/MM/YY**	**Am. 1 Approval DD/MM/YY (FYI only)**	**Am. 2 Approval DD/MM/YY**	**Am. 3 Approval DD/MM/YY**
2301 / Lenora Canizares-Fernandez	Research Implementation and Development Office UP-College of Medicine Philippine General Hospital Taft Avenue, Ermita Manila	Evangeline O. Santos, MD	16-Jan-03	16-Jan-03	24-Jul-03	08-Mar-06	11-May-07
2302 / Dr. Aileen Guzman	Ethics Committee Philippine Heart Center East Avenue, Quezon City	Noe A. Babilonia, MD	18-Jul-02	18-Jul-02	23-Jul-03	09-Mar-06	04-May-07
2303 / Teresita Aquino	Research and Ethics Committee Veterans Memorial medical Center North Avenue, Quezon City	Emerita A. Barrenechea, MD	17-Jun-02	17-Jun-02	23-Jul-03	08-Mar-06	10-May-07
2304 / Sullian Sy-Naval	Ethics Review Committee Lung Center of the Philippines East Avenue, Quezon City	Fr. Efren B. Ballistoy, OSC	26-Jun-02	26-Jun-02	01-Sep-03	08-Mar-06	15-May-07
2305 / Tim Trinidad	Institutional Review Board University of Santo Tomas Hospital España, Manila	Ma. Graciela Garayblas-Gonzaga, MD	12-Feb-02	12-Feb-02	05-Jan-04	08-Mar-06	07-May-07

Poland							
In section Informed Consent approval I gave a comment that it applies to PIC version 2 dated 09.11.2004 as PIC version 1 dated 14.02.2002 was approved together with main protocol approval - I also added that comment in the section.<br>The last comment is required for Am 2 approval - that date applies also to the PIC for long term follow-up approval, which was also applicable in Poland.<br>The site 2403 was closed soon after the opening so that they have only main protocol approval.							
**Centre number(s) / Investigator(s)**	**IRB or IEC (name/address)**	**IRB or IEC Chairperson**	**Informed Consent/Pt. Information Form Approval DD/MM/YY**	**Protocol Approval DD/MM/YY**	**Am. 1 Approval DD/MM/YY**	**Am. 2 Approval DD/MM/YY**	**Am. 3 Approval DD/MM/YY**
2401 / Prof. Dorota Górecka	Komisja Bioetyczna Przy Instytucie Gruźlicy i Chorób Płuc<br>Ul. Płocka 26<br>01-138 Warszawa	Prof. Dr hab. JAN KUŚ	20/06/05	02/10/02	25/09/03	16/01/06	20/06/07
2402 / Tadeusz Plusa	Komisja Bioetyczna Przy Wojskowej Izbie Lekarskiej<br>Ul. Koszykowa 78<br>00-909 Warszawa	1. Prof. Dr hab. DARIUSZ JURKIEWICZ<br>2.Prof. Dr hab. EUGENIUSZ DZIUK	24/05/05	18/09/02	22/10/03	27/01/06	15/06/07
2403 / Michal Pirozunski	Komisja Bioetyczna CMKP Ul. Marymoncka 99, Warszawa	Prof. Dr hab. EWA MARCINOWSKA-SUCHOWIERSKA	Site closed – no patient	04/09/02	NA	NA	NA
2404 / Prof. Ewa Pisarczyk-Bogacka	Komisja Bioetyczna Przy Akademii Medycznej<br>Ul. Pasteura 1<br>50-367 Wrocław	1. Prof. Dr hab. FRANCISZEK IWAŃCZAK<br>2. Prof. Dr hab. JAN KORNAFEL	09/06/05	10/10/02	17/10/03	12/01/06	29/06/07
2405 / Pawel Gorski	Komisja Bioetyki Uniwersytetu Medycznego w Łodzi<br>Al. Kościuszki 4<br>90-419 Łódź	Prof. Dr hab. PRZEDZISŁAW POLAKOWSKI	14/06/05	07/10/02	23/09/03	17/01/06	26/06/07

Poland							
In section Informed Consent approval I gave a comment that it applies to PIC version 2 dated 09.11.2004 as PIC version 1 dated 14.02.2002 was approved together with main protocol approval - I also added that comment in the section.<br>The last comment is required for Am 2 approval - that date applies also to the PIC for long term follow-up approval, which was also applicable in Poland.<br>The site 2403 was closed soon after the opening so that they have only main protocol approval.							
**Centre number(s) / Investigator(s)**	**IRB or IEC (name/address)**	**IRB or IEC Chairperson**	**Informed Consent/Pt. Information Form Approval DD/MM/YY**	**Protocol Approval DD/MM/YY**	**Am. 1 Approval DD/MM/YY**	**Am. 2 Approval DD/MM/YY**	**Am. 3 Approval DD/MM/YY**
2406 / Jerzy Kozielski	Komisja Bioetyczna Kraków, 23.01.2008 Przy Śląskiej Akademii Medycznej Ul. Warszawska 14 40-006 Katowice	Prof. Dr hab. STEFAN KOSSMANN	08/06/05	25/09/02	20/11/03	26/01/06	20/06/07
2407 / Wladyslaw Pierzchala	Komisja Bioetyczna Kraków, 23.01.2008 Przy Śląskiej Akademii Medycznej Ul. Warszawska 14 40-006 Katowice	Prof. Dr hab. STEFAN KOSSMANN	08/06/05	25/09/02	20/11/03	24/01/06	20/06/07
2408 / Andrzej Szczeklik	Komisja Bioetyczna Kraków, 23.01.2008 Uniwersytetu Jagiellońskiego Ul. Jagiellońska 10 31-010 Kraków	Prof. Dr hab. PIOTR THOR	23/06/05	24/10/02	25/09/03	12/01/06	28/06/07

Poland							
In section Informed Consent approval I gave a comment that it applies to PIC version 2 dated 09.11.2004 as PIC version 1 dated 14.02.2002 was approved together with main protocol approval - I also added that comment in the section.<br>The last comment is required for Am 2 approval - that date applies also to the PIC for long term follow-up approval, which was also applicable in Poland.<br>The site 2403 was closed soon after the opening so that they have only main protocol approval.							
**Centre number(s) / Investigator(s)**	**IRB or IEC (name/address)**	**IRB or IEC Chairperson**	**Informed Consent/Pt. Information Form Approval DD/MM/YY**	**Protocol Approval DD/MM/YY**	**Am. 1 Approval DD/MM/YY**	**Am. 2 Approval DD/MM/YY**	**Am. 3 Approval DD/MM/YY**
2409 / Piotr Kuna	Komisja Bioetyki Uniwersytetu Medycznego w Łodzi Al. Kościuszki 4 90-419 Łódź	Prof. Dr hab. PRZEDZISŁAW POLAKOWSKI	14/06/05	03/09/02	23/09/03	17/01/06	26/06/07
2410 / Jan Marek Slominski	Niezależna Komisja Bioetyczna, Kraków, 23.01.2008 d/s Badań Naukowych Przy Akademii Madycznej w Gdańsku Ul. Marii Skłodowskiej-Curie 3A 80-210 Gdańsk	Prof. Dr hab. STEFAN RASZEJA	30/06/05	19/09/02	03/10/03	14/02/06	09/07/07

Portugal							
**Centre number(s) / Investigator(s)**	**IRB or IEC (name/address)**	**IRB or IEC Chairperson**	**Informed Consent/Pt. Information Form Approval DD/MM/YY**	**Protocol Approval DD/MM/YY**	**Am. 1 Approval DD/MM/YY ***	**Am. 2 Approval DD/MM/YY ****	**Am. 3 Approval DD/MM/YY ****
2501 / João Almeida	Comissão de Ética do Hospital de S. João Av. Prof. Hernâni Monteiro 4200 Porto	Dr. Filipe Nuno Almeida	22 APR 02	22 APR 02	16 SEP 03	16 FEB 06	22 MAY 07

Poland							
In section Informed Consent approval I gave a comment that it applies to PIC version 2 dated 09.11.2004 as PIC version 1 dated 14.02.2002 was approved together with main protocol approval - I also added that comment in the section.<br>The last comment is required for Am 2 approval - that date applies also to the PIC for long term follow-up approval, which was also applicable in Poland.<br>The site 2403 was closed soon after the opening so that they have only main protocol approval.							
**Centre number(s) / Investigator(s)**	**IRB or IEC (name/address)**	**IRB or IEC Chairperson**	**Informed Consent/Pt. Information Form Approval DD/MM/YY**	**Protocol Approval DD/MM/YY**	**Am. 1 Approval DD/MM/YY**	**Am. 2 Approval DD/MM/YY**	**Am. 3 Approval DD/MM/YY**
2502 / Dr. Raul Sa	Comissão de Ética do Centro Hospitalar de Vila Nova de Gaia<br>Rua Conceição Fernandes<br>4434-502 Vila Nova de Gaia	Dr. Eurico Teixeira	12 JUN 02	12 JUN 02	04 SEP 03	16 FEB 06	22 MAY 07
2503 / Cristina Bárbara	Comissão de Ética d0 Hospital de Pulido Valente<br>Alameda das Linhas de Torres, 117 - Edifício Rainha D. Amélia, 2º<br>1769-001 Lisboa	Dr. António D'Orey Franco	21 AUG 02	21 AUG 02	11 MAR 04	16 FEB 06	22 MAY 07
2504 / Dr. Joaquim Moita	Comissão de Ética do Centro Hospitalar de Coimbra<br>Quinta dos Vales – S. Martinho do Bispo<br>3040-853 Coimbra	Dr. João Sarabando Moreira	04 JUN 02	04 JUN 02	04 SEP 03	16 FEB 06	22 MAY 07
2505 / João Cardoso	Comissão de Ética do Hospital de Santa Marta<br>Rua de Santa Marta<br>1169-024 Lisboa	Prof. Dr. José Fragata	07 JUN 02	07 JUN 02	1 MAR 04	16 FEB 06	22 MAY 07
* Note: Dates presented are application dates, and not approval dates; approval was not required<br>** Amendment 2 and 3 were approved by a Central Ethic Committee; Name: Comissão de Ética para a Investigação Clínica; Address: Parque da Saúde de							

Poland							
In section Informed Consent approval I gave a comment that it applies to PIC version 2 dated 09.11.2004 as PIC version 1 dated 14.02.2002 was approved together with main protocol approval - I also added that comment in the section.<br>The last comment is required for Am 2 approval - that date applies also to the PIC for long term follow-up approval, which was also applicable in Poland.<br>The site 2403 was closed soon after the opening so that they have only main protocol approval.							
**Centre number(s) / Investigator(s)**	**IRB or IEC (name/address)**	**IRB or IEC Chairperson**	**Informed Consent/Pt. Information Form Approval DD/MM/YY**	**Protocol Approval DD/MM/YY**	**Am. 1 Approval DD/MM/YY**	**Am. 2 Approval DD/MM/YY**	**Am. 3 Approval DD/MM/YY**
Lisboa, Av. do Brasil, nº 53 – Pav. 17A, 1749-004 Lisboa; Chairperson: Dr. António Barros Veloso							

Russia							
**Centre number(s) / Investigator(s)**	**IRB or IEC (name/address)**	**IRB or IEC Chairperson**	**Informed Consent/Pt. Information Form Approval DD/MM/YY**	**Protocol Approval DD/MM/YY**	**Am. 1 Approval DD/MM/YY**	**Am. 2 Approval DD/MM/YY**	**Am. 3 Approval DD/MM/YY**
2601 / Alexander G. Chuchalin	Ethical Committee at the Federal Department of Drug Quality, Efficacy and Safety Control / 1 kor., 8, Petrovsky blvrd., 103051, Moscow, Russia	Prof. F.I.Komarov	21-05-02	24-09-03	26-12-05	05-06-07	02-07-07
2602 / Svetlana Ovtcharenko	Ethical Committee at the Federal Department of Drug Quality, Efficacy and Safety Control / 1 kor., 8, Petrovsky blvrd., 103051, Moscow, Russia	Prof. F.I.Komarov	21-05-02	24-09-03	26-12-05	05-06-07	29-05-07
2603 / Vladimir Nonikov	Ethical Committee at the Federal Department of Drug	Prof. F.I.Komarov	21-05-02	24-09-03	26-12-05	05-06-07	02-07-07

Poland							
In section Informed Consent approval I gave a comment that it applies to PIC version 2 dated 09.11.2004 as PIC version 1 dated 14.02.2002 was approved together with main protocol approval - I also added that comment in the section.<br>The last comment is required for Am 2 approval - that date applies also to the PIC for long term follow-up approval, which was also applicable in Poland.<br>The site 2403 was closed soon after the opening so that they have only main protocol approval.							
**Centre number(s) / Investigator(s)**	**IRB or IEC (name/address)**	**IRB or IEC Chairperson**	**Informed Consent/Pt. Information Form Approval DD/MM/YY**	**Protocol Approval DD/MM/YY**	**Am. 1 Approval DD/MM/YY**	**Am. 2 Approval DD/MM/YY**	**Am. 3 Approval DD/MM/YY**
	Quality, Efficacy and Safety Control / 1 kor., 8, Petrovsky blvrd., 103051, Moscow, Russia						
2604 / Mikhail Ilkovich	Ethical Committee at the Federal Department of Drug Quality, Efficacy and Safety Control / 1 kor., 8, Petrovsky blvrd., 103051, Moscow, Russia	Prof. F.I.Komarov	21-05-02	24-09-03	26-12-05	05-06-07	05-07-07
2605 / Alla Tsoi	Ethical Committee at the Federal Department of Drug Quality, Efficacy and Safety Control / 1 kor., 8, Petrovsky blvrd., 103051, Moscow, Russia	Prof. F.I.Komarov	21-05-02	24-09-03	26-12-05	05-06-07	30-05-07

Singapore							
**Centre number(s) / Investigator(s)**	**IRB or IEC (name/address)**	**IRB or IEC Chairperson**	**Informed Consent/Pt. Information Form Approval DD/MM/YY**	**Protocol Approval DD/MM/YY**	**Am. 1 Approval DD/MM/YY**	**Am. 2 Approval DD/MM/YY**	**Am. 3 Approval DD/MM/YY**
1904 / Yee Tang Wang	National Healthcare Group HQ Domain Specific Review Boards (DSRB) No.6 Commonwealth Lane, GMTI Building, Level 6 Singapore 149547	Dr Goh Boon Cher	21 Apr 2003	21 Apr 2003	24 Aug 2006	24 Aug 2006	04 Jun 2007
1905 / Constance Lo	SGH Institutional Review Board Executive Office C/o Medical Board Blk 7 Level 1 Singapore General Hospital Outram Road Singapore 169608	Dr Aw Swee Eng	24 Apr 2003	24 Apr 2003	14 Aug 2006	14 Aug 2006	23 May 2007
1906 / Lim Tow Keang	National Healthcare Group HQ Domain Specific Review Boards (DSRB) No.6 Commonwealth Lane, GMTI Building, Level 6 Singapore 149547	Dr Goh Boon Cher	21 Apr 2003	21 Apr 2003	24 Aug 2006	24 Aug 2006	04 Jun 2007

Slovakia							
**Centre number(s) / Investigator(s)**	**IRB or IEC (name/address)**	**IRB or IEChairperson**	**Informed Consent/Pt. Information Form Approval DD/MM/YY**	**Protocol Approval DD/MM/YY**	**Am. 1 Approval DD/MM/YY**	**Am. 2 Approval DD/MM/YY**	**Am. 3 Approval DD/MM/YY**
2701 / Peter Kristufek	Eticka komisia NUTaRCH Krajinska cesta 91 SK-825 56 Bratislava	Karol Virsik	24/04/02	24/04/02	N/A	N/A	N/A
2702 / Ladislav Chovan	Eticka komisia NUTaRCH Krajinska cesta 91 SK-825 56 Bratislava	Karol Virsik	24/04/02	24/04/02	05/04/04	22/03/06	explicit approval not required
2703 / Eva Rozborilova	Eticka komisia Jeseniovej LF UK Záborskeho 2 SK-036 59 Martin	Gabriela Nosalova	19/04/02	19/04/02	14/10/03	03/01/06	explicit approval not required
2704 / Dusan Salat	Eticka komisia pri Srobarovom ustave DTaRCh SK-058 01 Dolny Smokovec	Tatiana Michnova	10/04/02	10/04/02	16/12/04	12/01/06	explicit approval not required
2705 / Jan Plutinsky	Eticka komisia Specializovana nemocnica Sv. Svorada Klastorska 134 SK-943 88 Nitra-Zobor	Bohumil Matula	05/09/02	05/09/02	03/09/03	02/03/06	explicit approval not required

Slovenia							
**Centre number(s) / Investigator(s)**	**IRB or IEC (name/address)**	**IRB or IEC Chairperson**	**Informed Consent/Pt. Information Form Approval DD/MM/YY**	**Protocol Approval DD/MM/YY**	**Am. 1 Approval DD/MM/YY**	**Am. 2 Approval DD/MM/YY**	**Am. 3 Approval DD/MM/YY**
2801 / Stanislav Suskovic	National Medical Ethics Committee University Institute of Clinical Neurophysiology, Medical Center Ljubljana, Zaloška c. 7, SI-1525 Ljubljana	Prof. Joze Trontelj, DrSc, MD, *Chairman*	11/06/2002	11/06/2002	16/09/2003	17/01/2006	19/06/2007
2802 / Mitja Kosnik	National Medical Ethics Committee University Institute of Clinical Neurophysiology, Medical Center Ljubljana, Zaloška c. 7, SI-1525 Ljubljana	Prof. Joze Trontelj, DrSc, MD, *Chairman*	11/06/2002	11/06/2002	16/09/2003	17/01/2006	19/06/2007
2803 / Matjaz Turel	National Medical Ethics Committee University Institute of Clinical Neurophysiology, Medical Center Ljubljana, Zaloška c. 7, SI-1525, Ljubljana	Prof. Joze Trontelj, DrSc, MD, *Chairman*	11/06/2002	11/06/2002	16/09/2003	17/01/2006	19/06/2007
2804 / Dr. Leopold Rezar	National Medical Ethics Committee University Institute of Clinical	Prof. Joze Trontelj, DrSc, MD, *Chairman*	11/06/2002	11/06/2002	16/09/2003	17/01/2006	19/06/2007

Slovenia							
**Centre number(s) / Investigator(s)**	**IRB or IEC (name/address)**	**IRB or IEC Chairperson**	**Informed Consent/Pt. Information Form Approval DD/MM/YY**	**Protocol Approval DD/MM/YY**	**Am. 1 Approval DD/MM/YY**	**Am. 2 Approval DD/MM/YY**	**Am. 3 Approval DD/MM/YY**
	Neurophysiology, Medical Center Ljubljana, Zaloška c. 7, SI-1525 Ljubljana						
2805 / Snezana Ulcar-Kostic	National Medical Ethics Committee University Institute of Clinical Neurophysiology, Medical Center Ljubljana, Zaloška c. 7, SI-1525 Ljubljana	Prof. Joze Trontelj, DrSc, MD, *Chairman*	11/06/2002	11/06/2002	16/09/2003	17/01/2006	19/06/2007

South Africa							
**Centre number(s) / Investigator(s)**	**IRB or IEC (name/address)**	**IRB or IEC Chairperson**	**Informed Consent/Pt. Information Form Approval DD/MM/YY**	**Protocol Approval DD/MM/YY**	**Am. 1 Approval DD/MM/YY**	**Am. 2 Approval DD/MM/YY**	**Am. 3 Approval DD/MM/YY**
2901 / G.J. Ras	Pharma-Ethics PO Box 786 Irene 0062	Dr. C. Duvenage	30-07-03	05-06-02	Not applicable to South Africa	12-12-05	15-05-07
2902 / Dr. J.J. du Toit	Pharma-Ethics PO Box 786 Irene 0062	Dr. C. Duvenage	30-07-03	05-06-02	Not applicable to South Africa	12-12-05	15-05-07
2903 / Leon Herbst	Pharma-Ethics PO Box 786 Irene 0062	Dr. C. Duvenage	30-07-03	05-06-02	Not applicable to South Africa	12-12-05	15-05-07
2904 / M. van der Linden	Pharma-Ethics PO Box 786 Irene 0062	Dr. C. Duvenage	30-07-03	05-06-02	Not applicable to South Africa	12-12-05	15-05-07
2905 / A.M. Nel	Pharma-Ethics PO Box 786 Irene 0062	Dr. C. Duvenage	30-07-03	05-06-02	Not applicable to South Africa	12-12-05	15-05-07
2906 / J.J. Jansen	Pharma-Ethics PO Box 786 Irene 0062	Dr. C. Duvenage	30-07-03	05-06-02	Not applicable to South Africa	12-12-05	15-05-07
2908 / F.C.J. Bester	Pharma-Ethics PO Box 786 Irene 0062	Dr. C. Duvenage	30-07-03	05-06-02	Not applicable to South Africa	12-12-05	15-05-07
2909 / H.J.R. Colyn	Pharma-Ethics PO Box 786 Irene 0062	Dr. C. Duvenage	30-07-03	05-06-02	Not applicable to South Africa	12-12-05	15-05-07
2910 / Ismail Abdullah	Pharma-Ethics PO Box 786 Irene 0062	Dr. C. Duvenage	30-07-03	05-06-02	Not applicable to South Africa	12-12-05	15-05-07

South Africa							
**Centre number(s) / Investigator(s)**	**IRB or IEC (name/address)**	**IRB or IEC Chairperson**	**Informed Consent/Pt. Information Form Approval DD/MM/YY**	**Protocol Approval DD/MM/YY**	**Am. 1 Approval DD/MM/YY**	**Am. 2 Approval DD/MM/YY**	**Am. 3 Approval DD/MM/YY**
2911 / Ismail Aboobaker Abdullah	Pharma-Ethics<br>PO Box 786<br>Irene 0062	Dr. C. Duvenage	30-07-03	05-06-02	Not applicable to South Africa	12-12-05	15-05-07
2912 / Eric Bateman	UCT Research Ethics Committee<br>Groote Schuur Hospital<br>Observatory 7925	Prof. M. Blockman	30-07-03	02-11-02	Not applicable to South Africa	19-12-05	08-06-07

Spain								
**Centre number(s) / Investigator(s)**	**IRB or IEC (name/address)**	**IRB or IEC Chairperson**	**Protocol Approval DD/MM/YY**	**Am. 1 Submission DD/MM/YY**	**Informed Consent/Pt. Information form (IB vers. 12) Approval DD/MM/YY**	**Am. 2 Approval DD/MM/YY**	**Informed Consent/Pt. Information form (IB vers. 13) Approval DD/MM/YY**	**Am. 3 Submission DD/MM/YY**
3001 / Josep Lluis Heredia Budó	Hospital Mutua de Terrasa<br>Secretaría del CEIC<br>Plaza Dr. Robert, 5<br>08221 Terrasa	Susana Redondo	31/07/02	09/07/03	01/02/05	01/02/06	28/07/06	29/06/07
3002 / Julio Marin Pardo	Hospital Clínico Universitario de Valencia<br>Secretaría del CEIC<br>Avda. Blasco Ibañez, 17<br>46010 Valencia	Manuel Labios	02/08/02	09/07/03<br><br>24/07/03 Approval	26/01/05	25/01/06	26/07/06	29/06/07
3003 / Marc Miravitlles	Hospital Clínico<br>Comité Ético de Investigación Clínica<br>C/ Villaroel, 170<br>08036- Barcelona	Begoña Gómez	07/10/02	09/07/03	14/01/05	03/01/06	07/09/06	29/06/07
3004 / José Luis Izquierdo Alonso	Hospital Gerenal Universitario de Guadalajara<br>Secretaría del CEIC<br>Donantes de Sangre, s/n<br>19002 Guadalajara	José Antonio Piqueras	24/07/02	09/07/03	27/01/05	27/01/06	14/07/06	29/06/07<br><br>11/07/07 Approval
3005 / Pascual Val Adán	Hospital General San Jorge de Huesca,<br>Secretaría del CEIC,<br>Avda. Martínez de Velasco, 36; 22004 Huesca	Miguel Ángel Domínguez<br><br>María González Hinjos	06/11/02	09/07/03	11/02/05	27/01/06	28/07/06	29/06/07

Spain									
**Centre number(s) / Investigator(s)**	**IRB or IEC (name/address)**	**IRB or IEC Chairperson**	**Protocol Approval DD/MM/YY**	**Am. 1 Submission DD/MM/YY**	**Informed Consent/Pt. Information form (IB vers. 12) Approval DD/MM/YY**	**Am. 2 Approval DD/MM/YY**	**Informed Consent/Pt. Information form (IB vers. 13) Approval DD/MM/YY**	**Am. 3 Submission DD/MM/YY**
	Comité Ético de Investigación Clínica de Aragón (CEICA)<br>Avda. Gómez Laguna, 25 - Planta 9ª<br>50009 Zaragoza							
3006 / Josep Morera Prat	Hospital Universitario Germans Trias i Pujol<br>Secretaría del CEIC<br>Crtra. del Canyet, s/n<br>08916 Badalona	Ángels Fortes	25/10/02	09/07/03	25/02/05	19/01/06	13/07/06	29/06/07
3008 / Jaume Ferrer	Hospital General Universitari Vall d'Hebrón<br>Secretaría del CEIC<br>Passeig de la Vall d'Hebron, 119<br>08035 Barcelona	José Bruno Montero	13/11/02	09/07/03<br>30/09/03 Approval	31/01/05	30/01/06	31/07/06	29/06/07
3009 / José Luis Alvarez-Sala	CEIC regional de la comunidad de Madrid<br>Servicio de Regulación Sanitaria-Unidad de Bioética Viceconsejería de Ordenación Sanitaria y Salud Publica<br>C/ La Aduana, 29 3ªplanta<br>28013 Madrid	Jesús Iñigo Martinez	29/11/02	09/07/03	11/02/05	14/02/06	02/08/06	29/06/07

Spain								
**Centre number(s) / Investigator(s)**	**IRB or IEC (name/address)**	**IRB or IEC Chairperson**	**Protocol Approval DD/MM/YY**	**Am. 1 Submission DD/MM/YY**	**Informed Consent/Pt. Information form (IB vers. 12) Approval DD/MM/YY**	**Am. 2 Approval DD/MM/YY**	**Informed Consent/Pt. Information form (IB vers. 13) Approval DD/MM/YY**	**Am. 3 Submission DD/MM/YY**
3010 / Jesús Fernández Francés	CEIC regional de la comunidad de Madrid Servicio de Regulación Sanitaria-Unidad de Bioética Viceconsejería de Ordenación Sanitaria y Salud Publica C/ La Aduana, 29 3ªplanta 28013 Madrid	Jesús Iñigo Martinez	29/11/02	09/07/03	NA Site closed-out on 23/09/04	NA Site closed-out on 23/09/04	NA Site closed-out on 23/09/04	NA Site closed-out on 23/09/04
3011 / Fernando Fuentes Otero	Complejo Hospitalario Infanta Cristina Secretaría del CEIC Ctra. De Elvas, s/n 06080 Badajoz	Julio Benitez	25/09/02	09/07/03	19/01/05	23/03/06	02/10/06	29/06/07
3013 / Mª José Ferreiro Álvarez	CEIC regional de la comunidad de Madrid Servicio de Regulación Sanitaria-Unidad de Bioética Viceconsejería de Ordenación Sanitaria y Salud Publica C/ La Aduana, 29 3ªplanta 28013 Madrid	Jesús Iñigo Martínez	29/11/02	09/07/03	11/02/05	14/02/06	02/08/06	29/06/07
3014 / Héctor Verea	CEIC Sergas Edificio de la Conselleria de Sanidad San Lázaro, s/n 15703 Santiago de	Maragarita Tasende	12/11/02	09/07/03	28/01/05	07/02/06	28/07/06	29/06/07

Spain								
**Centre number(s) / Investigator(s)**	**IRB or IEC (name/address)**	**IRB or IEC Chairperson**	**Protocol Approval DD/MM/YY**	**Am. 1 Submission DD/MM/YY**	**Informed Consent/Pt. Information form (IB vers. 12) Approval DD/MM/YY**	**Am. 2 Approval DD/MM/YY**	**Informed Consent/Pt. Information form (IB vers. 13) Approval DD/MM/YY**	**Am. 3 Submission DD/MM/YY**
	Compostela							
3015 / Albert Marín Pérez	Corporació Sanitària Parc Taulí de Sabadell Secretaría del CEIC Parc Taulí, s/n, 08208 Sabadell	Coloma Moreno	18/11/02	09/07/03	25/01/05	31/01/06	25/07/06	26/06/07
3016 / Salvador Hernández Flix	Hosp. Univer.de Sant Joan de Reus Secretaría del CEIC Sant Joan /sn 43201-Reus	Blai Coll	17/10/02	09/07/03	25/02/05	26/01/06	13/07/06	29/06/07
3017 / Antonio Sueiro Bendito	CEIC regional de la comunidad de Madrid Servicio de Regulación Sanitaria-Unidad de Bioética Viceconsejería de Ordenación Sanitaria y Salud Publica C/ La Aduana, 29 3ªplanta 28013 Madrid	Jesús Iñigo Martínez	29/11/02	09/07/03	11/02/05	14/02/06	02/08/06	29/06/07
3018 / Rafael Bandrés Gimeno	CEIC Sergas, Edificio de la Conselleria de Sanidad, San Lázaro, s/n, 15703 Santiago de Compostela	Margarita Tasende	12/11/02	09/07/03	28/01/05	07/02/06	28/07/06	29/06/07

Spain									
**Centre number(s) / Investigator(s)**	**IRB or IEC (name/address)**	**IRB or IEC Chairperson**	**Protocol Approval DD/MM/YY**	**Am. 1 Submission DD/MM/YY**	**Informed Consent/Pt. Information form (IB vers. 12) Approval DD/MM/YY**	**Am. 2 Approval DD/MM/YY**	**Informed Consent/Pt. Information form (IB vers. 13) Approval DD/MM/YY**	**Am. 3 Submission DD/MM/YY**
3019 / Antonio Sueiro Bendito	Hospital Virgen Macarena<br>Secretaría del CEIC<br>Avda. de Fedriani, 3<br>41009 Sevilla<br>CEIC Regional de Andalucía<br>Servicio Andaluz de Salud<br>Consejería de Salud<br>Av. Manuel Siurot,s/n<br>41013-Sevilla	Carlos García<br>Antonio Velázquez	20/12/02	09/07/03	09/02/05	27/01/06<br>10/02/06	22/07/06<br>26/10/06	29/06/07
3020 / Carlos Villasante Fernánsez-Montes	CEIC regional de la comunidad de Madrid<br>Servicio de Regulación Sanitaria-Unidad de Bioética Viceconsejería de Ordenación Sanitaria y Salud Publica<br>C/ La Aduana, 29<br>3ªplanta<br>28013 Madrid	Jesús Iñigo Martínez	29/11/02	16/07/03	11/02/05	14/02/06	02/08/06	29/06/07
3021 / Salvador Navas Vazquez	Hospital de Jerez de la Frontera<br>Secretaría del CEIC<br>Ctra. de Circunvalación ,s/n<br>11407-Jérez de la Frontera<br>CEIC Regional de	Asunción Almendral<br>Antonio Velázquez	07/11/02	09/07/03	18/02/05	10/02/06	26/10/06	29/06/07

Spain								
**Centre number(s) / Investigator(s)**	**IRB or IEC (name/address)**	**IRB or IEC Chairperson**	**Protocol Approval DD/MM/YY**	**Am. 1 Submission DD/MM/YY**	**Informed Consent/Pt. Information form (IB vers. 12) Approval DD/MM/YY**	**Am. 2 Approval DD/MM/YY**	**Informed Consent/Pt. Information form (IB vers. 13) Approval DD/MM/YY**	**Am. 3 Submission DD/MM/YY**
	Andalucía<br>Servicio Andaluz de Salud<br>Consejería de Salud<br>Av. Manuel Siurot,s/n<br>41013-Sevilla							
3022 / Victor Sobradillo Peña / Juan Bautista Galdiz	Hospital de Cruces<br>Plaza de Cruces, s/n<br>48903 Barakaldo	Amaya Martínez	04/12/02	09/07/03	09/02/05	25/01/06	20/07/06	03/07/07
3023 / Manuel Rubio Goday	Hospital Universitari de Girona Dr. Josep Trueta<br>Secretaría del CEIC<br>Avda. de França, s/n<br>17007 Girona	Blanca Martínez	17/12/02	09/07/03	25/01/05	01/02/06	19/07/06	29/06/07
3024 / Adolfo Baloira Villar	CEIC Sergas<br>Edificio de la Conselleria de Sanidad<br>San Lázaro, s/n<br>15703 Santiago de Compostela	Margarita Tesende	12/11/02	09/07/03	28/01/05	07/02/06	28/07/06	29/06/07
3025 / Rafael Blanquer Olivas	Hospital Univeristario Dr. Peset, Secretaría del CEIC<br>Avda. Gaspar Aguilar, 90<br>46017 Valencia	Eduardo Moreno	29/11/02	09/07/03	23/02/05	25/01/06	26/07/06	29/06/07
3026 / Dr. Santiago Romero	Hospital General Universitario de Alicante<br>Secretaría del CEIC	Elena López Navarro	18/12/02	09/07/03	27/04/05	31/05/06	26/07/06	29/06/07

Spain								
**Centre number(s) / Investigator(s)**	**IRB or IEC (name/address)**	**IRB or IEC Chairperson**	**Protocol Approval DD/MM/YY**	**Am. 1 Submission DD/MM/YY**	**Informed Consent/Pt. Information form (IB vers. 12) Approval DD/MM/YY**	**Am. 2 Approval DD/MM/YY**	**Informed Consent/Pt. Information form (IB vers. 13) Approval DD/MM/YY**	**Am. 3 Submission DD/MM/YY**
Candeira	Pintor Baeza, s/n<br>03010 Alicante							
3027 / Dr. Alvar Agustí	Complejo Asistencial Son Dureta<br>Secretaría del CEIC<br>C/ Andrea Doria, 55<br>07014 Palma de Mallorca	Magdalena Salom	30/10/02	09/07/03	23/02/05	25/01/06	26/07/06	29/06/07
3028 / Dr. Julio Ancochea	CEIC regional de la comunidad de Madrid<br>Servicio de Regulación Sanitaria-Unidad de Bioética Viceconsejería de Ordenación Sanitaria y Salud Publica<br>C/ La Aduana, 29<br>3ªplanta, 28013 Madrid	Jesús Iñigo	20/11/02	09/07/03	11/02/05	14/02/06	02/08/06	29/06/07
3030 / José María Marín Trigo	Hospital Miguel Servet<br>Secretaría del CEIC<br>Pº Isabel la Católica, 1-2<br>50009 Zaragoza<br>Comité Ético de Investigación Clínica de Aragón (CEICA)<br>Avda. Gómez Laguna, 25 - Planta 9ª<br>50009 Zaragoza	Angela Idoipe<br>María González Hinjos	19/11/02	09/07/03	11/02/05	27/01/06	28/07/06	29/06/07
3031 / José Miguel Rodríguez	CEIC regional de la comunidad de Madrid<br>Servicio de Regulación	Jesús Iñigo	20/11/02	09/07/03	11/02/05	14/02/06	02/08/06	29/06/07

Spain									
**Centre number(s) / Investigator(s)**	**IRB or IEC (name/address)**	**IRB or IEC Chairperson**	**Protocol Approval DD/MM/YY**	**Am. 1 Submission DD/MM/YY**	**Informed Consent/Pt. Information form (IB vers. 12) Approval DD/MM/YY**	**Am. 2 Approval DD/MM/YY**	**Informed Consent/Pt. Information form (IB vers. 13) Approval DD/MM/YY**	**Am. 3 Submission DD/MM/YY**
González-Moro	Sanitaria-Unidad de Bioética Viceconsejería de Ordenación Sanitaria y Salud Publica<br>C/ La Aduana, 29<br>3ªplanta<br>28013 Madrid							
3032 / Benjamín Brotons	Hospital Arnau de Vilanova de Valencia<br>Secretaría del CEIC<br>C/ San Clemente, 26<br>46015 Valencia	Amalia Francés	10/01/03	09/07/03	02/03/05	02/02/06	28/07/06	29/06/07
3033 / Julio Merino	Hospital General Carlos Haya<br>Secretaría del CEIC<br>Avda. Carlos Haya, s/n<br>29010 Málaga<br>CEIC Regional de Andalucía<br>Servicio Andaluz de Salud<br>Consj. de Salud<br>Av. Manuel Siurot,s/n<br>41013-Sevilla	Ramón Utrera<br><br>Antonio Velázquez	10/10/02	09/07/03	20/01/05	10/02/06	26/10/06	29/06/07
3034 / Miguel Perpiñá Tordera	Hospital La Fe de Valencia<br>Secretaría del CEIC<br>Avda. de Campanar, 21<br>46009 Valencia	M.Gobernado Serrano	27/11/02	09/07/03<br><br>07/11/03 (Approval)	07/03/05	25/04/06	08/08/06	29/06/07

Spain								
**Centre number(s) / Investigator(s)**	**IRB or IEC (name/address)**	**IRB or IEC Chairperson**	**Protocol Approval DD/MM/YY**	**Am. 1 Submission DD/MM/YY**	**Informed Consent/Pt. Information form (IB vers. 12) Approval DD/MM/YY**	**Am. 2 Approval DD/MM/YY**	**Informed Consent/Pt. Information form (IB vers. 13) Approval DD/MM/YY**	**Am. 3 Submission DD/MM/YY**
3035 / Dr. Francisco Ortega	Hospital Universitario Virgen del Rocío Secretaría del CEIC Avda. Manuel Siurot, s/n 41013 Sevilla CEIC Regional de Andalucía Servicio Andaluz de Salud Consejería de Salud Av. Manuel Siurot,s/n 41013-Sevilla	Ana Mª Casas<br><br>Antonio Velázquez	11/11/02	09/07/03<br><br>06/10/03 (Approval)	02/02/05	03/02/06<br>10/02/06	26/07/06	29/06/07
3036 / Ángel López Encuentra	CEIC regional de la comunidad de Madrid Servicio de Regulación Sanitaria-Unidad de Bioética Viceconsejería de Ordenación Sanitaria y Salud Publica C/ La Aduana, 29 3ªplanta 28013 Madrid	Jesús Iñigo	20/11/02	09/07/03	11/02/05	14/02/06	02/08/06	29/06/07
3037 / José Julián Batista	Hospital Ntra. Sra. de la Candelaria Secretaría del CEIC Crtra. del Rosario, 145 38010 Santa Cruz de Tenerife	Javier Merino	20/02/03	09/07/03	27/01/05	24/01/06	04/07/06	29/06/07

Spain								
**Centre number(s) / Investigator(s)**	**IRB or IEC (name/address)**	**IRB or IEC Chairperson**	**Protocol Approval DD/MM/YY**	**Am. 1 Submission DD/MM/YY**	**Informed Consent/Pt. Information form (IB vers. 12) Approval DD/MM/YY**	**Am. 2 Approval DD/MM/YY**	**Informed Consent/Pt. Information form (IB vers. 13) Approval DD/MM/YY**	**Am. 3 Submission DD/MM/YY**
3038 / Francisco Suárez Pinilla	Hospital Clínico Lozano Blesa Secretaría del CEIC, Avda. San Juan Bosco, 15, 50009 Zaragoza<br>Comité Ético de Investigación Clínica de Aragón (CEICA), Avda. Gómez Laguna, 25 - Planta 9ª, 50009 Zaragoza	Máximo Bartolomé Mª González Hinjos	12/12/02	09/07/03	11/02/05	27/01/06	28/07/06	29/06/07
3039 / Ramón Agüero	Hospital Universitario Marqués de Valdecilla Secretaría del CEIC Avda. Valdecilla, s/n 39008 Santander	Felipe de la Llama	05/02/03	09/07/03	10/03/05	03/02/06	14/07/06	29/06/07

Switzerland							
**Centre number(s) / Investigator(s)**	**IRB or IEC (name/address)**	**IRB or IEC Chairperson**	**Informed Consent/Pt. Information Form Approval DD/MM/YY**	**Protocol Approval DD/MM/YY**	**Am. 1 Approval DD/MM/YY**	**Am. 2 Approval DD/MM/YY**	**Am. 3 Approval DD/MM/YY**
3201 / Erich Russi	SPUK für Innere Medizin USZ, Dep. Innere Medizin Rämistrasse 100 8091 Zürich (old) Sonneneggstrasse 12 8091 Zürich (new)	Prof. Dr. Jürg Müller (old) Prof. Dr. med. Renato L. Galeazzi (new)	29.08.02	29.08.02	-	09.01.06	31.05.07
3202 / Markus Solèr	Ethikkommission beider BaselEKBB, Hebelstrasse 53 4056 Basel	Prof. Dr. Hans Kummer (old) Prof. Dr. med. André Perruchoud (new)	22.08.02	22.08.02	-	13.01.06	29.05.07
3203 / Michael Tamm	Ethikkommission beider Basel EKBB, Hebelstrasse 53 4056 Basel	Prof. Dr. Hans Kummer (old) Prof. Dr. med. André Perruchoud (new)	22.08.02	22.08.02	-	13.01.06	29.05.07
3204 / Otto Brändli	SPUK für Innere Medizin USZ, Dep. Innere Medizin Rämistrasse 100 8091 Zürich (old) Sonneneggstrasse 12 8091 Zürich (new)	Prof. Dr. Jürg Müller (old) Prof. Dr. med. Renato L. Galeazzi (new)	29.08.02	29.08.02	-	-	-
3205 / Marco Pons	Comitato etico cantonale c/o Sezione sanitaria Via Orico 5, 6501 Bellinzona	Dr. Giovan Maria Zanini	21.11.02	21.11.02	-	06.04.06	21.11.07
3206 / Jean-Marie Tschopp	Commission Cantonale Valaisanne d'Ethique Médicale (CCVEM); Av. Grand Champsec 86 ICHV, 1950 Sion	Prof. Patrick Ravussin	21.10.02	21.10.02	-	28.02.06	12.06.07

Switzerland							
**Centre number(s) / Investigator(s)**	**IRB or IEC (name/address)**	**IRB or IEC Chairperson**	**Informed Consent/Pt. Information Form Approval DD/MM/YY**	**Protocol Approval DD/MM/YY**	**Am. 1 Approval DD/MM/YY**	**Am. 2 Approval DD/MM/YY**	**Am. 3 Approval DD/MM/YY**
3208 / Thomas Hess	SPUK am Kantonsspital Winterthur, Ethikkommission des Kantonsspitals Winterthur Brauerstrasse 15, 8401 Winterthur	Dr. H.P. Keller	17.10.02	17.10.02	-	30.12.05	21.06.07

Taiwan							
**Centre number(s) / Investigator(s)**	**IRB or IEC (name/address)**	**IRB or IEC Chairperson**	**Informed Consent/Pt. Information Form Approval DD/MM/YY**	**Protocol Approval DD/MM/YY**	**Am. 1 Approval DD/MM/YY**	**Am. 2 Approval DD/MM/YY**	**Am. 3 Approval DD/MM/YY**
3301 / Pan-Chyi Yang	Research Ethics Committee NTUIH/ 7, Chung-Shan South Road, Taipei, Taiwan 100, ROC	Pan-Chyr Yang	25/Nov/2002	25/Nov/2002	15/Jun/2004	18/Apr/2007	10/Jul/2007
3302 / Han-Pin Kuo	JIRB/ No.201, Sec 2, Shih-pai Road, Taiwan 11217 ROC	Tsang-Tang Hsieh	05/Aug/2002	05/Aug/2002	15/Jun/2004	27/Sep/2006	07/Aug/2007
3303 / Guang-Ming Shiao	JIRB/ No.201, Sec 2, Shih-pai Road, Taiwan 11217 ROC	Tsang-Tang Hsieh	05/Aug/2002	05/Aug/2002	15/Jun/2004	27/Sep/2006	07/Aug/2007

Thailand							
**Centre number(s) / Investigator(s)**	**IRB or IEC (name/address)**	**IRB or IEC Chairperson**	**Informed Consent/Pt. Information Form Approval DD/MM/YY**	**Protocol Approval DD/MM/YY**	**Am. 1 Approval DD/MM/YY**	**Am. 2 Approval DD/MM/YY**	**Am. 3 Approval DD/MM/YY**
3401 / Pothirat Chaicharn	Research Ethical Committee, Faculty of Medicine, Chiang Mai Uiniversity	Prof. Kumpol Klunklin<br>Assoc.Prof. Sungwal Rugpao<br>Prof.Malai Muttaruk	Version 2.0 (12 Nov 02)<br>Version 3.0 (22 Aug 06)<br>Version 4.0 (22 Aug 06)<br>Long term follow-up (22 Aug 06)<br>Addendum for safety (22 Aug 06); Addendum for transportation (12 Feb 07)	12 Nov 02	23 Sep 03	22 Aug 06	3 Jul 07
3402 / Wongtim Somkiat	The Ethics Committee of The Faculty of Medicine, Chulalongkorn University, Bangkok, Thailand	Prof.Anek Aribarg	Version 1.0 (14 Feb 02)<br>Version 2.0 (17 Aug 05)<br>Version 3.0 (4 Sep 06)<br>Long term follow-up (4 Sep 06)<br>Addendum for safety (4 Sep 06); Addendum for transportation; (11 Jul 07)	5 Jul 02	12 Nov 03	4 Sep 06	21 Jun 07
3403 / Kiatboonsri Sumalee	Ethical Clearance Committee on Human Rights Related to Researches Involving Human Subjects Faculty of Medicine,	Prof.Krisada Ratana-Olarn<br>Prof.Duangrurdee Wattanasirichaigoon	Version 2.0 (15 May 02)<br>Version 3.0 (18 May 03)	15 May 02	26 Nov 03	27 Sep 06	20 Jun 07

Thailand							
**Centre number(s) / Investigator(s)**	**IRB or IEC (name/address)**	**IRB or IEC Chairperson**	**Informed Consent/Pt. Information Form Approval DD/MM/YY**	**Protocol Approval DD/MM/YY**	**Am. 1 Approval DD/MM/YY**	**Am. 2 Approval DD/MM/YY**	**Am. 3 Approval DD/MM/YY**
	Ramathibodi Hospital, Mahidol University	Prof.Boonsong Ongphiphadhanakul	Version 4.0 (27 Sep 06) Long term follow-up (27 Sep 06) Addendum for safety (27 Sep 06) Addendum for transportation (Pending)				
3404 / Wanchai Dejsomritrutai	The Ethical Committee on Research Involving Human Subject Faculty of Medicine, Siriraj Hospital, Mahidol University	Prof.Sumalee Nimmannit Prof.Shusee Visalyaputra	Version 2.0 (19 Jul 02) Version 3.0 (N/A) Version 4.0 (18 Aug 06) Long term follow-up (18 Aug 06) Addendum for safety (18 Aug 06) Addendum for transportation (Pending)	19 Jul 02	10 Oct 03	18 Aug 06	22 Jun 07
3405 / Boonsawat Watchara	The Khon Kean University Ethics Committee for Human Research	Prof.Pisak Lumbiganon Assoc.Prof.Verachai Kowsuwan Prof.Pyatat Tatsanavivat Assoc.Prof.Jiraporn	Version 2.0 (15 Aug 02) Version 3.0 (17 Aug 06) Version 4.0 (17 Aug 06) Long term follow-	15 Aug 02	16 Oct 03	17 Aug 06	22 Jun 07

Thailand							
**Centre number(s) / Investigator(s)**	**IRB or IEC (name/address)**	**IRB or IEC Chairperson**	**Informed Consent/Pt. Information Form Approval DD/MM/YY**	**Protocol Approval DD/MM/YY**	**Am. 1 Approval DD/MM/YY**	**Am. 2 Approval DD/MM/YY**	**Am. 3 Approval DD/MM/YY**
		Srinakarin	up<br>(17 Aug 06)<br>Addendum for safety<br>(17 Aug 06)<br>Addendum for transportation<br>(16 Feb 07)				

Turkey							
**Centre number(s) / Investigator(s)**	**IRB or IEC (name/address)**	**IRB or IEC Chairperson**	**Informed Consent/Pt. Information Form Approval DD/MM/YY**	**Protocol Approval DD/MM/YY**	**Am. 1 Approval DD/MM/YY**	**Am. 2 Approval DD/MM/YY**	**Am. 3 Approval DD/MM/YY**
3501 / Muzeyyen Erk	Istanbul Universitesi Cerrahpasa Tip Fakultesi Yerel Etik Kurulu Cerrahpasa-Istanbul	Prof. Dr. Ercument Olmez	19 JUN 2002	19 JUN 2002	NA	23 FEB 2006	16 OCT 2007
3502 / Esin Tuncay	Yedikule Egt. Ve Aras. Hastanesi Yerel Etik Kurulu Zeytinburnu-Istanbul	Assoc. Prof. Dr. Sedat Altin	10 JUN 2002	10 JUN 2002	NA	06 JAN 2006	16 JAN 2008
3503 / Turgay Celikel	Marmara Universitesi Tip Fakultesi Yerel Etik Kurulu Haydarpasa-Istanbul	Prof. Dr. Haner Direskeneli	29 AUG 2002	29 AUG 2002	NA	03 MAR 2006	21 JUL 2007
3504 / Benan Caglayan	Dr. Lutfi Kirdar Kartal Egt. ve Arastirma Hastanesi Yerel Etik Kurulu Kartal-Istanbul	Assoc. Prof. Dr. Ulku Turk	22 AUG 2002	22 AUG 2008	NA	26 JAN 2006	31 MAY 2007
3505 / Levent Tabak	Istanbul Universitesi Istanbul Tip Fakultesi Yerel Etik Kurulu CAPA-Istanbul	Prof. Dr. Zafer Ari	11 SEP 2002	11 SEP 2002	NA	20 MAR 2006	20 JUN 2007
3506 / Lütfi Çöplü	Hacettepe Universitesi Tip Fakultesi Yerel Etik Kurulu-Sihhiye-Ankara	Prof. Dr. Rustu Onur	05 JUN 2002	05 JUN 2002	NA	15 FEB 2006	26 JUN2007
3507 / Sadik Ardiç	SB. Diskapi Egt. ve Arastirma Hastanesi Yerel Etik Kurulu Diskapi-Ankara	Prof. Dr. Metin Aydin	18 JUL 2002	18 JUL 2002	NA	02 FEB 2006	02 NOV 2007
3508 / Nazire Ucar	Ataturk Gög. Hast. Ve Gogus Cerrahi Merkezi Eğitim ve Arastirma Hast. Yerel Etik Kurulu Sanatoryum -Ankara	Assoc. Prof. Dr. Yurdanur Erdogan	31 MAY 2002	31 MAY 2002	NA	20 JAN 2006	27 FEB 2008
3509 / Arzu Mirici	Ataturk Universitesi Tip Fakultesi Yerel Etik Kurulu-Yakutiye-Erzurum	Prof. Dr. Zerrin Orbak	24 JUL 2002	24 JUL 2002	NA	09 JAN 2006	29 MAY 2007

Turkey							
**Centre number(s) / Investigator(s)**	**IRB or IEC (name/address)**	**IRB or IEC Chairperson**	**Informed Consent/Pt. Information Form Approval DD/MM/YY**	**Protocol Approval DD/MM/YY**	**Am. 1 Approval DD/MM/YY**	**Am. 2 Approval DD/MM/YY**	**Am. 3 Approval DD/MM/YY**
3510 / Ali Kocabas	Cukurova Universitesi Tip Fakultesi Yerel Etik Kurulu Balcali-Adana	Prof. Dr. Selim Kadioglu	12 JUN2002	12 JUN 2002	NA	03 FEB 2006	09 NOV 2007
3511 / Sinan Erginel	Osmangazi Universitesi Tip Fakultesi Yerel Etik Kurulu Eskisehir	Prof. Dr. Zubeyir Kilic	10 JUN 2002	10 JUN 2002	NA	28 FEB 2006	27 JUL 2007
3512 / Arif Hikmet Çimrin	Dokuz Eylul Tip Fakultesi Yerel Etik Kurulu Izmir	Prof. Dr. Meral Koyuncuoglu	11 JUL 2002	11 JUL 2002	NA	08 MAY 2006	07 FEB 2008

UK								
**Centre number(s) / Investigator(s)**	**IRB or IEC (name/address)**	**IRB or IEC Chairperson**	**Informed Consent/Pt. Information Form Approval DD/MM/YY**	**Protocol Approval DD/MM/YY**	***Am. 1 Approval DD/MM/YY**	***Am. 2 Approval DD/MM/YY**	***Am. 3 Approval DD/MM/YY**	***Am. 1 (UK) Approval DD/MM/YY**
Overall Country Approval	South West Multi Centre Research Ethics Committee, The Lescaze Offices, Skinner's Bridge Dartington Devon TQ9 6JE	Mr Barrie Behenna	10/02/03	10/02/03	Not approved in the UK	Not approved in the UK	17/05/07	17/05/07
3601 / Dr. Adel Mansur	East Birmingham Local Research Ethics Committee, Birmingham Heartlands Hospital, Bordesley Green East, Birmingham B9 5SS	Dr S Rose	03/04/03	03/04/03	N/A	N/A	N/A	N/A
3602 / Christine Bucknall	North Glasgow University Hospital NHS Trust, R&D Office, Glasgow Royal Infirmary, 4th Floor, QEB University Block, 10 Alexandra Parade, Glasgow G31 2ER	Dr J B Neilly	19/03/03	19/03/03	N/A	N/A	N/A	N/A
3603 / Paul Fletcher	South West Peninsula NHS, Plymouth Local Research Ethics Committee, Room 101B, TTC South Building, Tamar Science Park, Davy Road, Derriford, Plymouth PL6 8BX	Mr A J R Beauchamp	28/08/03	28/08/03	N/A	N/A	N/A	N/A

UK								
**Centre number(s) / Investigator(s)**	**IRB or IEC (name/address)**	**IRB or IEC Chairperson**	**Informed Consent/Pt. Information Form Approval DD/MM/YY**	**Protocol Approval DD/MM/YY**	***Am. 1 Approval DD/MM/YY**	***Am. 2 Approval DD/MM/YY**	***Am. 3 Approval DD/MM/YY**	***Am. 1 (UK) Approval DD/MM/YY**
3604 / Paul Griffiths	North Manchester Local Research Ethics Committee, Gateway House, Piccadilly South, Manchester M60 7LP	Mrs Gillian Rimmington	06/03/03	06/03/03	N/A	N/A	N/A	N/A
3605 / David Halpin	North and East Devon LREC, Department of Research Ethics and Medical Affairs, Old Kenn Ward Royal Devon & Exeter Hospital, Barrack Road, Exeter EX2 5DW	Dr Terry Jones	26/03/03	26/03/03	N/A	N/A	N/A	N/A
3607 / Michael Morgan	Leicestershire Local Research Ethics Committee (Committee Two), Lakeside House, 4 Smith Way, Grove Park Enderby Leicester LE19 1SS	Dr D Heney	14/04/03	14/04/03	N/A	N/A	N/A	N/A
3608 / Alyn Morice	Hull and East Riding LREC, Room C24, College House, Willerby Hill Business Park, Willerby HU10 6NS	Prof S R Killick	19/03/03	19/03/03	N/A	N/A	N/A	N/A

UK								
**Centre number(s) / Investigator(s)**	**IRB or IEC (name/address)**	**IRB or IEC Chairperson**	**Informed Consent/Pt. Information Form Approval DD/MM/YY**	**Protocol Approval DD/MM/YY**	***Am. 1 Approval DD/MM/YY**	***Am. 2 Approval DD/MM/YY**	***Am. 3 Approval DD/MM/YY**	***Am. 1 (UK) Approval DD/MM/YY**
3609 / Paul Anderson	Sheffield Teaching Hospitals NHS Trust, North Sheffield Ethics Office, 1st Floor, Vickers Corridor, Northern General Hospital, Herries Road, Sheffield S5 7AU	Dr S R Brennan	06/03/03	06/03/03	N/A	N/A	N/A	N/A
3610 / Paul Plant	The Leeds Teaching Hospitals NHS Trust, Local Research Ethics Committee, Room 5.1, Clinical Sciences Building, St James's University Hospital, Beckett Street, Leeds LS9 7TF	Dr P R F Dear	20/03/03	20/03/03	N/A	N/A	N/A	N/A
3611 / Mark Britton	North West Surrey Local Research Ethics Committee, Bournewood House, St Peters Hospital Site, Guildford Road, Chertsey, Surrey KT16 0QA	Mrs Anne Damerell	31/03/03	31/03/03	N/A	N/A	N/A	N/A
3612 / Timothy Harrison	Nottingham Research Ethics Committee 1, 1 Standard Court, Park Row, Nottingham NG1 6GN	Dr D Pearson	14/04/03	14/04/03	N/A	N/A	N/A	N/A

UK								
**Centre number(s) / Investigator(s)**	**IRB or IEC (name/address)**	**IRB or IEC Chairperson**	**Informed Consent/Pt. Information Form Approval DD/MM/YY**	**Protocol Approval DD/MM/YY**	***Am. 1 Approval DD/MM/YY**	***Am. 2 Approval DD/MM/YY**	***Am. 3 Approval DD/MM/YY**	***Am. 1 (UK) Approval DD/MM/YY**
3613 / Stephen Crooks	North Derbyshire Local Research Ethics Committee, Scarsdale, Newbold Road, Chesterfield, Derbyshire S41 7PF	Mr Jonathan O Harris	19/03/03	19/03/03	N/A	N/A	N/A	N/A
3614 / Tim Griffiths	South East Wales Local Research Ethics Committees, Business Services Centre, Churchill House, 17 Churchill Way, Cardiff CF10 2TW	Dr D E B Powell	20/03/03	20/03/03	N/A	N/A	N/A	N/A
3615 / Dr. Graham Douglas	Grampian Research Ethics Committee (1), NHS Grampian, Summerfield House, 2 Eday Road, Aberdeen AB15 6RE	Dr John Dean	03/03/03	03/03/03	N/A	N/A	N/A	N/A
3616 / Jane Gravil	Argyll & Clyde NHS Board, Local Research Ethics Committee, Ross House, Hawkhead Road, Paisley PA2 7BN	Mrs L McKichan	01/04/03	01/04/03	N/A	N/A	N/A	N/A
* UK legislation requires that MREC approved amendments are submitted to LREC for information only so approval is not required.								

USA							
**Centre number(s) / Investigator(s)**	**IRB or IEC (name/address)**	**IRB or IEC Chairperson**	**Informed Consent/Pt. Information Form Approval DD/MM/YY**	**Protocol Approval DD/MM/YY**	**Am. 1 Approval DD/MM/YY**	**Am. 2 Approval DD/MM/YY**	**Am. 3 Approval DD/MM/YY**
3701 / Murray Altose	MetroHealth System Institutional Review Board<br>MetroHealth Medical Center<br>2500 MetroHealth Dr.<br>Cleveland, OH 44109	Ben Brouhard, 2002 Thomas Swales, 2004 David Kuentz 2006-2007	11-July-02	11-July-02	30-Jul-03	18-Jan-06	03-Oct-07
3703 / Theodore Amgott	Western Institutional Review Board<br>3535 Seventh Avenue, SW<br>Olympia, WA 98502-5010	William C. Jacobs, 2002-2004<br>Theodore D. Schultz, 2005-2008	23-May 02	23-May 02	11-Jul-03	21-Dec-05	23-May-07
3704 / Charles Andrews	Western Institutional Review Board<br>3535 Seventh Avenue, SW<br>Olympia, WA 98502-5010	William C. Jacobs, 2002-2004<br>Theodore Schultz, 2005-2008	16-May-02	16-May-02	28-Jul-03	21-Dec-05	23-May-07
3706 / Claudia Cote	Bay Pines VA Medical Center<br>Bay Pines VAMC Institutional Review Board<br>10000 Bay Pines Blvd<br>Bldg 22 Rm 123<br>St. Petersburgh, FL 33708	Lynne Krop, 2002<br>Pedro Vera, 2003<br>Katherine Meyer-Siegler, 2004<br>Rachel McArdle,	8-Aug-02	8-Aug-02	15-Aug-03	12-Jan-06	22-June-07

USA							
**Centre number(s) / Investigator(s)**	**IRB or IEC (name/address)**	**IRB or IEC Chairperson**	**Informed Consent/Pt. Information Form Approval DD/MM/YY**	**Protocol Approval DD/MM/YY**	**Am. 1 Approval DD/MM/YY**	**Am. 2 Approval DD/MM/YY**	**Am. 3 Approval DD/MM/YY**
		2007					
3707 / Francis Averill	Western Institutional Review Board<br>3535 Seventh Avenue, SW<br>Olympia, WA 98502-5010	William C. Jacobs, 2002-2004<br>Theodore D. Schultz, 2005-2008	13-June-02	13-June-02	28-July-03	21-Dec-05	23-May-07
3708 / William Bailey	University of Alabama at Birmingham Institutional Review Board<br>470 Administration Building<br>701 20th Street South<br>Birmingham, AL 35294-0104	Ferdinand Urthaler, 2002-2007	25-Sep-02	25-Sep-02	11-Aug-03	1-Feb-06	17-May-07
3711 / George Bensch	Western Institutional Review Board<br>3535 Seventh Avenue, SW<br>Olympia, WA 98502-5010	William C. Jacobs, 2002-2004<br>Theodore D. Schultz, 2005-2008	20-Jun-02	20-Jun-02	28-July-03	21-Dec-05	23-May-07
3712 / Eugene R. Bleecker	The Office of Research and Development<br>Wake Forest University School of Medicine<br>Medical Center Blvd<br>Winston-Salem, NC 27157	Anthony Liquiri 2007<br>Richard Weinberg 2008	17-July-02	17-July-02	20-Aug-03	16-Jan-07	2-May-07

USA							
**Centre number(s) / Investigator(s)**	**IRB or IEC (name/address)**	**IRB or IEC Chairperson**	**Informed Consent/Pt. Information Form Approval DD/MM/YY**	**Protocol Approval DD/MM/YY**	**Am. 1 Approval DD/MM/YY**	**Am. 2 Approval DD/MM/YY**	**Am. 3 Approval DD/MM/YY**
3713 / David Koh	Western Institutional Review Board<br>3535 Seventh Avenue, SW<br>Olympia, WA 98502-5010	William C. Jacobs, 2002-2004<br>Theodore D. Schultz, 2005-2008	23-May-02	23-May-02	28-Jul-03	21-Dec-05	23-May-07
3715 / Sidney Braman	Committee on the Protection of Human Subjects<br>593 Eddy Street<br>Providence, RI 02903	Penelope Dennehy, 2002-2005	11-Oct-02	11-Oct-02	Did not submit All patients completed visit 2 prior to amendment	22-Dec-05	15-May-07
3716 / Alfred Lynn Brannen	Western Institutional Review Board<br>3535 Seventh Avenue, SW<br>Olympia, WA 98502-5010	William C. Jacobs, 2002-2004<br>Theodore D. Schultz, 2005-2008	22-Aug-02	22-Aug-02	28-Jul-03	21-Dec-05	23-May-07
3717 / Shari Ann Brazinsky	Western Institutional Review Board<br>3535 Seventh Avenue, SW<br>Olympia, WA 98502-5010	William C. Jacobs, 2002-2004<br>Theodore D. Schultz, 2005-2008	16-May-02	16-May-02	28-Jul-03	21-Dec-05	23-May-07

USA							
**Centre number(s) / Investigator(s)**	**IRB or IEC (name/address)**	**IRB or IEC Chairperson**	**Informed Consent/Pt. Information Form Approval DD/MM/YY**	**Protocol Approval DD/MM/YY**	**Am. 1 Approval DD/MM/YY**	**Am. 2 Approval DD/MM/YY**	**Am. 3 Approval DD/MM/YY**
3718 / Timothy Bruya	Western Institutional Review Board 3535 Seventh Avenue, SW Olympia, WA 98502-5010	William C. Jacobs, 2002-2004 Theodore D. Schultz, 2005-2008	13-Jun-02	13-Jun-02	28-Jul-03	21-Dec-05	23-May-07
3721 / Bartolome Celli	Research/Human Subjects Committee St. Elizabeth's Medical Center 736 Cambridge Street Boston, MA 02135	Alan Ashare, 2002-2006	28-Oct-02	9-Jul-02	8-Sep-03	25-Jan-06	07-Apr-08
3722 / John. J. Condemi	Western Institutional Review Board 3535 Seventh Avenue, SW Olympia, WA 98502-5010	William C. Jacobs, 2002-2004 Theodore D. Schultz, 2005-2006	9-May-02	9-May-02	28-Jul-03	21-Dec-05	23-May-07
3723 / Bruce Corser	Western Institutional Review Board 3535 Seventh Avenue, SW Olympia, WA 98502-5010	William C. Jacobs, 2002-2004 Theodore D. Schultz, 2005-2006	6-Jun-02	6-Jun-02	28-Jul-03	21-Dec-05	23-May-07
3724 / James Osmanski	Western Institutional Review Board 3535 Seventh Avenue, SW Olympia, WA 98502-5010	William C. Jacobs, 2002-2004 Theodore D. Schultz, 2005-	12-Sept-02	12-Sept-02	28-Jul-03	21-Dec-05	23-May-07

USA							
**Centre number(s) / Investigator(s)**	**IRB or IEC (name/address)**	**IRB or IEC Chairperson**	**Informed Consent/Pt. Information Form Approval DD/MM/YY**	**Protocol Approval DD/MM/YY**	**Am. 1 Approval DD/MM/YY**	**Am. 2 Approval DD/MM/YY**	**Am. 3 Approval DD/MM/YY**
		2008					
3725 / Timothy Craig	Penn State College of Medicine The Milton S. Hershey Medical Center, Institutional Review Board, Human Subjects Projection Office, 500 University Drive, H112, Hershey, PA 17033	Kevin Gleeson, 2001-2006	30-Jul-02	30-Jul-02	08-Jul-03	04-Jan-06	02-May-07
3727 / Arthur Degraff	Western Institutional Review Board 3535 Seventh Avenue, SW Olympia, WA 98502-5010	William C. Jacobs, 2002-2004 Theodore D. Schultz, 2005-2008	23-May-02	23-May-02	28-Jul-03	21-Dec-05	23-May-07
3729 / Dennis E. Doherty	University of Kentucky Institutional Review Board Room 315 Kinkeed Hall Lexington, KY 40506	Thomas Foster, 2003 Edward Hirschowitz, 2002	25-Jul-02	25-Jul-02	12-Aug-03	11-Jan-06	05 May 2007
3730 / Dr. Anderson	Western Institutional Review Board 3535 Seventh Avenue, SW Olympia, WA 98502-5010	William C. Jacobs, 2002-2004 Theodore D. Schultz, 2005-2008	18-Apr-02	18-Apr-02	28-Jul-03	21-Dec-05	01-May-07

USA							
**Centre number(s) / Investigator(s)**	**IRB or IEC (name/address)**	**IRB or IEC Chairperson**	**Informed Consent/Pt. Information Form Approval DD/MM/YY**	**Protocol Approval DD/MM/YY**	**Am. 1 Approval DD/MM/YY**	**Am. 2 Approval DD/MM/YY**	**Am. 3 Approval DD/MM/YY**
3731 / James Donohue	The Committee on the Protection of the Rights of Human Subjects University of North Carolina at Chapel Hill CB# 7097, Medical School Building 52 Chapel Hill, NC 27599-7097	Stephen Bernard, 2008 Robert Lowman, 2002	04-Oct-02	04-Oct-02	22-Aug-03	15-Mar-06	10-May-07
3732 / Michael Eichenhorn	Human Rights Committee (IRB) Henry Ford Hospital 2799 West Grand Boulevard, CFP-B Detroit, MI 48202	Ajlouni Muntler, 2002 Timothy Roehrs, 2003-2004	04-Jun-02	04-Jun-02	11-Jul-03	02-Feb-06	02-May-07
3734 / Neil Ettinger	St. Luke's Hospital IRB 232 South Woods Mill Road Chesterfield, MO 63017	John Meyer, 2002-2006	28-Jul-02	28-Jul-02	25-Jul-03	25-Jan-06	27-Jun-07
3736 / Dr. Joseph A. Govert	Duke University IRB Box 2991 Duke University Medical Center 2400 Pratt Street, Suite 9000 Durham, NC 27708	John Falletta, 2002 John Harrelson 2006-2007	16-Oct-02	16-Oct-02	24-Jul-03	17-Apr-06	16-May-07

USA							
**Centre number(s) / Investigator(s)**	**IRB or IEC (name/address)**	**IRB or IEC Chairperson**	**Informed Consent/Pt. Information Form Approval DD/MM/YY**	**Protocol Approval DD/MM/YY**	**Am. 1 Approval DD/MM/YY**	**Am. 2 Approval DD/MM/YY**	**Am. 3 Approval DD/MM/YY**
3737 / Dr. Joseph Lasky	Committee on Use of Human Subjects TW 36<br>Office of the Dean<br>Tulane School of Medicine<br>1430 Tulane Avenue<br>Tidewater Building, Room 830<br>New Orleans, LA 70112-2699	Ina Friedman, 2001-2004<br>Mark James 2006-2007	06-Jan-02	06-Jan-02	8-Jul-03	16-Jan-06	31-Jul-07
3738 / Glenn Giessel	Western Institutional Review Board<br>3535 Seventh Avenue, SW<br>Olympia, WA 98502-5010	William C. Jacobs, 2002-2004<br>Theodore D. Schultz, 2005-2008	18-Apr-02	18-Apr-02	28-Jul-03	21-Dec-05	23-May-07
3739 / Dr. Richard Pomerantz	Western Institutional Review Board<br>3535 Seventh Avenue, SW<br>Olympia, WA 98502-5010	William C. Jacobs, 2002-2004<br>Theodore D. Schultz, 2005-2008	02-May-02	02-May-02	27-Aug-03	21-Dec-05	23-May-07
3741 / Gary I. Greenwald	Western Institutional Review Board<br>3535 Seventh Avenue, SW<br>Olympia, WA 98502-5010	William C. Jacobs, 2002-2004<br>Theodore D. Schultz, 2005-	02-May-02	02-May-02	28-Jul-03	21-Dec-05	23-May-07

USA							
**Centre number(s) / Investigator(s)**	**IRB or IEC (name/address)**	**IRB or IEC Chairperson**	**Informed Consent/Pt. Information Form Approval DD/MM/YY**	**Protocol Approval DD/MM/YY**	**Am. 1 Approval DD/MM/YY**	**Am. 2 Approval DD/MM/YY**	**Am. 3 Approval DD/MM/YY**
		2008					
3742 / Nicola Hanania	IRB for Human Subject Research for Baylor College of Medicine and Afflicted Hospitals<br>One Baylor Plaza, 600D<br>Houston, TX 77030	Kathleen Motil, PhD<br>Stacey Berg, 2001-2003	04-Jun-02	04-Jun-02	10-Sep-03	12-Jan-06	12-Jun-07
3743 / Raymond Dean Hautamaki	Sarasota Memorial Hospital<br>Institutional Review Board<br>1700 South Tamiami Trail<br>Sarasota, FL 34239	Mark Magenheim MD, PhD<br>John Gallagher	15-Jul-02	15-Jul-02	25-Aug-03	22-Feb-06	05-Jul-07
3744 / William Henderson IV	Western Institutional Review Board<br>3535 Seventh Avenue, SW<br>Olympia, WA 98502-5010	William C. Jacobs, 2002-2004<br>Theodore D. Schultz, 2005-2008	18-Apr-02	18-Apr-02	28-Jul-03	21-Dec-05	23-May-07
3745 / Albrecht Heyder	Western Institutional Review Board<br>3535 Seventh Avenue, SW<br>Olympia, WA 98502-5010	William C. Jacobs, 2002-2004<br>Theodore D. Schultz, 2005-2008	01-Aug-02	16-May-02	28-Jul-03	21-Dec-05	23-May-07

USA							
**Centre number(s) / Investigator(s)**	**IRB or IEC (name/address)**	**IRB or IEC Chairperson**	**Informed Consent/Pt. Information Form Approval DD/MM/YY**	**Protocol Approval DD/MM/YY**	**Am. 1 Approval DD/MM/YY**	**Am. 2 Approval DD/MM/YY**	**Am. 3 Approval DD/MM/YY**
3747 / Thomas Hyers	Western Institutional Review Board<br>3535 Seventh Avenue, SW<br>Olympia, WA 98502-5010	William C. Jacobs, 2002-2004<br>Theodore D. Schultz, 2005-2008	20-Jun-02	20-Jun-02	28-Jul-03	21-Dec-05	23-May-07
3748 / Jonathan Ilowite	Wintrop-University Hospital<br>Institutional Review Board<br>222 Station Plaza North<br>Suite 521<br>Mineola, NY 11501	Jonathon Davis, 2002-2006	10-Sep-02	20-Jun-02	8-Jul-03	16-Feb-06	07-Jun-07
3749 / Mark James	Western Institutional Review Board<br>3535 Seventh Avenue, SW<br>Olympia, WA 98502-5010	William C. Jacobs, 2002-2004<br>Theodore D. Schultz, 2005-2008	16-May-02	16-May-02	28-Jul-03	21-Dec-05	23-May-07
3750 / Dr. Richard Kahn	Western Institutional Review Board<br>3535 Seventh Avenue, SW<br>Olympia, WA 98502-5010	William C. Jacobs, 2002-2004<br>Theodore D. Schultz, 2005-2008	06-Jun-02	06-Jun-02	28-Jul-03	21-Dec-05	23-May-07
3751 / Harold Kaiser	Western Institutional Review Board<br>3535 Seventh Avenue, SW<br>Olympia, WA 98502-5010	William C. Jacobs, 2002-2004<br>Theodore D. Schultz, 2005-	09-May-02	09-May-02	28-Jul-03	21-Dec-05	23-May-07

USA							
**Centre number(s) / Investigator(s)**	**IRB or IEC (name/address)**	**IRB or IEC Chairperson**	**Informed Consent/Pt. Information Form Approval DD/MM/YY**	**Protocol Approval DD/MM/YY**	**Am. 1 Approval DD/MM/YY**	**Am. 2 Approval DD/MM/YY**	**Am. 3 Approval DD/MM/YY**
		2008					
3752 / Richard E. Kanner	University of Utah Institutional Review Board<br>50 North Medical Drive<br>101 MREB<br>Salt Lake City, UT 84132	Mark Munger, 2002-2005	26-Aug-02	26-Aug-02	6-Mar-03	11-Apr-06	1-Aug-07
3754 / Mitchell Kaye	Western Institutional Review Board<br>3535 Seventh Avenue, SW<br>Olympia, WA 98502-5010	William C. Jacobs, 2002-2004<br>Theodore D. Schultz, 2005-2006	18-Jul-02	18-Jul-02	28-Jul-03	21-Dec-05	23-May-07
3756 / Stephen Kreitzer	Western Institutional Review Board<br>3535 Seventh Avenue, SW<br>Olympia, WA 98502-5010	William C. Jacobs, 2002-2004<br>Theodore D. Schultz, 2005-2006	27-Jun-02	27-Jun-02	28-Jul-03	21-Dec-05	23-May-07
3757 / Dennis Lawlor	Western Institutional Review Board<br>3535 Seventh Avenue, SW<br>Olympia, WA 98502-5010	William C. Jacobs, 2002-2004<br>Theodore D. Schultz, 2005-2006	11-Jul-02	11-Jul-02	15-Sept-03	21-Dec-05	23-May-07

USA							
Centre number(s) / Investigator(s)	IRB or IEC (name/address)	IRB or IEC Chairperson	Informed Consent/Pt. Information Form Approval DD/MM/YY	Protocol Approval DD/MM/YY	Am. 1 Approval DD/MM/YY	Am. 2 Approval DD/MM/YY	Am. 3 Approval DD/MM/YY
3758 / Irene Leech	St. Mary's Medical Center Institutional Review Board 1050 Linden Avenue Long Beach, CA 90813	Phill Madden, 2002	02-Aug-02	02-Aug-02	11-Aug-03	10-Jan-07	08-Apr-08
3759 / Bernard Levine	Western Institutional Review Board 3535 Seventh Avenue, SW Olympia, WA 98502-5010	William C. Jacobs, 2002-2004 Theodore D. Schultz, 2005-2006	03-Jul-02	03-Jul-02	28-Jul-03	21-Dec-05	23-May-07
3760 / Michael Littner	Research and Development Human Subjects Sub-committee VA Greater Los Angeles Healthcare System 1611 Plummer Street (111p) Sepulveda, CA 91343	Ronald Szymusiak 2002-2004 Elizabeth Yano 2007	21-Oct-02	21-Oct-02	28-Jul-03	23-Jan-06	19-July-07
3761 / Nabil Jarmukli	Human Studies Subcommittee VA Medical Center 1970 Roanoke Blvd. Salem, VA 24153	Kim Kye, 2001 Kim Ragsdale, 2004	07-May-02	07-May-02	02-Sep-03	13-Feb-06	01-Aug-07
3762 / Daniel G. Lorch	Western Institutional Review Board 3535 Seventh Avenue, SW Olympia, WA 98502-5010	William C. Jacobs, 2002-2004 Theodore D. Schultz, 2005-	03-Jul-02	03-Jul-02	28-Jul-03	21-Dec-05	23-May-07

USA							
**Centre number(s) / Investigator(s)**	**IRB or IEC (name/address)**	**IRB or IEC Chairperson**	**Informed Consent/Pt. Information Form Approval DD/MM/YY**	**Protocol Approval DD/MM/YY**	**Am. 1 Approval DD/MM/YY**	**Am. 2 Approval DD/MM/YY**	**Am. 3 Approval DD/MM/YY**
		2008					
3763 / Donald Mahler	Committee for the Protection of Human Subjects<br>Office of Grants and Contracts<br>11 Rope Ferry Road #6210<br>Hanover, NH 03755-1404	Eric Larsen 2006-2007	17-Jul-02	17-Jul-02	29-Aug-03	12-Dec-05	16-May-07
3764 / B. Make	National Jewish Medical and Research Center Institutional Review Board<br>1400 Jackson Street<br>Denver Colorado 80206	Henry Milgram Richard Meehan	19-Sep-02	29-Aug-02	17-Jul-03	28-Dec-05	16-May-07
3765 / Michael Mandel	Western Institutional Review Board, 3535 Seventh Avenue, SW, Olympia, WA 98502-5010	William C. Jacobs, 2002-2004; Theodore D. Schultz, 2005-2008	14-Aug-02	14-Aug-02	28-Jul-03	21-Dec-05	23-May-07
3768 / Praveen Mathur	IUPI and clarion IRB<br>Research & Sponsored Program<br>620 Union Drive Rm 618<br>Indianapolis, IN 46202-	C. Conrad Johnston, MD, 2004 - 2008	14-Aug-02	14-Aug-02	17-Jul-03	Did not submit to IRB	26-Jun-07

USA							
**Centre number(s) / Investigator(s)**	**IRB or IEC (name/address)**	**IRB or IEC Chairperson**	**Informed Consent/Pt. Information Form Approval DD/MM/YY**	**Protocol Approval DD/MM/YY**	**Am. 1 Approval DD/MM/YY**	**Am. 2 Approval DD/MM/YY**	**Am. 3 Approval DD/MM/YY**
	5167						
3770 / Delbert H. Meyer	Western Institutional Review Board<br>3535 Seventh Avenue SW<br>Olympia, WA 98502	William C. Jacobs, 2002-2004<br>Theodore D. Schultz, 2005-2008	03-Jul-02	03-Jul-02	28-Jul-03	21-Dec-05	23-May-07
3771 / Michael G. Milam	Western Institutional Review Board<br>3535 Seventh Avenue, SW<br>Olympia, WA 98502-5010	William C. Jacobs, 2002-2004<br>Theodore D. Schultz, 2005-2008	02-May-02	02-May-02	28-Jul-03	21-Dec-05	23-May-07
3773 / Zab Mosenifar	Cedars Sinai Medical CSHS Institutional Review Board<br>83833 Wilshire Blvd. Suite 302<br>Los Angeles, CA 90211	William C. Jacobs, 2002<br>Scott Cumeen, 2002-2004<br>Stephen Lim, 2005	27-Sept-02	27-Sept-02	16-Jul-03	07-Feb-06	15-Jun-07
3775 / Timothy Moriarty	Western Institutional Review Board<br>3535 Seventh Avenue, SW<br>Olympia, WA 98502-5010	William C. Jacobs, 2002-2004<br>Theodore D. Schultz, 2005-2006	25-Jul-02	25-Jun-02	28-Jul-03	21-Dec-05	23-May-07

USA							
**Centre number(s) / Investigator(s)**	**IRB or IEC (name/address)**	**IRB or IEC Chairperson**	**Informed Consent/Pt. Information Form Approval DD/MM/YY**	**Protocol Approval DD/MM/YY**	**Am. 1 Approval DD/MM/YY**	**Am. 2 Approval DD/MM/YY**	**Am. 3 Approval DD/MM/YY**
3778 / James Otoshi	Western Institutional Review Board<br>3535 Seventh Avenue, SW<br>Olympia, WA 98502-5010	William C. Jacobs, 2002-2004<br>Theodore D. Schultz, 2005-2008	13-Jun-02	13-Jun-02	28-Jul-03	21-Dec-05	23-May-07
3779 / Rohit Patel	Western Institutional Review Board<br>3535 Seventh Avenue, SW<br>Olympia, WA 98502-5010	William C. Jacobs, 2002-2004<br>Theodore D. Schultz, 2005-2008	09-May-02	09-May-02	28-Jul-03	21-Dec-05	23-May-07
3780 / Allan Pratt	Gunersen Clinic, Ltd.<br>Human Stubjects Committee<br>1836 South Avenue<br>La Crosse, WI 54601	Bernard J. Hammes, 2001 - 2005	24-Apr-02	24-Apr-02	23-Jul-03	15-Dec-05	17-May-07
3781 / Bruce Prenner	Western Institutional Review Board<br>3535 Seventh Avenue, SW<br>Olympia, WA 98502-5010	William C. Jacobs, 2002-2004<br>Theodore D. Schultz, 2005-2008	25-Jul-02	25-Jul-02	28-Jul-03	21-Dec-05	23-May-07
3782 / Jeffery Rehm	Western Institutional Review Board<br>3535 Seventh Avenue, SW<br>Olympia, WA 98502-5010	William C. Jacobs, 2002-2004<br>Theodore D. Schultz, 2005-2008	20-Jun-02	20-Jun-02	28-Jul-03	21-Dec-05	23-May-07

USA

Centre number(s) / Investigator(s)	IRB or IEC (name/address)	IRB or IEC Chairperson	Informed Consent/Pt. Information Form Approval DD/MM/YY	Protocol Approval DD/MM/YY	Am. 1 Approval DD/MM/YY	Am. 2 Approval DD/MM/YY	Am. 3 Approval DD/MM/YY
3783 / Lawrence Repsher	Western Institutional Review Board<br>3535 Seventh Avenue, SW<br>Olympia, WA 98502-5010	William C. Jacobs, 2002-2004<br>Theodore D. Schultz, 2005-2008	11-Apr-02	11-Apr-02	28-Jul-03	21-Dec-05	23-May-07
3784 / Andrew Ries	University California San Diego Human Research Protections Program, 9500 Gilman Drive #0052<br>La Jolla, CA 92093-0052	Daniel Masys, 2003-2007	06-Jun-02	06-Jun-02	07-Aug-03	23-Feb-06	23-May-07
3785 / Karin B. Rock	Western Institutional Review Board<br>3535 Seventh Avenue, SW<br>Olympia, WA 98502-5010	William C. Jacobs, 2002-2004<br>Theodore D. Schultz, 2005-2008	25-Apr-02	25-Apr-02	28-Jul-03	21-Dec-05	23-May-07
3787 / Amir Sharafkhaneh	The Institutional Review Board<br>For Human Subject Research at Baylor College of Medicine and Affiliated Hospitals, Baylor College of Medicine, One Baylor Place, Houston TX 77030	Daniel Epner, 2000<br>Kathleen Motil, 2002-2004<br>Lauren Marangell, 2004	02-Oct-02	02-Oct-02	13-Aug-03	31-Mar-06	22-Jan-08

USA							
**Centre number(s) / Investigator(s)**	**IRB or IEC (name/address)**	**IRB or IEC Chairperson**	**Informed Consent/Pt. Information Form Approval DD/MM/YY**	**Protocol Approval DD/MM/YY**	**Am. 1 Approval DD/MM/YY**	**Am. 2 Approval DD/MM/YY**	**Am. 3 Approval DD/MM/YY**
3788 / Gerry San Pedro	Louisiana State University Health Sciences Center-Shreveport Institutional Review Board<br>1501 Kings Highway<br>Shreveport, LA 71103	Jerry McLarty 2003-2007	26-Sept-02	26-Sept-02	08-Jul-03	05-Jan-06	11-May-07
3789 / Paul D. Scanlon	Mayo Clinic Department of Laboratory Medicine & Pathology<br>200 1st Street S.W<br>Rochester, MN 55905	Louis Letendre, 2002<br>John Pemberton, 2003<br>Randall Pearson, 2004-2006	30-Jan-03	30-Jan-03	07-Jul-03	12-Jan-06	08-Nov-07
3790 / Paul Scheinberg	Western Institutional Review Board<br>3535 Seventh Avenue, SW<br>Olympia, WA 98502-5010	William C. Jacobs, 2002-2004<br>Theodore D. Schultz, 2005-2008	28-Mar-02	28-Mar-02	28-Jul-03	27-Jan-06	23-May-07
3791 / Joe Schelbar	Western Institutional Review Board<br>3535 Seventh Avenue, SW<br>Olympia, WA 98502-5010	William C. Jacobs, 2002-2004<br>Theodore D. Schultz, 2005-2006	09-May-02	09-May-02	28-Jul-03	21-Dec-05	23-May-07
3792 / Jeffery Schul	Western Institutional Review Board<br>3535 Seventh Avenue, SW	William C. Jacobs, 2002-2004<br>Theodore D.	06-Jun-02	06-Jun-02	28 Jul-03	21-Dec-05	23-May-07

USA							
**Centre number(s) / Investigator(s)**	**IRB or IEC (name/address)**	**IRB or IEC Chairperson**	**Informed Consent/Pt. Information Form Approval DD/MM/YY**	**Protocol Approval DD/MM/YY**	**Am. 1 Approval DD/MM/YY**	**Am. 2 Approval DD/MM/YY**	**Am. 3 Approval DD/MM/YY**
	Olympia, WA 98502-5010	Schultz, 2005-2006					
3793 / Jonathan Fine	Norwalk Hospital Institution- al Review Board, Maple Street, Norwalk, CT 06856	Eric Mazur, 2002<br>Lynda Nemeth, 2003-2006	14-Oct-02	14-Oct-02	3-Sept-03	28-Apr-06	07-Jun-07
3794/ 3719 / Segall	Western Institutional Review Board<br>3535 Seventh Avenue, SW<br>Olympia, WA 98502-5010	William C. Jacobs, 2002-2004<br>Theodore D. Schultz, 2005-2008	02-May-02	02-May-02	28-Jul-03	21-Dec-05	23-May-07
3795 / Seibert	Western Institutional Review Board<br>3535 Seventh Avenue, SW<br>Olympia, WA 98502-5010	William C. Jacobs, 2002-2004<br>Theodore D. Schultz, 2005-2008	13-Jun-02	13-Jun-02	28-Jul-03	21-Dec-05	23-May-07
3796 / Serfillippi	Western Institutional Review Board<br>3535 Seventh Avenue, SW<br>Olympia, WA 98502-5010	William C. Jacobs, 2002-2004<br>Theodore D. Schultz, 2005-2008	28-Mar-02	28-Mar-02	28-Jul-03	21-Dec-05	23-May-07
3797 / Spirn	Western Institutional Review Board<br>3535 Seventh Avenue, SW<br>Olympia, WA 98502-5010	William C. Jacobs, 2002-2004<br>Theodore D. Schultz, 2005-	10-Oct-02	10-Oct-02	28-Jul-03	21-Dec-05	23-May-07

USA							
**Centre number(s) / Investigator(s)**	**IRB or IEC (name/address)**	**IRB or IEC Chairperson**	**Informed Consent/Pt. Information Form Approval DD/MM/YY**	**Protocol Approval DD/MM/YY**	**Am. 1 Approval DD/MM/YY**	**Am. 2 Approval DD/MM/YY**	**Am. 3 Approval DD/MM/YY**
		2008					
3799 / Sussman	Western Institutional Review Board<br>3535 Seventh Avenue, SW<br>Olympia, WA 98502-5010	William C. Jacobs, 2002-2004<br>Theodore D. Schultz, 2005-2008	02-May-02	02-May-02	28-Jul-03	21-Dec-05	23-May-07
3800 / Tashkin	Office for Protection of Research Subjects (OPRS)<br>Medical Institutional Review Board<br>1111Ken Ross Ave Suite 102<br>PO BOX 951694<br>Los Angeles, California 90095-1694	Lawrence Wollinsky 2005-07	02-Aug-02	02-Aug-02	12-Sep-03	22-Feb-06	26-Sept-07
3802 / Wachtel	Western Institutional Review Board<br>3535 Seventh Avenue, SW<br>Olympia, WA 98502-5010	William C. Jacobs, 2002-2004<br>Theodore D. Schultz, 2005-2008	06-Jun-02	06-Jun-02	28-Jul-03	21-Dec-05	23-May-07
3804 / Jan. H. Westerman	Western Institutional Review Board<br>3535 Seventh Avenue, SW<br>Olympia, WA 98502-5010	William C. Jacobs, 2002-2004<br>Theodore D. Schultz, 2005-2008	09-May-02	09-May-02	28-Jul-03	21-Dec-05	23-May-07

USA							
**Centre number(s) / Investigator(s)**	**IRB or IEC (name/address)**	**IRB or IEC Chairperson**	**Informed Consent/Pt. Information Form Approval DD/MM/YY**	**Protocol Approval DD/MM/YY**	**Am. 1 Approval DD/MM/YY**	**Am. 2 Approval DD/MM/YY**	**Am. 3 Approval DD/MM/YY**
3806 / Robert A. Wise	Western Institutional Review Board 3535 Seventh Avenue, SW Olympia, WA 98502-5010	William C. Jacobs, 2002-2004 Theodore D. Schultz, 2005-2008	25-Apr-02	25-Apr-02	28-Jul-03	21-Dec-05	23-May-07
3807 / Ronald Zielinski	Western Institutional Review Board 3535 Seventh Avenue, SW Olympia, WA 98502-5010	William C. Jacobs, 2002-2004 Theodore D. Schultz, 2005-2008	13-Jun-02	13-Jun-02	28-Jul-03	Closed 02-Nov-05	Closed 02-Nov-05
3808 / Richard ZuWallack	St. Francis Hospital and Medical Center Institutional Review Board 114 Woodland St. Hartford, CT 06105	Ernesto Canalis 2003-2007	26-Jun-02	26-Jun-02	10-Jul-03	22-Feb-06	9-May-07
3809 / Gary Ferguson	Western Institutional Review Board 3535 Seventh Avenue, SW Olympia, WA 98502-5010	William C. Jacobs, 2002-2004 Theodore D. Schultz, 2005-2006	19-Sep-02	19-Sep-03	28-Jul-03	21-Dec-05	23-May-07
3810 / Arthur Gelb	Western Institutional Review Board 3535 Seventh Avenue, SW Olympia, WA 98502-5010 P.O. Box 12029, Olympia, WA 98508-2029	William C. Jacobs, 2002-2004 Theodore D. Schultz, 2005-2008	29-Aug-02	29-Aug-02	28-Jul-03	21-Dec-05	23-May-07

USA							
**Centre number(s) / Investigator(s)**	**IRB or IEC (name/address)**	**IRB or IEC Chairperson**	**Informed Consent/Pt. Information Form Approval DD/MM/YY**	**Protocol Approval DD/MM/YY**	**Am. 1 Approval DD/MM/YY**	**Am. 2 Approval DD/MM/YY**	**Am. 3 Approval DD/MM/YY**
3811 / Dr. Ware G. Kuschner	Stanford University Administrative Panel of Human Subjects in Medical Research 1215 Welch Road, Modular A Stanford, CA 94395 – 5401	David Oakes, 2002-2006	05-Nov-02	05-Nov-02	5-Aug-03	7-Feb-06	20-Jun-07
3812 / Kees Mahutte	Human Studies Subcomittee VA Long Beach Healthcare System 5901 East 7th Street (09-151) Long Beach, CA 90822	James Hagar, 2002-2003	31-Jan-03	31-Jan-03	10-Jul-03	19-Dec-05	31-May-07
3813 / Fernando Martinez	Medical School Institutional Review Board (IRBMED), 4558 Kresge Medical Research Building, Ann Arbor, MI 48109-0570	Vernon K. Sondak, 2002-2003	14-Nov-02	14-Nov-02	02-Oct-03	09-Feb-06	29-Apr-08
3814 / Mark Plautz	Veterans Affairs Medical Center #151, Attention Human Studies Committee, 4801 East Linwood Blvd., Kansas City MO 64128	James Hamilton, 2004	31-Dec-02	31-Dec-02	10-Jul-03	12-Jan-06	9-May-07
3815 / Carolyn Rochester	VA Connecticut Health System IRB Human Studies Sub-Committee	Robert Kerns, 2002-2003 Morris Bell, 2005	28-Feb-03	28-Feb-03	14-Aug-03	01-Mar-07	7-Jun-07

USA							
**Centre number(s) / Investigator(s)**	**IRB or IEC (name/address)**	**IRB or IEC Chairperson**	**Informed Consent/Pt. Information Form Approval DD/MM/YY**	**Protocol Approval DD/MM/YY**	**Am. 1 Approval DD/MM/YY**	**Am. 2 Approval DD/MM/YY**	**Am. 3 Approval DD/MM/YY**
	Research 1151 Attn. Cora Mileski 950 Campbell Avenue West Haven, CT 06516						
3816 / Gregory Schilero	Bronx Veterans Affairs Medical Center Research Program The Research and Development Office, 151 Room 1F01 130 West Kingsbridge Road Bronx, New York 10468	Lawrence Feinman, 2002-2004 Sheldon Brown, 2004	10-Apr-03	09-Jan-03	Did not submit All patients completed visit 2 prior to amendment	13-Jul-06	07-Jun-07
3817 / Gregory Tino	University of Pennsylvania Mezzaine Level, Mellon Bank Building, 133 S. 36th Street, Philadelphia, PA 19104	Does not closure the Chairman's name but meets the ICH.GCP guidelines	15-Jan-03	15-Jan-03	9-Jul-03	18 Dec-06	Closed
3818 / Gerard Turino	St. Luke's – Roosevelt Hospital Center, Institutional Review Board, 432 West 58th Street – Room 207, New York, New York 10019	Airlie Cameron 2003-2007	16-Dec-02	16-Dec-02	18-Nov-03	8-May-06	23-May-07
3820 / John Keppel	Western Institutional Review Board 3535 Seventh Avenue, SW, Olympia, WA 98502-5010, P.O. Box 12029,	William C. Jacobs, 2002-2004 Theodore D. Schultz, 2005-	19-Sept-02	19-Sept-02	28-Jul-03	21-Dec-05	23-May-07

USA							
**Centre number(s) / Investigator(s)**	**IRB or IEC (name/address)**	**IRB or IEC Chairperson**	**Informed Consent/Pt. Information Form Approval DD/MM/YY**	**Protocol Approval DD/MM/YY**	**Am. 1 Approval DD/MM/YY**	**Am. 2 Approval DD/MM/YY**	**Am. 3 Approval DD/MM/YY**
	Olympia, WA, 98508-2029	2008					
